# Synthesis of 1,4-benzothiazinones from acylpyruvic acids or furan-2,3-diones and *o*-aminothiophenol

**DOI:** 10.3762/bjoc.16.193

**Published:** 2020-09-21

**Authors:** Ekaterina E Stepanova, Maksim V Dmitriev, Andrey N Maslivets

**Affiliations:** 1 Department of Chemistry, Perm State University, ul. Bukireva 15, Perm 614990, Russian Federation

**Keywords:** acylpyruvic acid, 1,4-benzothiazine, cyclocondensation, diversity-oriented synthesis, furan-2,3-dione

## Abstract

Two synthetic approaches to enaminones fused to 1,4-benzothiazin-2-one moiety, which can be interesting in studies on biological activity, chemosensors, and fluorescence, were developed via the reaction of furan-2,3-diones or acylpyruvic acids in the presence of carbodiimides with *o*-aminothiophenols. The target enaminones were formed together with pharmaceutically interesting 2-hydroxy-2*H*-1,4-benzothiazin-3(4*H*)-ones. A selective synthetic approach to 2-hydroxy-2*H*-1,4-benzothiazin-3(4*H*)-ones was developed via the solvent-switchable reaction of furan-2,3-diones with *o*-aminothiophenol. Preliminary biological assays (antimicrobial, acute toxicity) of the new compounds were carried out.

## Introduction

**Figure 1:**
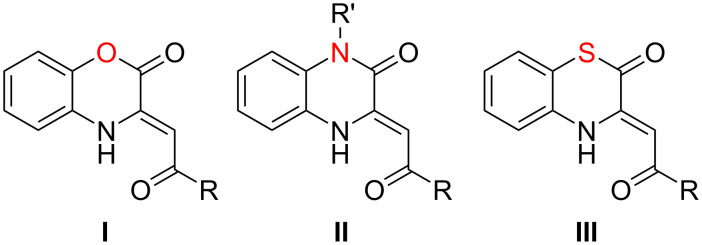
Enaminones fused to heterocyclic moieties.

Enaminones fused to the 1,4-benzoxazine-2-one **I** or quinoxaline-2(1*H*)-one **II** moiety (Figure 1) represent an intensively investigated class of enamines. Undying interest in these compounds is due to the simplicity of their synthesis and purification, the availability of starting materials and the possibility of their synthesis under mild green conditions [1–6]. Because of their availability in gram-scale quantities, these compounds were thoroughly investigated for the possibility of practical use. Particularly, some enaminones **I** and **II** were found to show antioxidant [3], antimycotic [7–8], antimycobacterial [9–10], anti-Alzheimer’s disease (JNK3 inhibitors) [11], platelet aggregation inhibitory [12], antimicrobial [7] and analgesic [13] activities. Enaminones **II** were reported as potential Cu^2+^ chemosensors [14], and their BF_2_ chelates, as multicolor fluorescence complexes, some of which exhibited aggregation-induced emission (AIE) properties [15]. In addition, enaminones **I** and **II** proved themselves to be versatile and available building blocks for the synthesis of various heterocyclic systems [10,16–20].

**Scheme 1:**
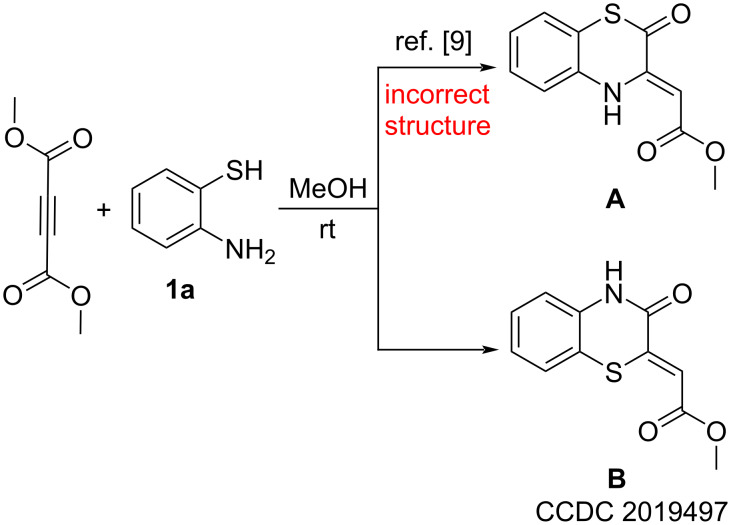
Reported structure **A** of assayed compound [9] and its correct structure **B**.

In light of the unquestionable practical value of the above-mentioned 1-oxa- and 1-aza-substituted enaminones **I** and **II**, investigations on the properties of their 1-thia analogs, the 1,4-benzothiazin-2-ones (BTAs) **III**, (Figure 1) seem to be promising. But according to the literature, data on BTAs **III** have been poorly reported. For example, the only available report [9] on their antimycobacterial activity gives erroneous information, since the structure **A** of the assayed compound has been established incorrectly (Scheme 1). We reproduced the synthesis from the work [9] and found that the correct structure of the studied compound was structure **B** (CCDC 2019497) (Scheme 1).

**Scheme 2:**
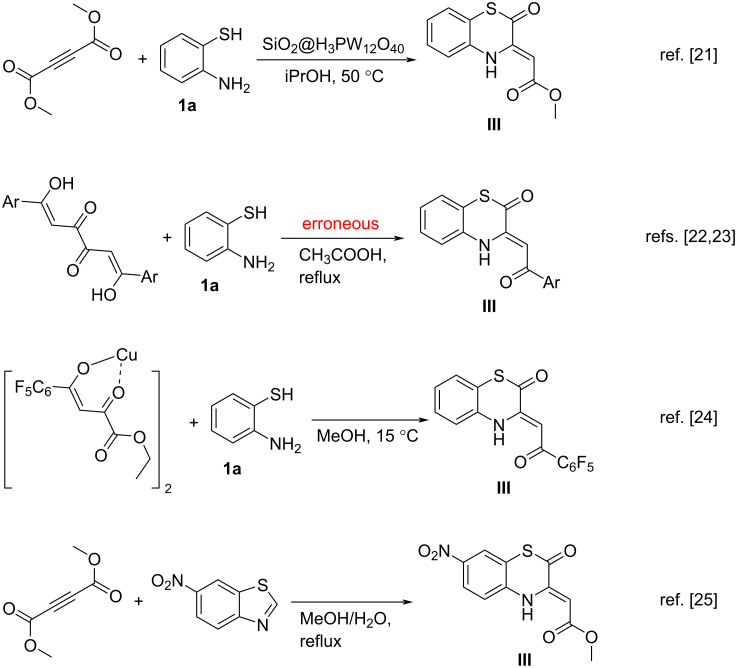
Known synthetic approaches to BTAs **III**.

In fact, for now, four approaches to BTAs **III** are known [21–25] (Scheme 2). They include the reaction of dimethyl acetylenedicarboxylate (DMAD) with *o*-aminothiophenol (**1a)** on the catalyst SiO_2_@H_3_PW_12_O_40_ [21], the reaction of tetracarbonyl compounds with *o*-aminothiophenol (**1a**) [22–23], the reaction of copper(II) chelate of ethyl pentafluorobenzoylpyruvate with *o*-aminothiophenol (**1a**, one example) [24] and the reaction of DMAD with 6-nitro-1,3-benzothiazole (one example) [25]. Our recent research [26] revealed that the approach based on the reaction of tetracarbonyl compounds with *o*-aminothiophenol (**1a**) [22–23] is a mistaken one. Considering these facts, it can be concluded that there are no convenient synthetic approaches with a wide substrate scope to BTAs **III**.

Herein, we report a comprehensive research on synthetic approaches to BTAs **III**.

## Results and Discussion

**Scheme 3:**
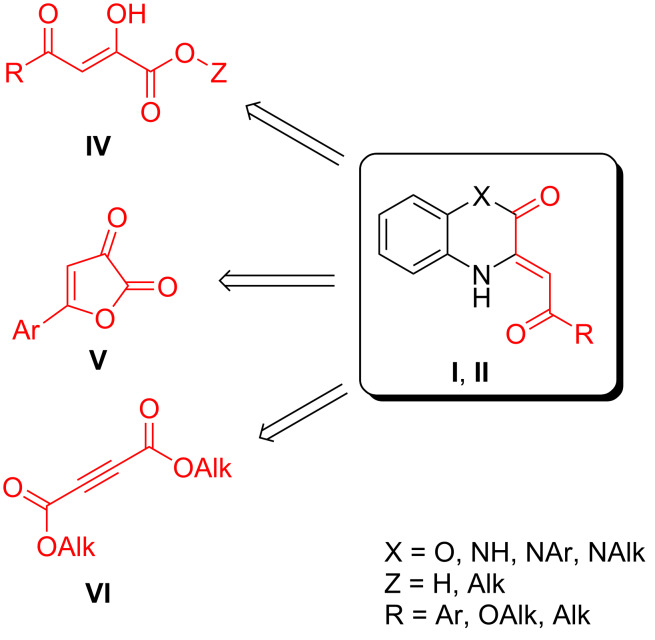
General synthetic approaches to enaminones **I** and **II**.

To develop an approach to target the BTAs **III**, we analyzed the general synthetic methods to enaminones **I** and **II**, which involve the reaction of the acylpyruvic acids or their esters **IV**, 5-arylfuran-2,3-diones **V**, and acetylenedicarboxylates **VI** with *o*-aminophenols or *o*-phenylenediamines (Scheme 3) [1–6]. Since the approach to alkoxy-substituted BTAs **III** (Figure 1, R = OAlk) has already been reported (Scheme 2) [21], we focused our research on aryl/alkyl-substituted BTAs **III** (Figure 1, R = Ar, Alk).

By analogy with the synthesis of enaminones **I** and **II**, it can be assumed that the target aryl/alkyl-substituted BTAs **III** can be accessible via the reaction of acylpyruvic acids or their esters **IV** and 5-arylfuran-2,3-diones **V** with *o*-aminothiophenol (**1a**). But in fact, this assumption is not correct.

**Scheme 4:**
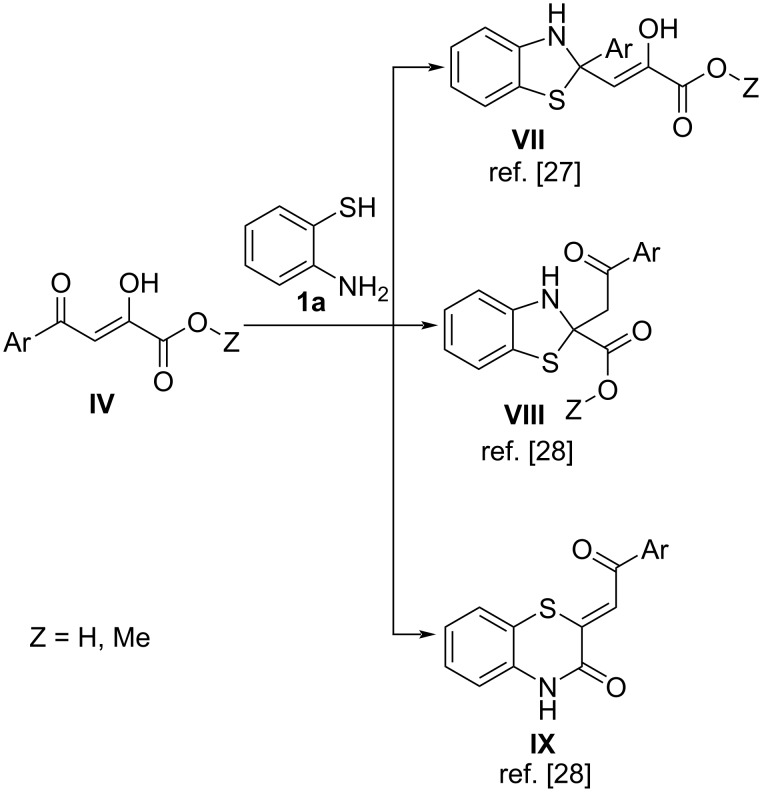
Reported reactions of acylpyruvic acids or their esters **IV** with *o*-aminothiophenol (**1a**) [27–28].

The approach based on the reaction of acylpyruvic acids or their esters **IV** with *o*-aminothiophenol (**1a**) can be supposed as the simplest and expedite, but it was reported to afford other types of products **VII**–**IX** (Scheme 4) [27–28] instead of the desired BTAs **III**.

To verify the unsuitability of this approach, we repeated some experiments described previously [27–28] and examined the reaction mixtures by UPLC–UV–MS. We supposed that the peak of the target BTA **III** would be characterized by appropriate molecular ion signals at ESI^+^ and ESI^−^ chromatograms and absorbance bands at 400–450 nm (representative for enaminones **I** and **II** [28]) at UV chromatograms. The examined reaction mixtures showed peaks characteristic for adducts **VII**–**IX**, and no peaks characteristic for target BTAs **III** were observed, which was in good agreement with previous studies [27–28].

The inability of the reaction of acylpyruvic acids or their esters **IV** with *o*-aminothiophenol (**1a**) to afford the target BTAs **III** can be explained both by the impossibility of thioesterification/thiotransesterification to proceed under additives-free conditions and by the high nucleophilicity of the *o*-aminothiophenol’s SH group that attacked the more electrophilic ketone carbonyls of compounds **IV** firstly. To overcome these issues, we examined the reaction of acylpyruvic acid (**2a**) with *o*-aminothiophenol (**1a**) in the presence of carbodiimides in various solvents (Table 1). Fortunately, in many cases we succeeded to detect the desired BTA **3a** by UPLC–UV–MS. The target product **3a** was formed along with the concurrent product **4a** (Table 1). Both products **3a** and **4a** were isolated and characterized, and their structures were unequivocally proved by single crystal X-ray analyses (**3a**, CCDC 2019498; **4a**, CCDC 2019501).

**Table 1:** Reaction of acylpyruvic acid **2a** with *o*-aminothiophenol (**1a**) in the presence of carbodiimides in various solvents.

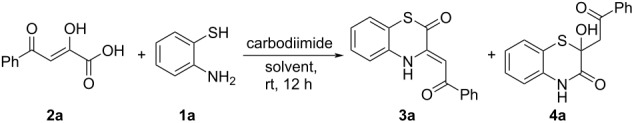					

Entry	Solvent	Carbodiimide^a^	Procedure^b^	Yield^c^, %	

				**3a**	**4a**

1	toluene	DCC	A	35	23
2	toluene	DCC	B	21	14
3	toluene	DIC	A	33	21
4	toluene	DIC	B	18	16
5	DMSO	DCC	A	traces	18
6	DMSO	DCC	B	traces	44
7	1,4-dioxane	DCC	A	25	24
8	1,4-dioxane	DCC	B	22	27
9	acetonitrile	DCC	A	18	8
10	acetonitrile	DCC	B	56	34
11	chloroform	DCC	A	traces	20
12	chloroform	DCC	B	traces	27
13	DMF	DCC	A	7	35
14	DMF	DCC	B	18	54
15	ethyl acetate	DCC	A	25	21
16	ethyl acetate	DCC	B	30	35
17	tetrahydrofuran	DCC	A	31	16
18	tetrahydrofuran	DCC	B	25	21
19	hexane	DCC	A	traces	traces
20	hexane	DCC	B	traces	traces
21	acetone	DCC	A	28	18
22	acetone	DCC	B	33	20
23	*N*-methyl-2-pyrrolidone	DCC	A	3	35
24	*N*-methyl-2-pyrrolidone	DCC	B	11	54
25	acetonitrile	DCC	C	52	30
26	acetonitrile	DIC	C	53	33
27	1,4-dioxane	DCC	C	19	46
28	DMF	DCC	C	34	24
29	acetonitrile	DCC	D	–	traces
30	acetonitrile	EDC	B	–	–

^a^DCC – dicyclohexylcarbodiimide, DIC – diisopropylcarbodiimide, EDC – 1-ethyl-3-(3-dimethylaminopropyl)carbodiimide. ^b^Procedure A: to a stirring suspension of compound **2a** (10 mg, 52 µmol) and *o*-aminothiophenol (**1a**, 7 µL, 65 µmol) in a solvent (100 µL), a carbodiimide (52 µmol) was added; procedure B: to a stirring suspension of compound **2a** (10 mg, 52 µmol) and a carbodiimide (52 µmol) in a solvent (100 µL), *o*-aminothiophenol (**1a**, 7 µL, 65 µmol) was added; procedure C: to a stirring suspension of compound **2a** (10 mg, 52 µmol) in a solvent (100 µL), a carbodiimide (52 µmol) was added, and right after, *N*-hydroxybenzotriazole (HOBt) hydrate (8 mg, 52 µmol) was added, and after 5 min, *o*-aminothiophenol (**1a**, 7 µL, 65 µmol) was added to the reaction mixture; procedure D: to a stirring suspension of compound **2a** (10 mg, 52 µmol) in a solvent (100 µL), a carbodiimide (52 µmol) and 4-dimethylaminopyridine (DMAP, 6.4 mg, 52 µmol) were added, and after 5 min, *o*-aminothiophenol (**1a**, 7 µL, 65 µmol) was added to the reaction mixture. ^c^UPLC–UV yields (biphenyl was used as an internal standard; each entry was carried out in triplicate, and the yields are given as mean values).

During the optimization (Table 1), we found that the yield of BTA **3a** depended on the method of the addition of the reagents (Table 1, procedure A or B). In case of some non-polar solvents (toluene, 1,4-dioxane), the addition of *o*-aminothiophenol (**1a**) before the carbodiimide resulted in higher yields of BTA **3a**, and in case of some polar solvents (acetonitrile, ethyl acetate, DMF, and *N*-methyl-2-pyrrolidone), addition of the carbodiimide before *o*-aminothiophenol (**1a**) afforded higher yields of BTA **3a**.

Noteworthy, the solvents strongly influenced the studied reaction. When DMSO was used as a solvent (Table 1, entries 5 and 6), only trace amounts of the target compound **3a** were formed, probably, because the combination of DMSO and a carbodiimide induced multiple side-processes similar to the Pfitzner–Moffatt oxidation [29]. In case of hexane as a solvent (Table 1, entries 19 and 20), only traces of compounds **3a** and **4a** were observed, because of the low solubility of benzoylpyruvic acid (**2a**) and *o*-aminothiophenol (**1a**) in hexane. To our surprise, carrying out the reaction in chloroform (Table 1, entries 11 and 12) resulted in the formation of multiple side-products and only traces of BTA **3a**. When acetone was used as a solvent (Table 1, entries 21 and 22), though the target BTA **3a** was formed in about 30% yield, side-products were observed, which, possibly, were formed due to the reaction of acetone with *o*-aminothiophenol (**1a**). The best yield of BTA **3a** was observed in acetonitrile (Table 1, entry 10).

Then we tested the effect of common additives for carbodiimide coupling reactions, DMAP [30] and HOBt [31]. The utilization of HOBt (Table 1, entries 25 and 26) in acetonitrile as an additive did not significantly influence the yield of BTA **3a**. But since the solubility of HOBt in acetonitrile is poor [32], we examined the reaction in DMF and 1,4-dioxane (Table 1, entries 27 and 28), which are known to be better solvents for HOBt [32]. Unfortunately, these attempts did not provide any significant results either.

When DMAP (Table 1, entry 29) was employed, the formation of BTA **3a** was not observed at all, and the side-product **4a** was formed only in trace amounts. This phenomenon can be explained by the formation of intermediates highly sensitive to amines.

**Scheme 5:**
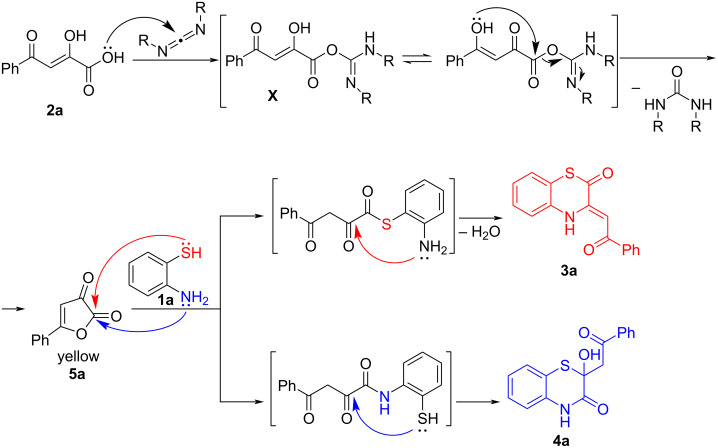
Plausible mechanism of the reaction of acylpyruvic acid **2a** with *o*-aminothiophenol (**1a**) in the presence of carbodiimides.

We believe that the reaction of acylpyruvic acid **2a** with *o*-aminothiophenol (**1a**) in the presence of carbodiimides proceeded through the formation of 5-phenylfuran-2,3-dione (**5a**, CCDC 2019499, Scheme 5).

The acid **2a** reacted with a carbodiimide to form the reactive isourea derivative **X** which underwent intramolecular cyclization with the formation of furandione **5a** which reacted with *o*-aminothiophenol (**1a**). Product **3a** was formed when the *o*-aminothiophenol’s SH group attacked the compound **5a** at the lactone carbonyl group, and the product **4a**, when *o*-aminothiophenol’s NH_2_ group attacked compound **5a** at the lactone carbonyl group. The in situ formation of 5-phenylfuran-2,3-dione (**5a**) was indicated by the appearance of bright yellow color (characteristic for the 5-arylfuran-2,3-diones **5** [6]) after mixing the suspension of acid **2a** with a carbodiimide. In addition, after mixing the suspension of acid **2a** in acetonitrile with DIC, the formation of the yellow precipitate of 5-phenylfuran-2,3-dione (**5a**) was observed, and compound **5a** could be isolated by simple filtration. Moreover, after mixing the suspension of acid **2a** with DCC, formation of the white precipitate of dicyclohexylurea was observed, which gave the evidence of intramolecular cyclization to furandione **5a** with dicyclohexylurea elimination occurring. The formation of furandione **5a** explained well our observations when DMAP was used as an additive (Table 1, entry 29), since furandiones **5** are well-known to readily react with amines to afford various products [33]. To confirm our assumption, we examined the reaction of furandione **5a** with DMAP. For this, equimolar amounts of compound **5a** and DMAP were stirred at room temperature in acetonitrile for 1 h, and the reaction mixture was examined by UPLC–UV–MS. As a result, a complex mixture of various products was observed. Similar results were obtained upon the treatment of compound **5a** with triethylamine under the same conditions. These meant that 5-arylfuran-2,3-diones **5** were sensitive to tertiary amines and should only be carefully treated with reagents containing tertiary amino groups.

Because of this, we were encouraged to examine EDC as a carbodiimide in the developed procedure (Table 1, entry 30). As expected, we found that the utilization of EDC in this reaction resulted in the formation of a complex mixture containing no target products **3a** and **4a**. So, EDC cannot be utilized as an activating additive in the studied reaction, while DCC and DIC could be used equally.

Taking these results into account, we examined the direct reaction of furandione **5a** with *o*-aminothiophenol (**1a**) in various solvents (Table 2). As we expected, the studied process afforded compounds **3a** and **4a**. The best yields of BTA **3a** were observed in acetonitrile (Table 2, entries 4 and 5). Notably, an increase in the reaction temperature (Table 2, entry 6) resulted in a decrease of the BTA **3a** yield, which could possibly mean, that BTA **3a** was the kinetic product. Unfortunately, conduction of the reaction at −40 °C (Table 2, entry 5) did not increase the BTA **3a** yield.

**Table 2:** Reaction of furandione **5a** with *o*-aminothiophenol (**1a**) in various solvents.^a^

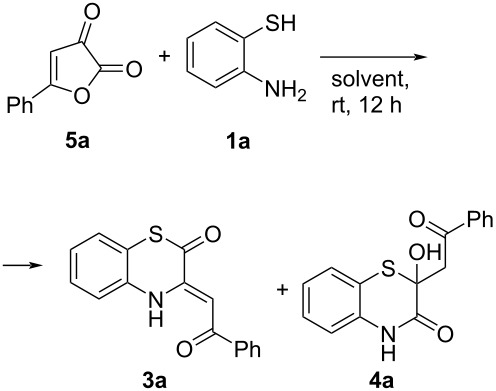			

Entry	Solvent	Yield^b^, %	

		**3a**	**4a**

1	toluene	traces	79
2	DMSO	traces	82
3	1,4-dioxane	12	80
4	acetonitrile	41	55
5	acetonitrile^c^	42	50
6	acetonitrile^d^	20	55
7	chloroform	traces	79
8	DMF	21	72
9	ethyl acetate	25	68
10	tetrahydrofuran	15	69
11	acetic acid	19	69
12	acetone	21	50
13	*N*-methyl-2-pyrrolidone	15	81

^a^To a stirring suspension of furandione **5a** (10 mg, 57 µmol) in solvent (100 µL), *o*-aminothiophenol (**1a**, 7 µL, 65 µmol) was added. ^b^UPLC–UV yields (biphenyl was used as an internal standard; each entry was carried out in triplicate, and the yields are given as mean values). ^c^The reaction was carried out at −40 °C. ^d^The reaction was carried out at 70 °C.

Thus, we found two approaches to the target BTA **3a**. These two approaches could be used as interchangeable in the synthesis of BTAs **3**. The furandione-based approach gave less side products, but the yield of the BTA **3** was lower and, sometimes, furandiones **5** were difficult to be synthesized [2]. The acylpyruvic acid-based approach gave higher yields of the target compounds **3**, involved available reagents, but produced more waste. In fact, the acylpyruvic acid-based approach could be considered as a furandione-based one with in situ generation of furandiones **5**.

**Scheme 6:**
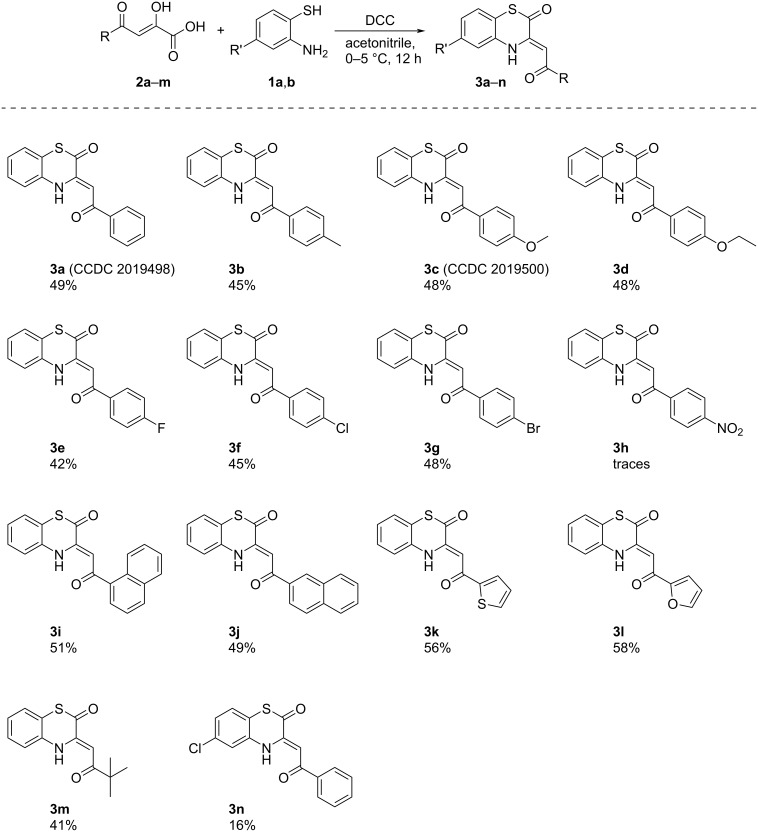
The substrate scope of the optimized approach to BTAs **3a–n**. Procedure: to a cooled to 0–5 °C stirring suspension of compound **2** (5 mmol) and DCC (5 mmol) in acetonitrile (10 mL), *o*-aminothiophenol **1** (5.1 mmol) was added; Isolated yields are shown.

Since the acylpyruvic acid-based approach gave higher yields of the target compounds **3**, we investigated its substrate scope by involvement of various acylpyruvic acids **2a–m** and *o*-aminothiophenols **1a,b** (Scheme 6). It should be noted, that scaled up reactions of acylpyruvic acids **2a–m** and *o*-aminothiophenols **1a,b** in the presence of DCC was found to be exothermic, therefore, cooling was applied.

As a result, we found that the developed procedure to BTAs **3a**–**n** worked well with a wide range of substituents on the acylpyruvic acids **2**, excepting 4-nitrobenzoylpyruvic acid (**2h**). In case of acid **2h**, target BTA **3h** was formed only in trace amounts detected by UPLC–UV–MS. Our attempts to obtain BTA **3h** through the alternative approach based on the reaction of furandione **5h** with *o*-aminothiophenol (**1a**) were unsuccessful. In both cases, a complex mixture of inseparable products was formed. Such a change in reactivity could be explained by the influence of the NO_2_ substituent in compounds **2h** and **5h**, which had a strong negative mesomeric effect, affecting the whole conjugated bond system in these molecules.

**Scheme 7:**
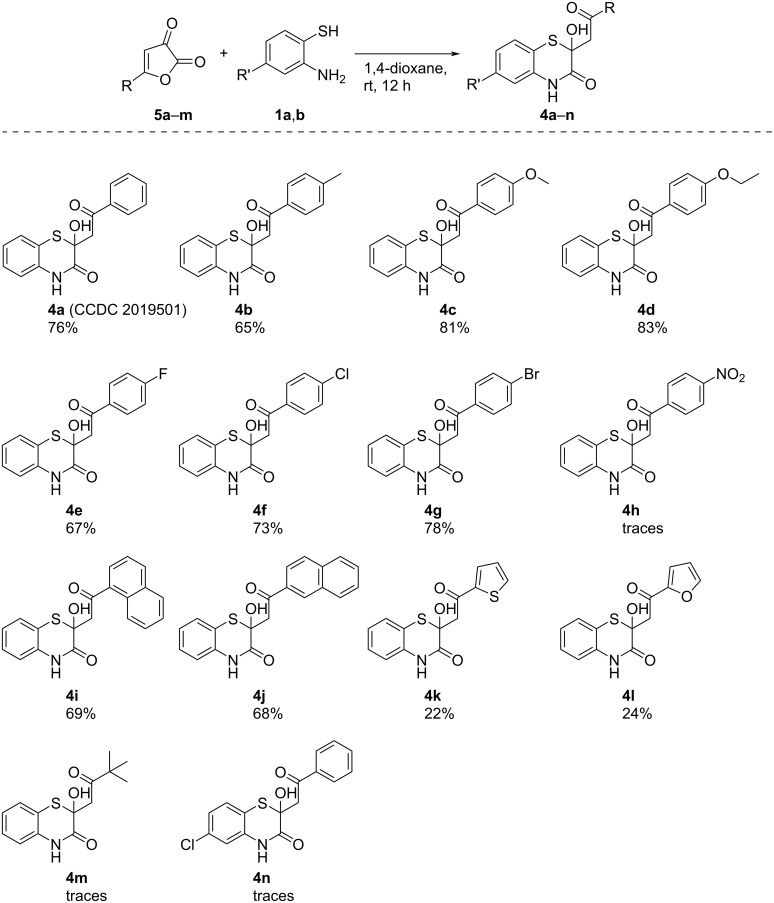
The substrate scope of the optimized approach to compounds **4a–n**. Procedure: to a stirring solution of compound **5** (1 mmol) in 1,4-dioxane (3 mL), *o*-aminothiophenol **1** (1.05 mmol) was added. Isolated yields are shown.

Notably, the target compounds **3a**–**n** were formed together with the corresponding compounds **4a**–**n**, which can be interesting for pharmaceutics, since compounds bearing a 2-hydroxy-2*H*-1,4-benzothiazin-3(4*H*)-one moiety were reported to be potentially useful for treatment of circulatory diseases [34–36]. According to the optimization data, the best yields of compound **4a** were observed at the reaction of furandione **5a** with *o*-aminothiophenol (**1a**) in DMSO or 1,4-dioxane (Table 2, entries 2 and 3). In order to check the substrate scope for compounds **4a–n**, we examined the reactions of various furandiones **5a–m** and *o*-aminothiophenols **1a,b** (Scheme 7).

**Scheme 8:**
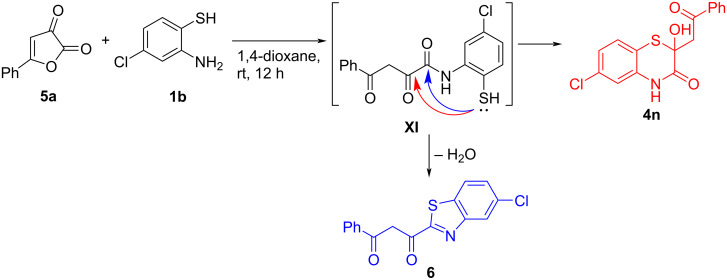
Plausible scheme of the formation of diketone **6**.

The examined procedure to compounds **4a–n** worked well with a wide range of substituents in furandiones **5**. The involvement of 4-nitrophenylfurandione **5h** in the reaction afforded only trace amounts of compound **4h** possibly because of the above mentioned reasons. A similar situation was observed with *tert*-butylfurandione **5m**. Notably, in case of the reaction of phenylfurandione **5a** with chloro-substituted *o*-aminothiophenol **1b**, diketone **6** [26] was isolated as the product instead of the expected compound **4n** (Scheme 8). Diketone **6** was formed when the SH group of intermediate **XI** intramolecularly attacked at the amide carbonyl group (Scheme 8). Such a change in the intramolecular cyclization direction, possibly, was due to the electron-withdrawing effect of the chloro-substituent in the *o*-aminothiophenol moiety, which decreased the nucleophilicity of the SH group, inducing its attack on the carbonyl group bearing a higher partially positive charge (for partial charges comparison, see Supporting Information File 1).

As we were most interested in the biological activity of BTAs **3**, preliminary antimicrobial and toxicity assays of these compounds were carried out (detailed data on biological assays is given in Supporting Information File 1). Unfortunately, we found that tested BTAs **3** did not show any significant antimicrobial activity (against *Staphylococcus aureus*, *Escherichia coli*, *Klebsiella pneumoniae*, *Acinetobacter baumannii*, *Pseudomonas aeruginosa*, *Candida albicans*, *Cryptococcus neoformans var. grubii*, *Mycobacterium tuberculosis*) in vitro. Acute toxicity of BTA **3a** in mice was determined to be higher than 1000 mg/kg, which means that BTA **3a** can be considered as low toxic.

## Conclusion

In conclusion, we developed two interchangeable approaches to enaminones fused to 1,4-benzothiazin-2-one moieties, which may be interesting for studies on biological activity, chemosensors, and fluorescence. The first approach is based on the reaction of furan-2,3-diones **5** with *o*-aminothiophenols **1** in acetonitrile. This approach gave less side products, but the yields of BTAs **3** were lower and, sometimes, starting furandiones **5** are difficult to be synthesized. The second one is based on the reaction of acylpyruvic acids **2** with *o*-aminothiophenols **1** in the presence of carbodiimides in acetonitrile. This approach gave higher yields of the target compounds **3**, involves available precursors, but produced more waste. The acylpyruvic acid-based approach can be considered as furandione-based one with in situ generation of furandiones **5**. The BTAs **3** were formed together with the corresponding 2-hydroxy-2*H*-1,4-benzothiazin-3(4*H*)-ones **4**, which can be interesting for pharmaceutics. A selective synthetic approach to compounds **4** was developed based on the reaction of furan-2,3-diones **5** with *o*-aminothiophenols **1** in 1,4-dioxane or DMSO. According to antimicrobial assays, BTAs **3** did not show any significant antimicrobial activity in vitro. BTA **3a** was found to be low toxic according to the test of acute toxicity in mice.

## Supporting Information

File 1 Experimental details, copies of NMR spectra, X-ray crystallographic details, detailed antimicrobial and toxicity assays, results of semi-empirical calculations of partial charges.
