# Supplementary material for: Synthesis of 1,4-benzothiazinones from acylpyruvic acids or furan-2,3-diones and o-aminothiophenol
Source: Beilstein J Org Chem. 2020 Sep 21;16:2322–31. doi: 10.3762/bjoc.16.193 (PMC7522460; doi:10.3762/bjoc.16.193)
Supplement: File 1 — Experimental details, copies of NMR spectra, X-ray crystallographic details, detailed antimicrobial and toxicity assays, results of semi-empirical calculations of partial charges. [file Beilstein_J_Org_Chem-16-2322-s001.pdf]

BEILSTEIN JOURNAL OF ORGANIC CHEMISTRY

Supporting Information

for

Synthesis of 1,4-benzothiazinones from acylpyruvic acids or furan-2,3-diones and *o*-aminothiophenol

Ekaterina E. Stepanova, Maksim V. Dmitriev and Andrey N. Maslivets

Beilstein J. Org. Chem. **2020,** *16,* 2322–2331. doi:10.3762/bjoc.16.193

Experimental details, copies of NMR spectra, X-ray crystallographic details, detailed antimicrobial and toxicity assays, results of semi-empirical calculations of partial charges

License and Terms: This is a supporting information file under the terms of the Creative Commons Attribution License (https://creativecommons.org/licenses/by/4.0). Please note that the reuse, redistribution and reproduction in particular requires that the authors and source are credited.
The license is subject to the *Beilstein Journal of Organic Chemistry* terms and conditions: (https://www.beilstein-journals.org/bjoc)

Table of contents

General……S3
Methyl (2*Z*)-(3-oxo-3,4-dihydro-2*H*-1,4-benzothiazin-2-ylidene)acetate (**B**)……………………………………………S4
Enaminones fused to the 1,4-benzothiazin-2-one moiety (BTAs) **3a-n**. General procedure…………………………………..S7
2-Hydroxy-2*H*-1,4-benzothiazin-3(4*H*)-ones **4a-n**. General procedure……………………………………………………..S40
1-(5-Chloro-1,3-benzothiazol-2-yl)-3-hydroxy-3-phenylprop-2-en-1-one (**6**)……………………………………………...S69
Antimicrobial assay by CO-ADD (ESKAPE pathogens)………………………………………………………..S72
Antitubercular assays………………………………………………………………………………………………S75
Acute toxicity in mice………………………………………………………………………………………………S78
Crystal structure determination…………………………………………………………………………………..S79
Partial atomic charges comparison in intermediate **XI**…………………………………………………………………S88
References……..S89

Experimental

General

^{1}H, ^{13}C NMR, and ^{19}F NMR spectra were acquired on a Bruker Avance-III spectrometer (400, 100, and 377 MHz, respectively) in $CDCl_3$ or DMSO-d_6 using the HMDS signal (in ^{1}H NMR), solvent residual signals (in ^{13}C NMR, 77.00 for $CDCl_3$, 39.51 for DMSO-d_6; in ^{1}H NMR, 7.27 for $CDCl_3$, 2.50 for DMSO-d_6) or C_6F_6 signal (in ^{19}F NMR) as internal standards. IR spectra were recorded on a Perkin–Elmer Spectrum Two spectrometer from mulls in mineral oil. Melting points were measured on a Khimlabpribor PTP apparatus or a Mettler Toledo MP70 apparatus. Single crystal X-ray analyses were performed on an Xcalibur Ruby diffractometer. Elemental analyses were carried out on a Vario MICRO Cube analyzer. The reaction conditions were optimized using UPLC–UV–MS [Waters ACQUITY UPLC I-Class system; Acquity UPLC BEH C18 column, grain size of 1.7 μm; acetonitrile–water as eluents; flow rate of 0.6 mL/min; ACQUITY UPLC PDA eλ Detector (wavelength range of 230–780 nm); Xevo TQD mass detector; electrospray ionization; positive and negative ion detection; ion source temperature of 150 °C; capillary voltage of 3500–4000 V; cone voltage of 20–70 V; vaporizer temperature of 200 °C. Thin-layer chromatography (TLC) was performed on Merck silica gel 60 F_{254} plates using EtOAc/toluene, 1:5 v/v, toluene, EtOAc as eluents.

Acylpyruvic acids **2** and their esters were obtained from commercially available reagents by the Claisen condensation of corresponding methylketones and diethyl oxalate [1]. Furan-2,3-diones **5** were obtained according to the reported procedures [2] from aroylpyruvic acids **2** and commercially available reagents. Toluene, THF, benzene, and 1,4-dioxane were distilled over Na before use. Ethyl acetate, chloroform, and acetone were distilled over P_2O_5 before the use. Acetonitrile, DMSO, DMF were dried over molecular sieves 4 Å before the use. All other solvents and reagents were purchased from commercial vendors and were used as received.

Methyl (2*Z*)-(3-oxo-3,4-dihydro-2*H*-1,4-benzothiazin-2-ylidene)acetate (B) [3]

To a stirring solution of dimethyl acetylenedicarboxylate (DMAD) (165 µL, 1 mmol) in methanol (5 mL), *o*-aminothiophenol (**1a**, 105 µL, 1 mmol) was added. The mixture was stirred overnight. The formed yellow precipitate was filtered off and recrystallized from 1,4-dioxane to afford methyl (2*Z*)-(3-oxo-3,4-dihydro-2*H*-1,4-benzothiazin-2-ylidene)acetate (**B**).

Yield: 220 mg (94%); bright yellow solid; mp 259–261 °C.
^{1}H NMR (400 MHz, DMSO-d_6): δ = 11.47 (s, 1 H), 7.40 (m, 1 H), 7.24 (m, 1 H), 7.13 (m, 1 H), 7.08 (m, 1 H), 6.92 (s, 1 H), 3.73 (s, 3 H) ppm.
^{13}C NMR (100 MHz, DMSO-d_6): δ = 165.7, 154.1, 140.9, 132.6, 127.1, 125.1, 123.3, 117.0, 114.9, 113.7, 51.5 ppm.
IR (mineral oil): 3178, 1680, 1662 cm^{-1}.
MS (ESI+): m/z calcd for $C_{11}H_9NO_3S+H^+$: 236.04 [M+H$^+$]; found: 235.99.
Anal. Calcd (%) for $C_{11}H_9NO_3S$: C 56.16; H 3.86; N 5.95. Found: C 56.01; H 3.88; N 5.91.
λ_{max} = 376 nm.

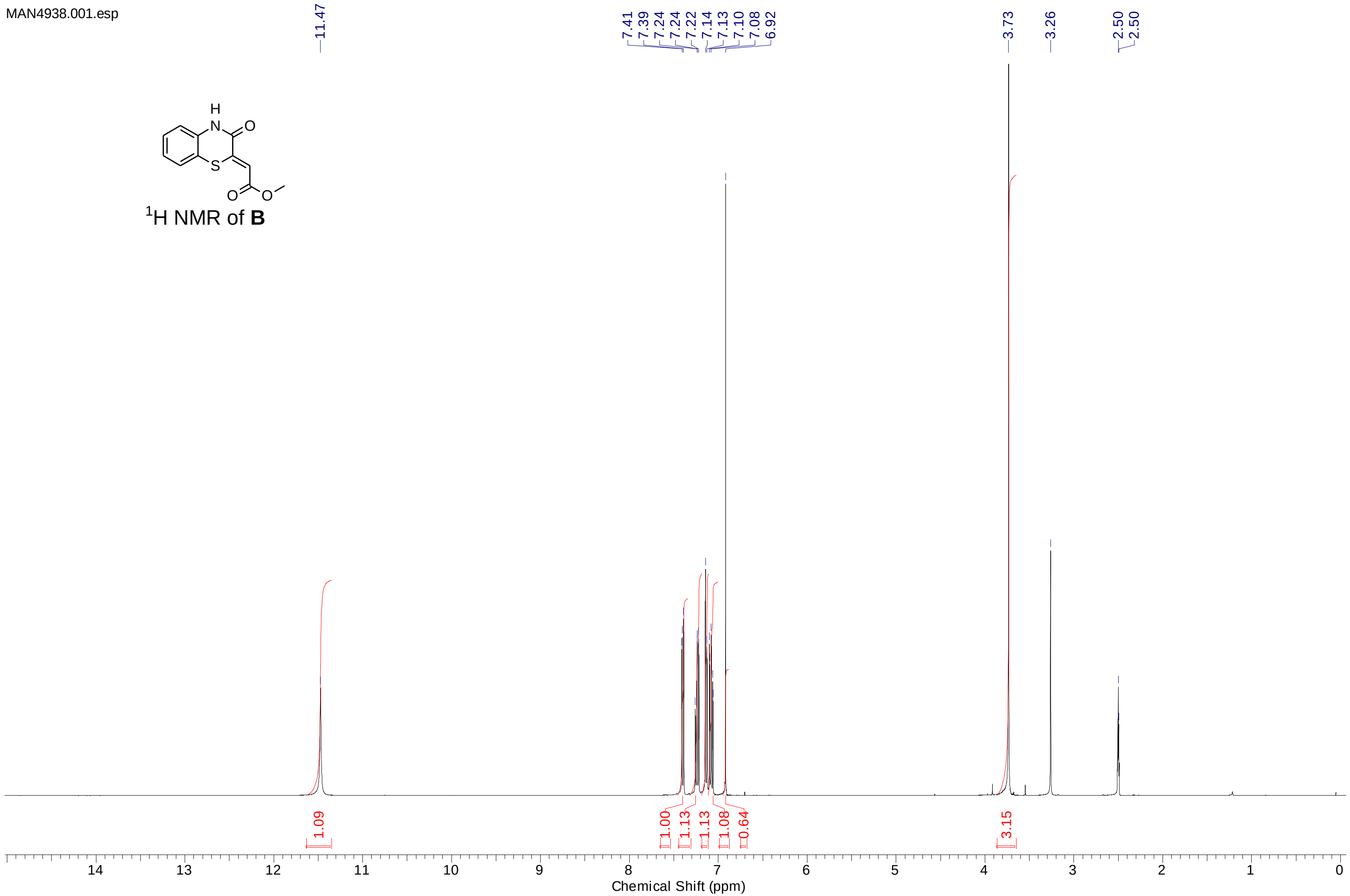

MAN4938.001.esp
11.47
7.41
7.39
7.24
7.24
7.22
7.14
7.13
7.10
7.08
6.92
3.73
3.26
2.50
2.50
H
N
O
S
O
O
^{1}H NMR of **B**
1.09
1.00
1.13
1.13
1.08
0.64
3.15
14 13 12 11 10 9 8 7 6 5 4 3 2 1 0
Chemical Shift (ppm)

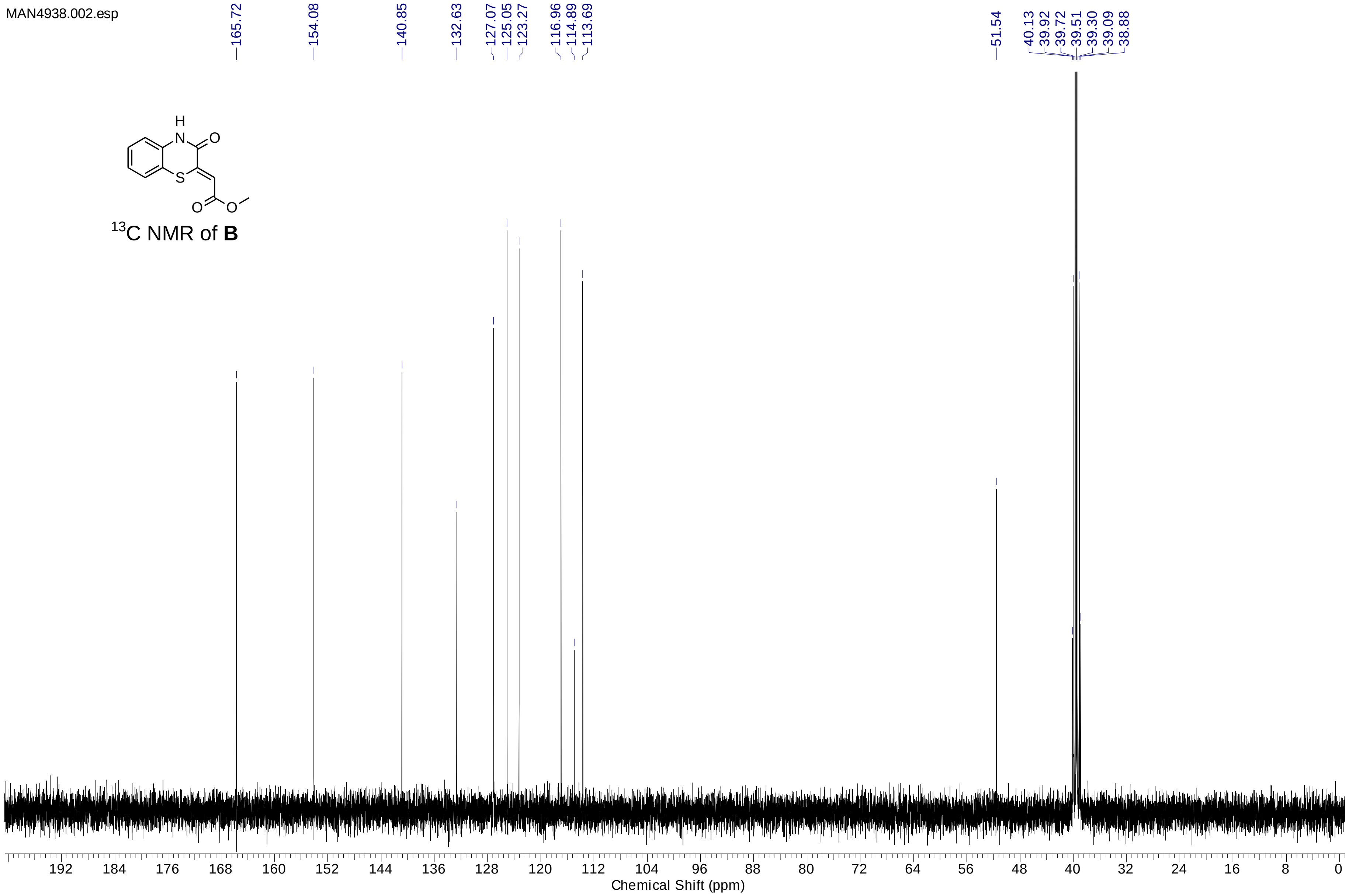
MAN4938.002.esp
165.72
154.08
140.85
132.63
127.07
125.05
123.27
116.96
114.89
113.69
51.54
40.13
39.92
39.72
39.51
39.30
39.09
38.88
H
N
O
S
O
O
192 184 176 168 160 152 144 136 128 120 112 104 96 88 80 72 64 56 48 40 32 24 16 8 0
Chemical Shift (ppm)

^{13}C NMR of **B**

Enaminones fused to the 1,4-benzothiazin-2-one moiety (BTAs) 3a–n. General procedure.

To a cooled (to 0–5 °C) stirring suspension of acylpyruvic acid **2** (5 mmol) and DCC (5 mmol) in acetonitrile (10 mL), *o*-aminothiophenol **1** (5.1 mmol) was added. The mixture was allowed to reach ambient temperature (25 °C) overnight. Then the formed yellow precipitate was filtered off and washed with toluene (50–100 mL; it is necessary to remove precipitated orange crystals of the target compound). The yellow precipitate was disposed of. The collected mother liquor was evaporated to dryness under vacuum. The solid residue was stirred with ethanol (10 mL) for 1 h (it is necessary to remove dicylohexylurea). Then, the orange precipitate was filtered off and recrystallized from toluene (5–10 mL) to give the target BTA **3**.

This procedure can be modified to save acetonitrile. The modified variant affords slightly lower yields of BTAs 3. Modified procedure: To a cooled to 0–5 °C stirring suspension of acylpyruvic acid **2** (5 mmol) and *o*-aminothiophenol **1** (5.1 mmol) in toluene (10 mL), DCC (5 mmol) was added. The mixture was allowed to reach ambient temperature (25 °C) overnight. Then the formed yellow precipitate was filtered off and washed with toluene (50–100 mL; it is necessary to remove precipitated orange crystals of the target compound). The yellow precipitate was disposed of. The collected mother liquor was evaporated to dryness under vacuum. The solid residue was stirred with ethanol (10 mL) for 1 h (it is necessary to remove dicylohexylurea). Then, the orange precipitate was filtered off and recrystallized from toluene (5–10 mL) to give the target BTA **3**.

(3*Z*)-3-(2-Oxo-2-phenylethylidene)-3,4-dihydro-2*H*-1,4-benzothiazin-2-one (3a)
Yield: 691 mg (49%); orange solid; mp 159–160 °C.
^{1}H NMR (400 MHz, DMSO-d_6): δ = 13.84 (s, 1 H), 8.02 (m, 2 H), 7.65-7.39 (m, 6 H), 7.22 (m, 1 H), 6.75 (s, 1 H) ppm.
^{13}C NMR (100 MHz, DMSO-d_6): δ = 190.3, 181.6, 142.8, 138.3, 132.5, 128.8 (2 C), 128.5, 127.2 (2 C), 126.2, 123.7, 118.7, 116.6, 114.4, 88.2 ppm.
IR (mineral oil): 3078, 1659, 1650 cm^{-1}.
MS (ESI+): m/z calcd for $C_{16}H_{11}NO_2S+H^+$: 282.06 [M+H$^+$]; found: 281.98.
Anal. Calcd (%) for $C_{16}H_{11}NO_2S$: C 68.31; H 3.94; N 4.98. Found: C 68.09; H 4.07; N 4.93.
λ_{max} = 444 nm.

(3*Z*)-3-(2-(4-Methylphenyl)-2-oxoethylidene)-3,4-dihydro-2*H*-1,4-benzothiazin-2-one (3b)
Yield: 666 mg (45%); orange solid; mp 176–178 °C.
^{1}H NMR (400 MHz, DMSO-*d*$_6$): δ = 13.81 (s, 1 H), 7.92 (m, 2 H), 7.47 (m, 2 H), 7.42-7.35 (m, 3 H), 7.21 (m, 1 H), 6.73 (s, 1 H), 2.39 (s, 3 H) ppm.
^{13}C NMR (100 MHz, DMSO-*d*$_6$): δ = 190.0, 181.7, 142.9, 142.5, 135.7, 129.7, 129.3 (2 C), 128.5, 127.3 (2 C), 126.2, 123.5, 118.5, 116.4, 88.2, 21.0 ppm.
IR (mineral oil): 3105, 1657 cm^{-1}.
MS (ESI+): m/z calcd for $C_{17}H_{13}NO_2S+H^+$: 296.07 [M+H$^+$]; found: 296.05.
Anal. Calcd (%) for $C_{17}H_{13}NO_2S$: C 69.13; H 4.44; N 4.74. Found: C 68.88; H 4.46; N 4.81.
λ_{max} = 444 nm.

(3*Z*)-3-(2-(4-Methoxyphenyl)-2-oxoethylidene)-3,4-dihydro-2*H*-1,4-benzothiazin-2-one (3c)
Yield: 748 mg (48%); orange solid; mp 166–167 °C.
^{1}H NMR (400 MHz, $CDCl_3$): δ = 13.91 (br.s, 1 H), 8.02 (m, 2 H), 7.30 (m, 1 H), 7.21 (m, 2 H), 7.10 (m, 1 H), 6.98 (m, 2 H), 6.83 (s, 1 H), 3.90 (s, 3 H) ppm.
^{13}C NMR (100 MHz, $CDCl_3$): δ = 190.6, 182.1, 163.2, 142.5, 131.8, 130.5, 129.8 (2 C), 128.3, 126.1, 123.1, 118.2, 117.0, 113.9 (2 C), 89.5, 55.5 ppm.
IR (mineral oil): 3103, 1656 cm^{-1}.
MS (ESI+): m/z calcd for $C_{17}H_{13}NO_3S+H^+$: 312.07 [M+H$^+$]; found: 312.01.
Anal. Calcd (%) for $C_{17}H_{13}NO_3S$: C 65.58; H 4.21; N 4.50. Found: C 65.66; H 4.25; N 4.50.
λ_{max} = 450 nm.

(3*Z*)-3-(2-(4-Ethoxyphenyl)-2-oxoethylidene)-3,4-dihydro-2*H*-1,4-benzothiazin-2-one (3d)
Yield: 782 mg (48%); orange solid; mp 159–161 °C.
^{1}H NMR (400 MHz, DMSO-*d*$_6$): δ = 13.77 (s, 1 H), 7.99 (m, 2 H), 7.46-7.37 (m, 3 H), 7.19 (m, 1 H), 7.05 (m, 2 H), 6.71 (s, 1 H), 4.14 (q, J = 7.0 Hz, 2 H), 1.36 (t, J = 7.0 Hz, 3 H) ppm.
^{13}C NMR (100 MHz, DMSO-*d*$_6$): δ = 189.2, 181.7, 162.1, 142.2, 130.9, 129.9, 129.5 (2 C), 128.5, 126.2, 123.3, 118.4, 116.2, 114.4 (2 C), 88.2, 63.4, 14.4 ppm.

IR (mineral oil): 3164, 1646 cm^{-1}.
MS (ESI+): m/z calcd for $C_{18}H_{15}NO_3S$+H$^+$: 326.09 [M+H$^+$]; found: 326.11.
Anal. Calcd (%) for $C_{18}H_{15}NO_3S$: C 66.44; H 4.65; N 4.30. Found: C 66.67; H 4.68; N 4.30.
λ_{max} = 444 nm.

(3*Z*)-3-(2-(4-Fluorophenyl)-2-oxoethylidene)-3,4-dihydro-2*H*-1,4-benzothiazin-2-one (3e)
Yield: 630 mg (42%); orange solid; mp 175–177 °C.
^{1}H NMR (400 MHz, DMSO-*d*$_6$): δ = 13.79 (s, 1 H), 8.09 (m, 2 H), 7.49 (m, 2 H), 7.43-7.33 (m, 3 H), 7.22 (m, 1 H), 6.72 (s, 1 H) ppm.
^{13}C NMR (100 MHz, DMSO-*d*$_6$): δ = 188.9, 181.5, 165.8, 163.3, 142.8, 134.9, 134.9, 130.1, 130.0, 129.6, 128.5, 126.2, 123.7, 118.7, 116.6, 115.8, 115.6, 88.0 ppm.
^{19}F NMR (377 MHz, DMSO-*d*$_6$): δ = 107.33 ppm.
IR (mineral oil): 3176, 1677, 1656 cm^{-1}.
MS (ESI+): m/z calcd for $C_{16}H_{10}FNO_2S$+H$^+$: 300.05 [M+H$^+$]; found: 300.08.
Anal. Calcd (%) for $C_{16}H_{10}FNO_2S$: C 64.20; H 3.37; N 4.68. Found: C 64.39; H 3.40; N 4.66.
λ_{max} = 444 nm.

(3*Z*)-3-(2-(4-Chlorophenyl)-2-oxoethylidene)-3,4-dihydro-2*H*-1,4-benzothiazin-2-one (3f)
Yield: 711 mg (45%); orange solid; mp 190–191 °C.
^{1}H NMR (400 MHz, DMSO-*d*$_6$): δ = 13.82 (s, 1 H), 8.02 (m, 2 H), 7.60 (m, 2 H), 7.49 (m, 2 H), 7.42 (m, 1 H), 7.23 (m, 1 H), 6.72 (s, 1 H) ppm.
^{13}C NMR (100 MHz, DMSO-*d*$_6$): δ = 188.9, 181.4, 143.0, 137.3, 136.9, 129.5, 129.0 (2 C), 128.8 (2 C), 128.5, 126.2, 123.8, 118.8, 116.8, 88.0 ppm.
IR (mineral oil): 3113, 1675 cm^{-1}.
MS (ESI+): m/z calcd for $C_{16}H_{10}ClNO_2S$ +H$^+$: 316.02 [M+H$^+$]; found: 316.00.
Anal. Calcd (%) for $C_{16}H_{10}ClNO_2S$: C 60.86; H 3.19; N 4.44. Found: C 60.57; H 3.21; N 4.48.
λ_{max} = 450 nm.

(3*Z*)-3-(2-(4-Bromophenyl)-2-oxoethylidene)-3,4-dihydro-2*H*-1,4-benzothiazin-2-one (3g)
Yield: 864 mg (48%); orange solid; mp 208–209 °C.
^{1}H NMR (400 MHz, DMSO-d_6): δ = 13.83 (s, 1 H), 7.95 (m, 2 H), 7.74 (m, 2 H), 7.50 (m, 2 H), 7.42 (m, 1 H), 7.23 (m, 1 H), 6.72 (s, 1 H) ppm.
^{13}C NMR (100 MHz, DMSO-d_6): δ = 189.0, 181.5, 143.0, 137.3, 131.8 (2 C), 129.5, 129.2 (2 C), 128.5, 126.4, 126.3, 123.8, 118.8, 116.8, 88.0 ppm.
IR (mineral oil): 3058, 1659 cm^{-1}.
MS (ESI+): m/z calcd for $C_{16}H_{10}BrNO_2S+H^+$: 359.97, 361.97 [M+H$^+$]; found: 359.99, 361.99.
Anal. Calcd (%) for $C_{16}H_{10}BrNO_2S$: C 53.35; H 2.80; N 3.89. Found: C 53.18; H 2.87; N 4.00.
λ_{max} = 444 nm.

(3*Z*)-3-(2-(4-Nitrophenyl)-2-oxoethylidene)-3,4-dihydro-2*H*-1,4-benzothiazin-2-one (3h)
was not isolated since it was formed only in trace amounts and detected by UPLC-UV-MS.
MS (ESI+): m/z calcd for $C_{16}H_{10}N_2O_4S+H^+$: 327.04 [M+H$^+$]; found: 326.89.
λ_{max} = 454 nm.

(3*Z*)-3-(2-(Naphthalen-1-yl)-2-oxoethylidene)-3,4-dihydro-2*H*-1,4-benzothiazin-2-one (3i)
Yield: 846 mg (51%); orange solid; mp 143–144 °C.
^{1}H NMR (400 MHz, DMSO-d_6): δ = 13.71 (s, 1 H), 8.50 (m, 1 H), 8.10 (m, 1 H), 8.01 (m, 1 H), 7.88 (m, 1 H), 7.64-7.40 (m, 6 H), 7.23 (m, 1 H), 6.50 (s, 1 H) ppm.
^{13}C NMR (100 MHz, DMSO-d_6): δ = 194.5, 181.5, 142.2, 137.5, 133.4, 131.5, 129.6, 129.3, 128.5, 128.4, 127.2, 126.8, 126.3, 126.3, 125.2, 125.0, 123.7, 118.7, 116.6, 92.5 ppm.
IR (mineral oil): 3114, 1665 cm^{-1}.
MS (ESI+): m/z calcd for $C_{20}H_{13}NO_2S+H^+$: 332.07 [M+H$^+$]; found: 332.04.
Anal. Calcd (%) for $C_{20}H_{13}NO_2S$: C 72.49; H 3.95; N 4.23. Found: C 72.83; H 4.04; N 4.12.
λ_{max} = 443 nm.

(3*Z*)-3-(2-(Naphthalen-2-yl)-2-oxoethylidene)-3,4-dihydro-2*H*-1,4-benzothiazin-2-one (3j)
Yield: 813 mg (49%); orange solid; mp 201–203 °C.
^{1}H NMR (400 MHz, DMSO-d_6): δ = 13.93 (s, 1 H), 8.70 (s, 1 H), 8.20 (m, 1 H), 8.10-7.98 (m, 3 H), 7.64 (m, 2 H), 7.50 (m, 2 H), 7.43 (m, 1 H), 7.23 (m, 1 H), 6.95 (s, 1 H) ppm.
^{13}C NMR (100 MHz, DMSO-d_6): δ = 190.2, 181.7, 142.8, 135.6, 134.7, 132.3, 129.7, 129.5, 128.5, 128.4, 128.4, 128.3, 127.5, 126.7, 126.3, 123.7, 123.3, 118.7, 116.6, 88.5 ppm.
IR (mineral oil): 3130, 1660 cm^{-1}.
MS (ESI+): m/z calcd for $C_{20}H_{13}NO_2S+H^+$: 332.07 [M+H$^+$]; found: 332.05.
Anal. Calcd (%) for $C_{20}H_{13}NO_2S$: C 72.49; H 3.95; N 4.23. Found: C 72.21; H 4.03; N 4.22.
λ_{max} = 440 nm.

(3*Z*)-3-(2-Oxo-2-(thiophen-2-yl)ethylidene)-3,4-dihydro-2*H*-1,4-benzothiazin-2-one (3k)
Yield: 806 mg (56%); orange solid; mp 184–185 °C.
^{1}H NMR (400 MHz, DMSO-d_6): δ = 13.45 (s, 1 H), 7.98 (m, 2 H), 7.47-7.37 (m, 3 H), 7.26-7.17 (m, 2 H), 6.61 (s, 1 H) ppm.
^{13}C NMR (100 MHz, DMSO-d_6): δ = 183.4, 181.4, 145.4, 142.3, 134.1, 131.0, 129.7, 128.8, 128.5, 126.2, 123.5, 118.5, 116.3, 88.6 ppm.
IR (mineral oil): 3181, 1654 cm^{-1}.
MS (ESI+): m/z calcd for $C_{14}H_9NO_2S_2+H^+$: 288.02 [M+H$^+$]; found: 288.04.
Anal. Calcd (%) for $C_{14}H_9NO_2S_2$: C 58.52; H 3.16; N 4.87. Found: C 58.41; H 3.18; N 4.93.
λ_{max} = 452 nm.

(3*Z*)-3-(2-(Furan-2-yl)-2-oxoethylidene)-3,4-dihydro-2*H*-1,4-benzothiazin-2-one (3l)
Yield: 789 mg (58%); orange solid; mp 174–176 °C.
^{1}H NMR (400 MHz, DMSO-d_6): δ = 13.44 (s, 1 H), 7.99 (m, 1 H), 7.47-7.37 (m, 4 H), 7.20 (m, 1 H), 6.73 (m, 1 H), 6.55 (s, 1 H) ppm.
^{13}C NMR (100 MHz, DMSO-d_6): δ = 181.4, 179.1, 152.9, 147.2, 142.5, 129.7, 128.5, 126.2, 123.5, 118.4, 116.3, 116.2, 112.8, 88.5 ppm.
IR (mineral oil): 3107, 1658, 1645 cm^{-1}.
MS (ESI+): m/z calcd for $C_{14}H_9NO_3S+H^+$: 272.04 [M+H$^+$]; found: 272.02.

Anal. Calcd (%) for $C_{14}H_9NO_3S$: C 61.98; H 3.34; N 5.16. Found: C 61.69; H 3.33; N 5.31.
λ_{max} = 450 nm.

(3*Z*)-3-(3,3-Dimethyl-2-oxobutylidene)-3,4-dihydro-2*H*-1,4-benzothiazin-2-one (3m)
Yield: 535 mg (41%); orange solid; mp 131–132 °C.
^{1}H NMR (400 MHz, DMSO-d_6): δ = 13.27 (s, 1 H), 7.38 (m, 3 H), 7.16 (m, 1 H), 6.20 (s, 1 H), 1.18 (s, 9 H) ppm.
^{13}C NMR (100 MHz, DMSO-d_6): δ = 207.3, 181.8, 141.9, 129.9, 128.4, 126.1, 123.2, 118.2, 115.9, 87.5, 42.7, 26.8 (3 C) ppm.
IR (mineral oil): 3148, 1654 cm^{-1}.
MS (ESI+): m/z calcd for $C_{14}H_{15}NO_2S+H^+$: 261.08 [$M+H^+$]; found: 261.01.
Anal. Calcd (%) for $C_{14}H_{15}NO_2S$: C 64.34; H 5.79; N 5.36. Found: C 64.36; H 5.70; N 5.33.
λ_{max} = 425 nm.

(3*Z*)-6-Chloro-3-(2-oxo-2-phenylethylidene)-3,4-dihydro-2*H*-1,4-benzothiazin-2-one (3n)
Yield: 253 mg (16%); orange solid; mp 156–158 °C.
^{1}H NMR (400 MHz, DMSO-d_6): δ = 13.57 (s, 1 H), 8.01 (m, 2 H), 7.71 (m, 1 H), 7.64 (m, 1 H), 7.56 (m, 2 H), 7.49 (m, 1 H), 7.24 (m, 1 H), 6.77 (s, 1 H) ppm.
^{13}C NMR (100 MHz, DMSO-d_6): δ = 190.5, 181.2, 142.1, 138.1, 132.7, 131.0, 128.8 (2 C), 128.5, 127.7, 127.2 (2 C), 123.3, 118.2, 115.4, 89.2 ppm.
IR (mineral oil): 3179, 1671, 1661 cm^{-1}.
MS (ESI+): m/z calcd for $C_{16}H_{10}ClNO_2S$ $+H^+$: 316.02 [$M+H^+$]; found: 316.04.
Anal. Calcd (%) for $C_{16}H_{10}ClNO_2S$: C 60.86; H 3.19; N 4.44. Found: C 60.93; H 3.23; N 4.31.
λ_{max} = 439 nm.

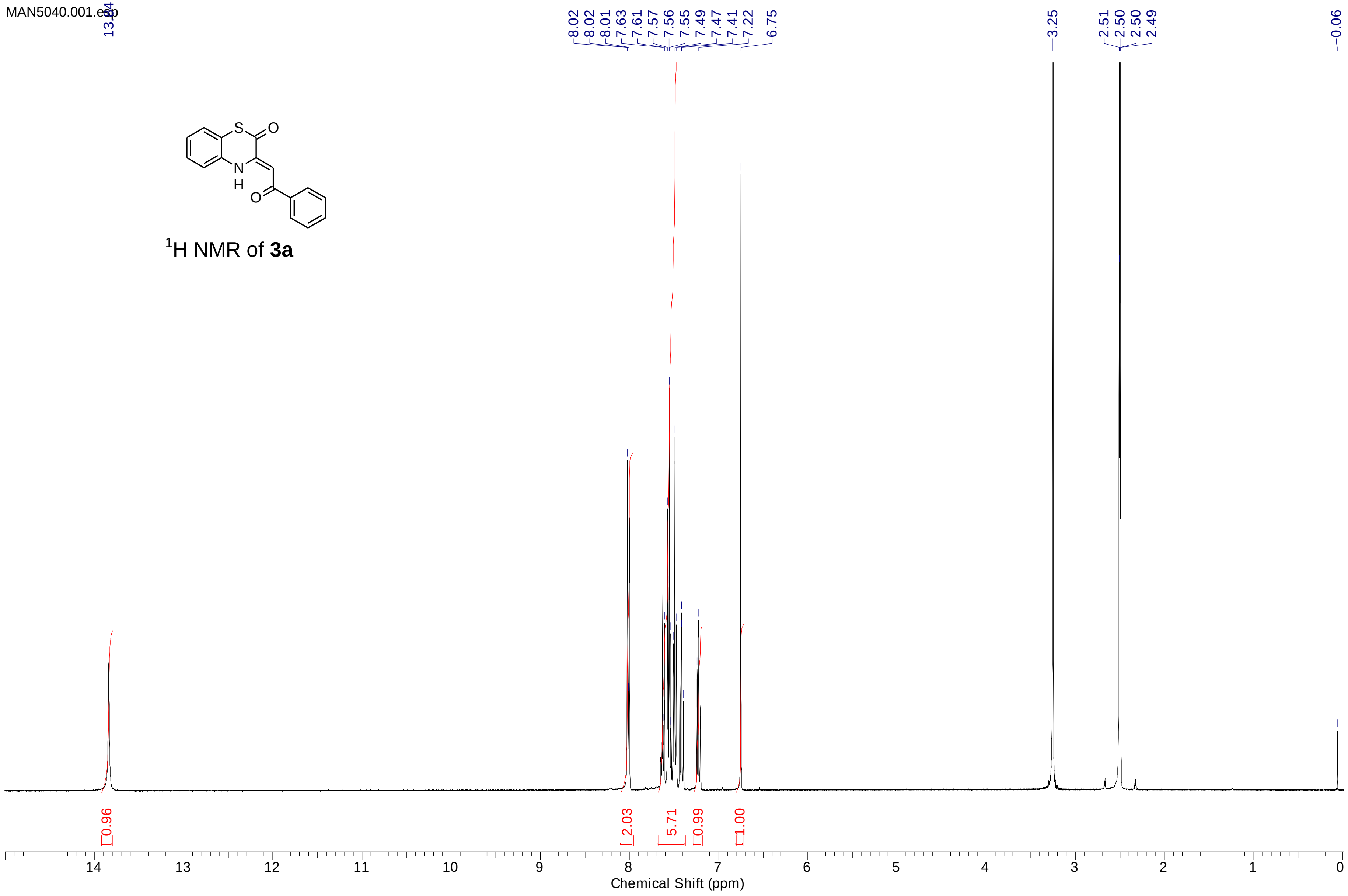
MAN5040.001.esp
13.84
8.02
8.02
8.01
7.63
7.61
7.57
7.56
7.55
7.49
7.47
7.41
7.22
6.75
3.25
2.51
2.50
2.50
2.49
0.06
S
O
N
H
O
1H NMR of 3a
0.96
2.03
5.71
0.99
1.00
14
13
12
11
10
9
8
7
6
5
4
3
2
1
0
Chemical Shift (ppm)

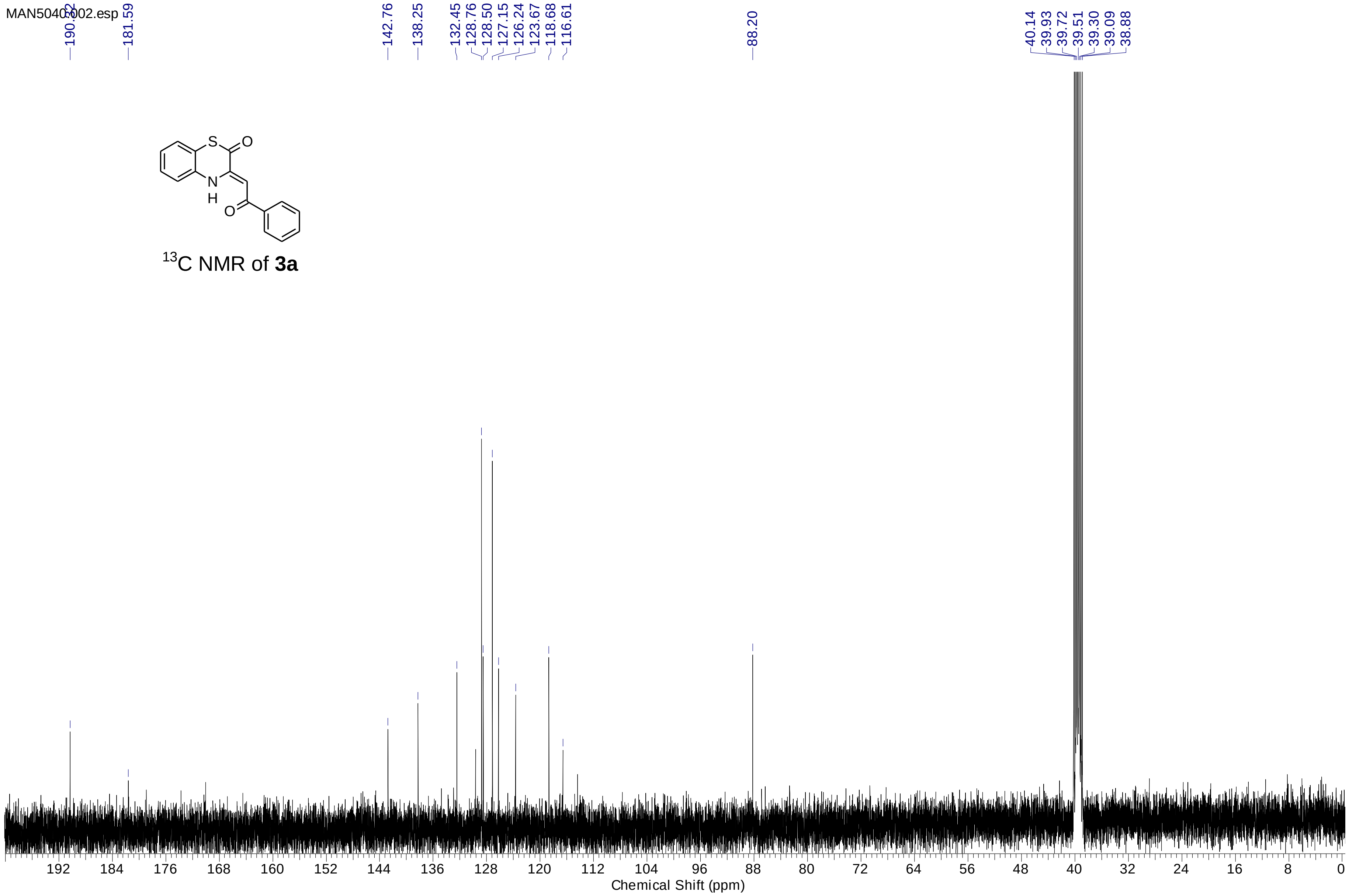
MAN5040.002.esp
190.32
181.59
142.76
138.25
132.45
128.76
128.50
127.15
126.24
123.67
118.68
116.61
88.20
40.14
39.93
39.72
39.51
39.30
39.09
38.88
S
O
N
H
O
192
184
176
168
160
152
144
136
128
120
112
104
96
88
80
72
64
56
48
40
32
24
16
8
0
Chemical Shift (ppm)

^{13}C NMR of **3a**

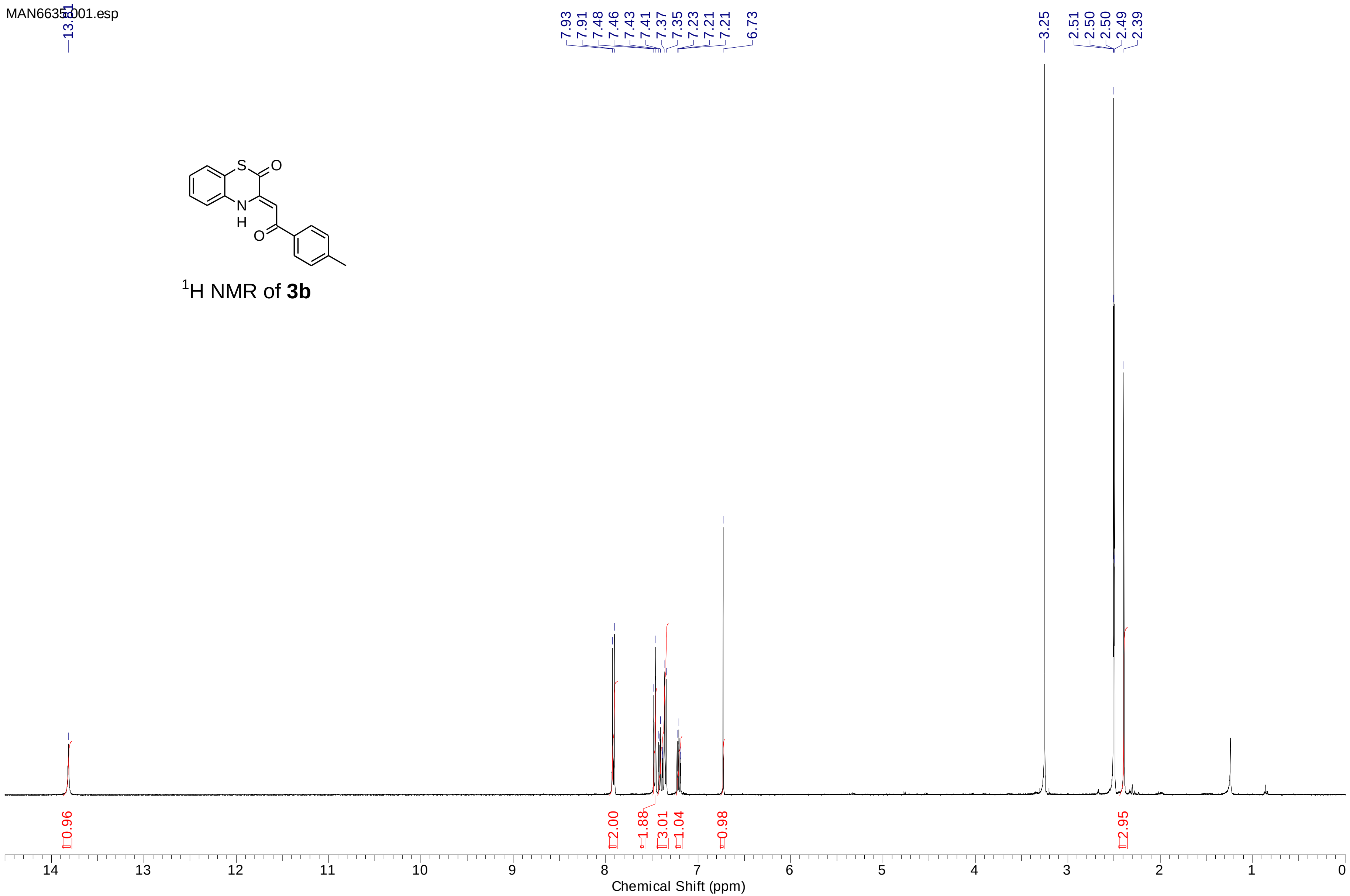
MAN6635001.esp
13.81
7.93
7.91
7.48
7.46
7.43
7.41
7.37
7.35
7.23
7.21
7.21
6.73
3.25
2.51
2.50
2.50
2.49
2.39
S
O
N
H
O
^{1}H NMR of **3b**
0.96
2.00
1.88
3.01
1.04
0.98
2.95
14
13
12
11
10
9
8
7
6
5
4
3
2
1
0
Chemical Shift (ppm)

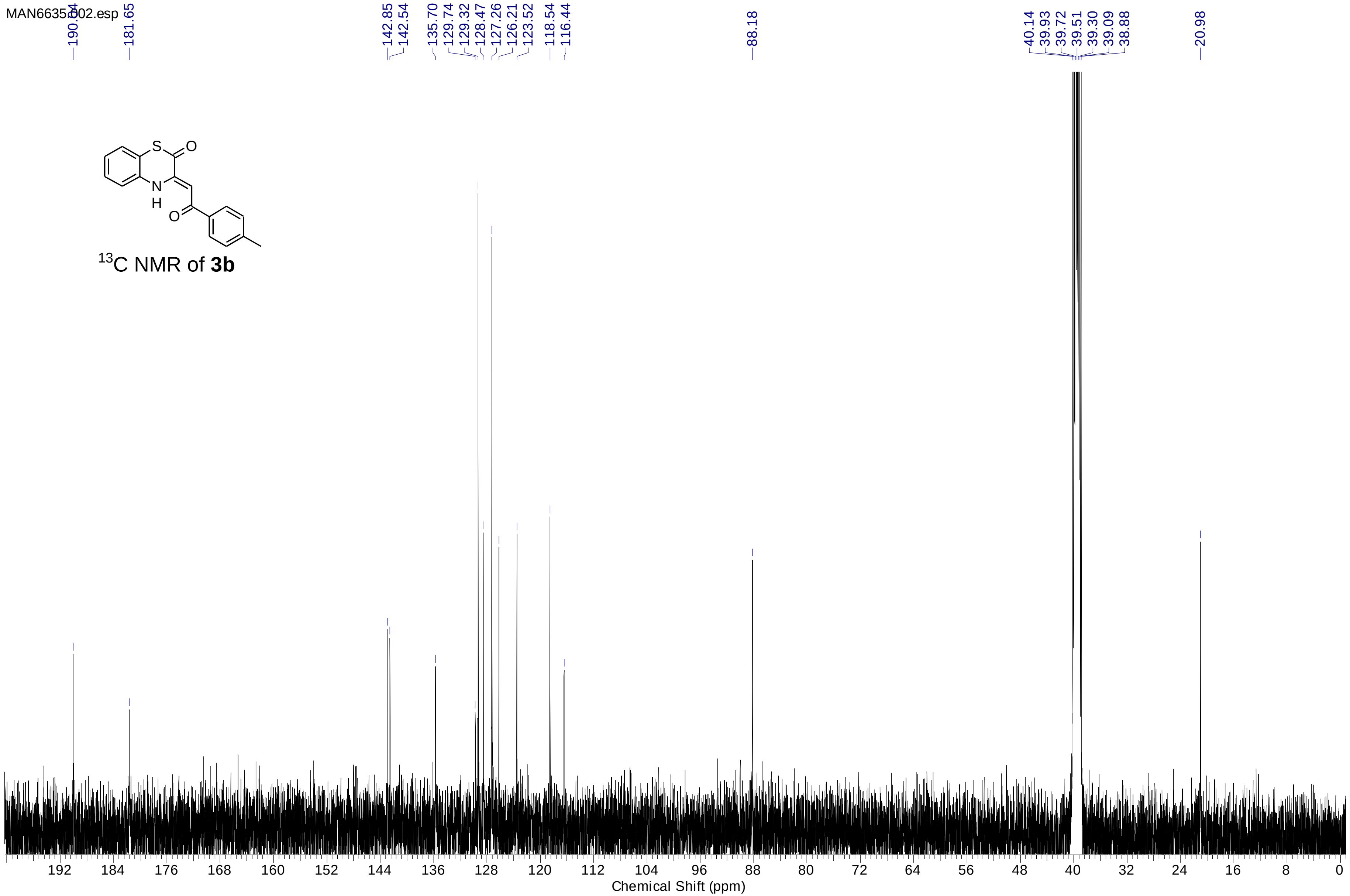
MAN6635.002.esp
190.04
181.65
142.85
142.54
135.70
129.74
129.32
128.47
127.26
126.21
123.52
118.54
116.44
88.18
40.14
39.93
39.72
39.51
39.30
39.09
38.88
20.98
S
O
N
H
O
192 184 176 168 160 152 144 136 128 120 112 104 96 88 80 72 64 56 48 40 32 24 16 8 0
Chemical Shift (ppm)

^{13}C NMR of **3b**

MAN6120.001.esp

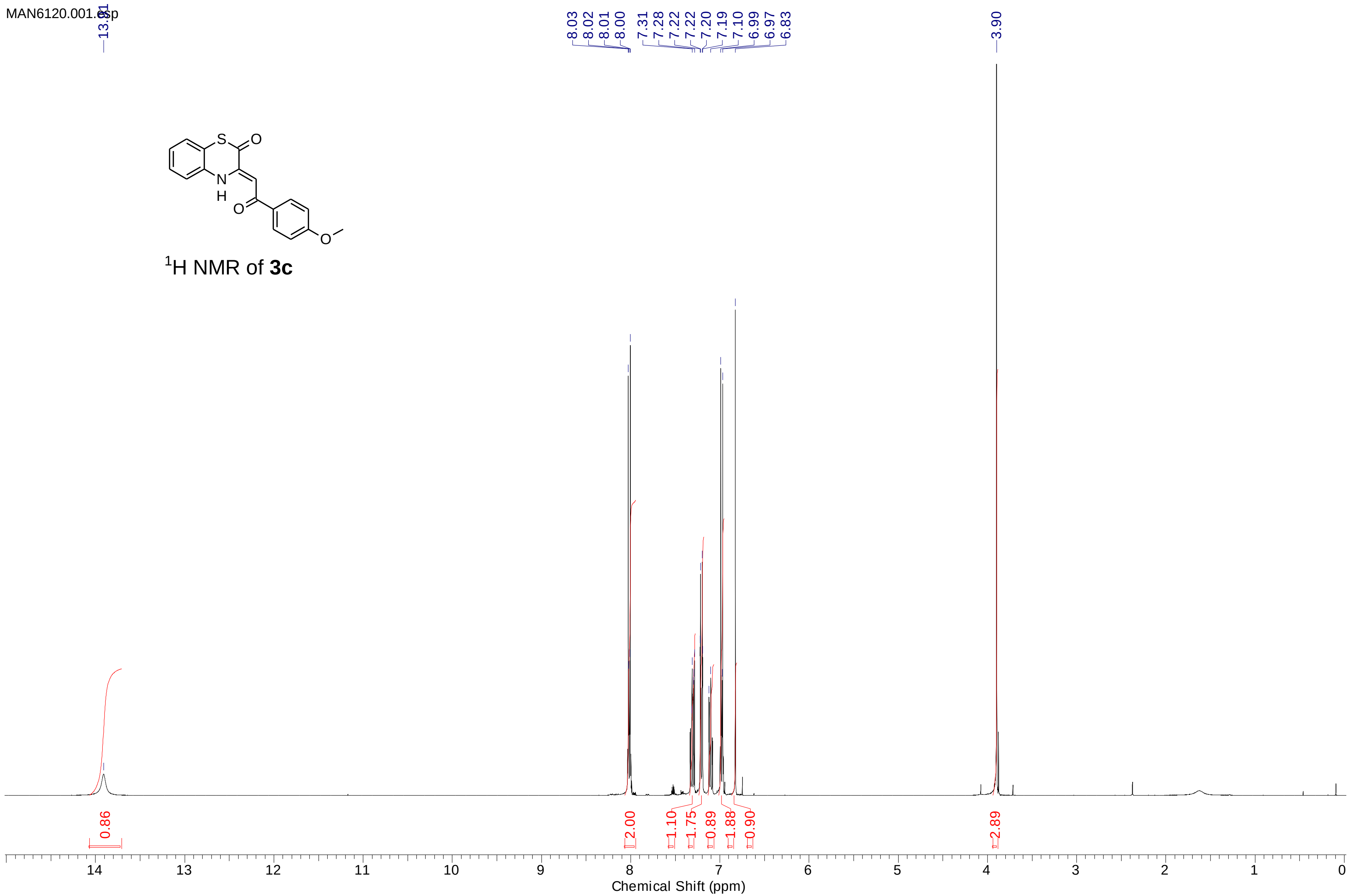

^{1}H NMR of **3c**

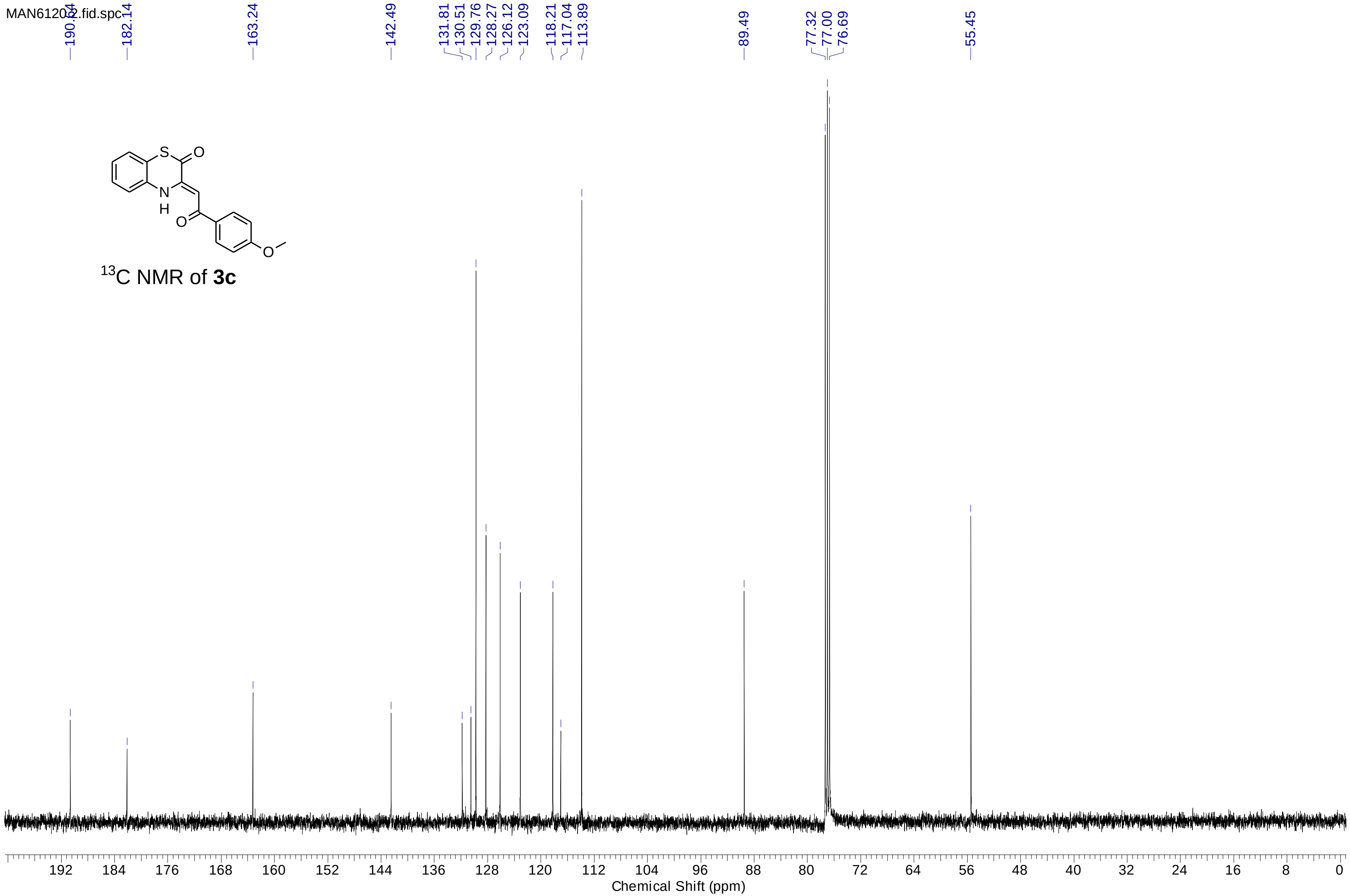
190.64
182.14
163.24
142.49
131.81
130.51
129.76
128.27
126.12
123.09
118.21
117.04
113.89
89.49
77.32
77.00
76.69
55.45
S
O
N
H
O
O
192 184 176 168 160 152 144 136 128 120 112 104 96 88 80 72 64 56 48 40 32 24 16 8 0
Chemical Shift (ppm)

^{13}C NMR of **3c**

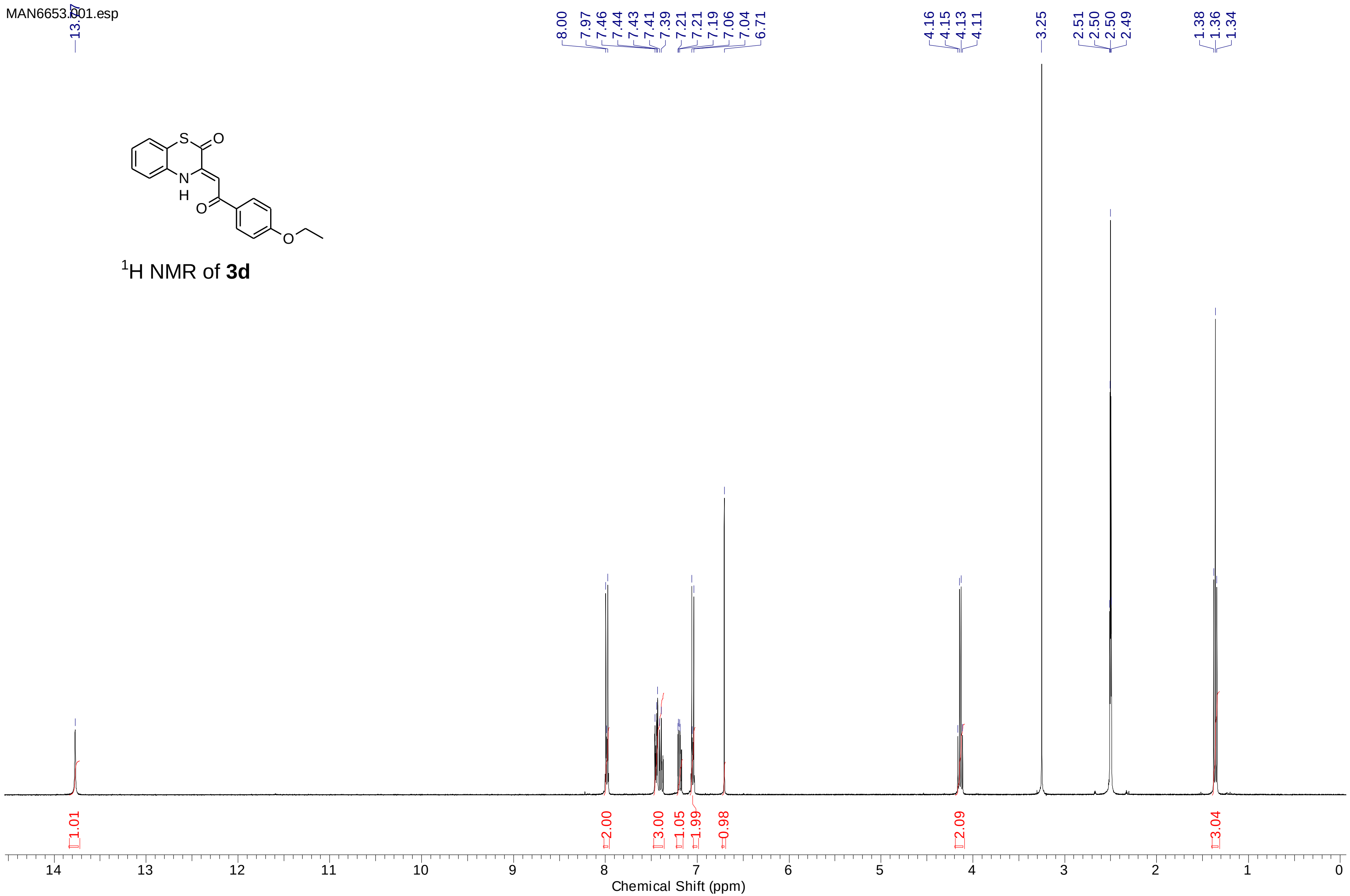
MAN6653.001.esp
13.77
8.00
7.97
7.46
7.44
7.43
7.41
7.39
7.21
7.21
7.19
7.06
7.04
6.71
4.16
4.15
4.13
4.11
3.25
2.51
2.50
2.50
2.49
1.38
1.36
1.34
1.01
2.00
3.00
1.05
1.99
0.98
2.09
3.04
Chemical Shift (ppm)

^{1}H NMR of **3d**

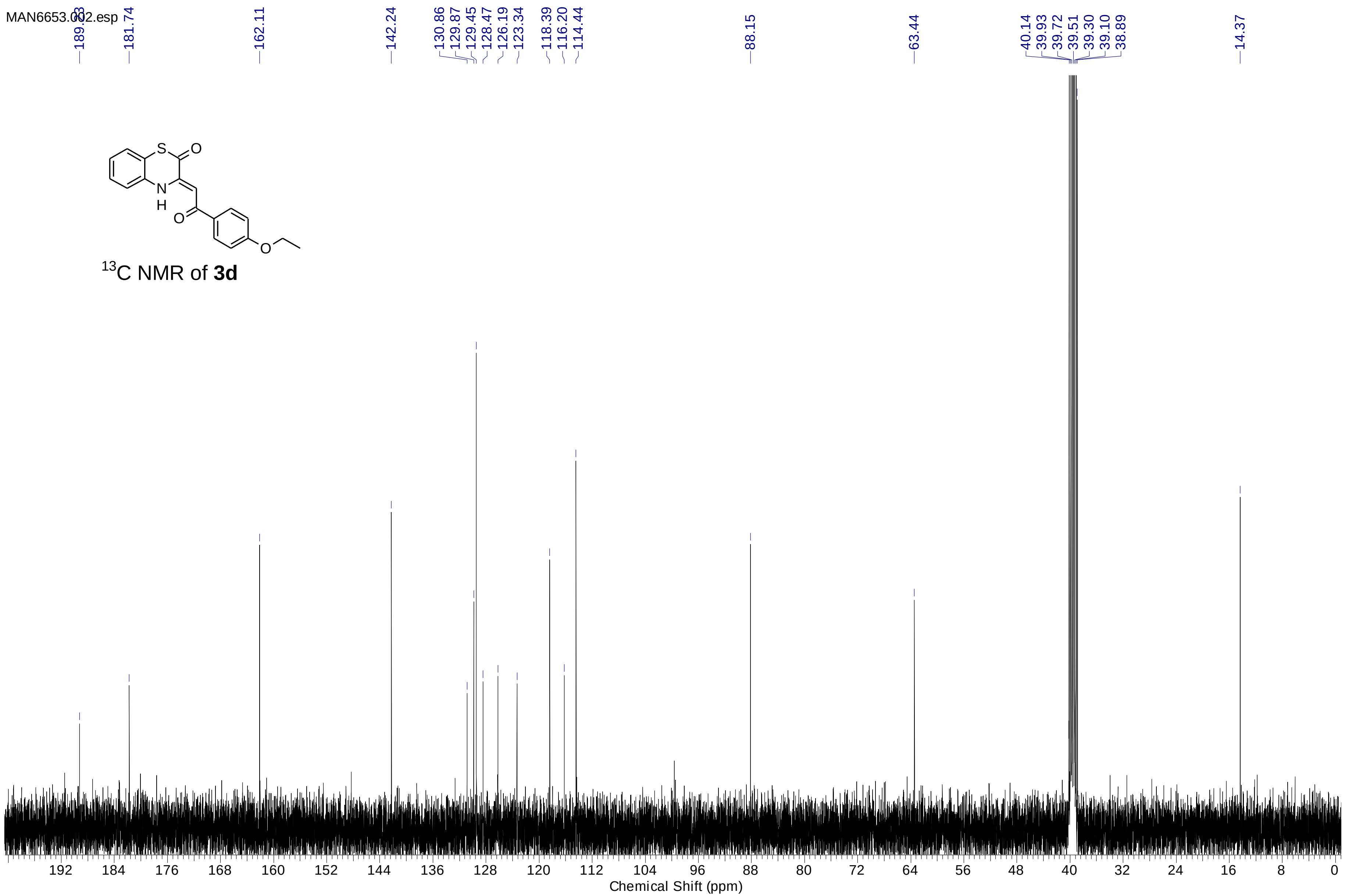

MAN6653.002.esp
189.23
181.74
162.11
142.24
130.86
129.87
129.45
128.47
126.19
123.34
118.39
116.20
114.44
88.15
63.44
40.14
39.93
39.72
39.51
39.30
39.10
38.89
14.37
S
O
N
H
O
O
^{13}C NMR of **3d**
192 184 176 168 160 152 144 136 128 120 112 104 96 88 80 72 64 56 48 40 32 24 16 8 0
Chemical Shift (ppm)

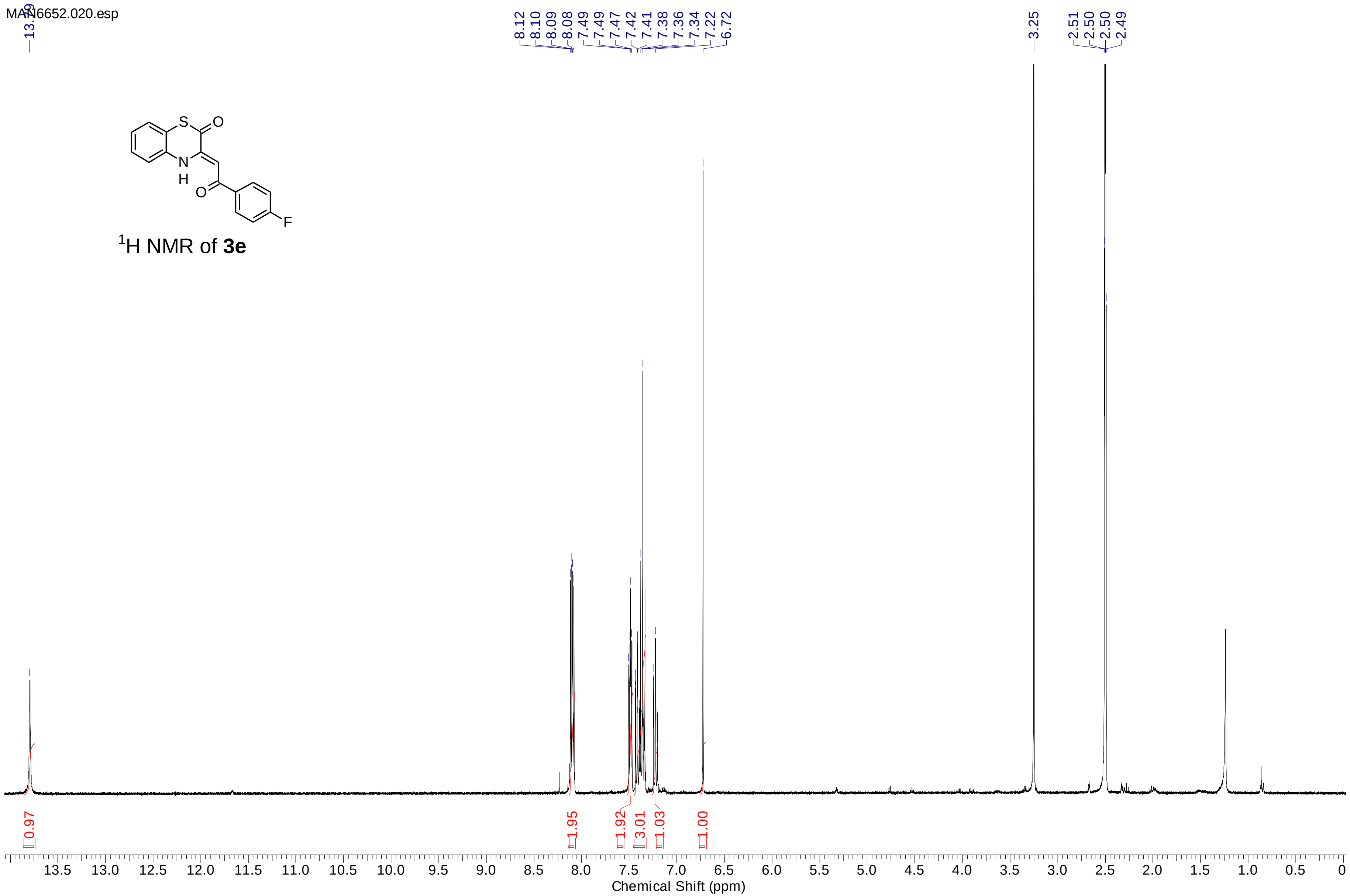
MAN6652.020.esp
13.79
8.12
8.10
8.09
8.08
7.49
7.49
7.47
7.42
7.41
7.38
7.36
7.34
7.22
6.72
3.25
2.51
2.50
2.50
2.49
S
O
N
H
O
F
0.97
1.95
1.92
3.01
1.03
1.00
13.5
13.0
12.5
12.0
11.5
11.0
10.5
10.0
9.5
9.0
8.5
8.0
7.5
7.0
6.5
6.0
5.5
5.0
4.5
4.0
3.5
3.0
2.5
2.0
1.5
1.0
0.5
0
Chemical Shift (ppm)

^{1}H NMR of **3e**

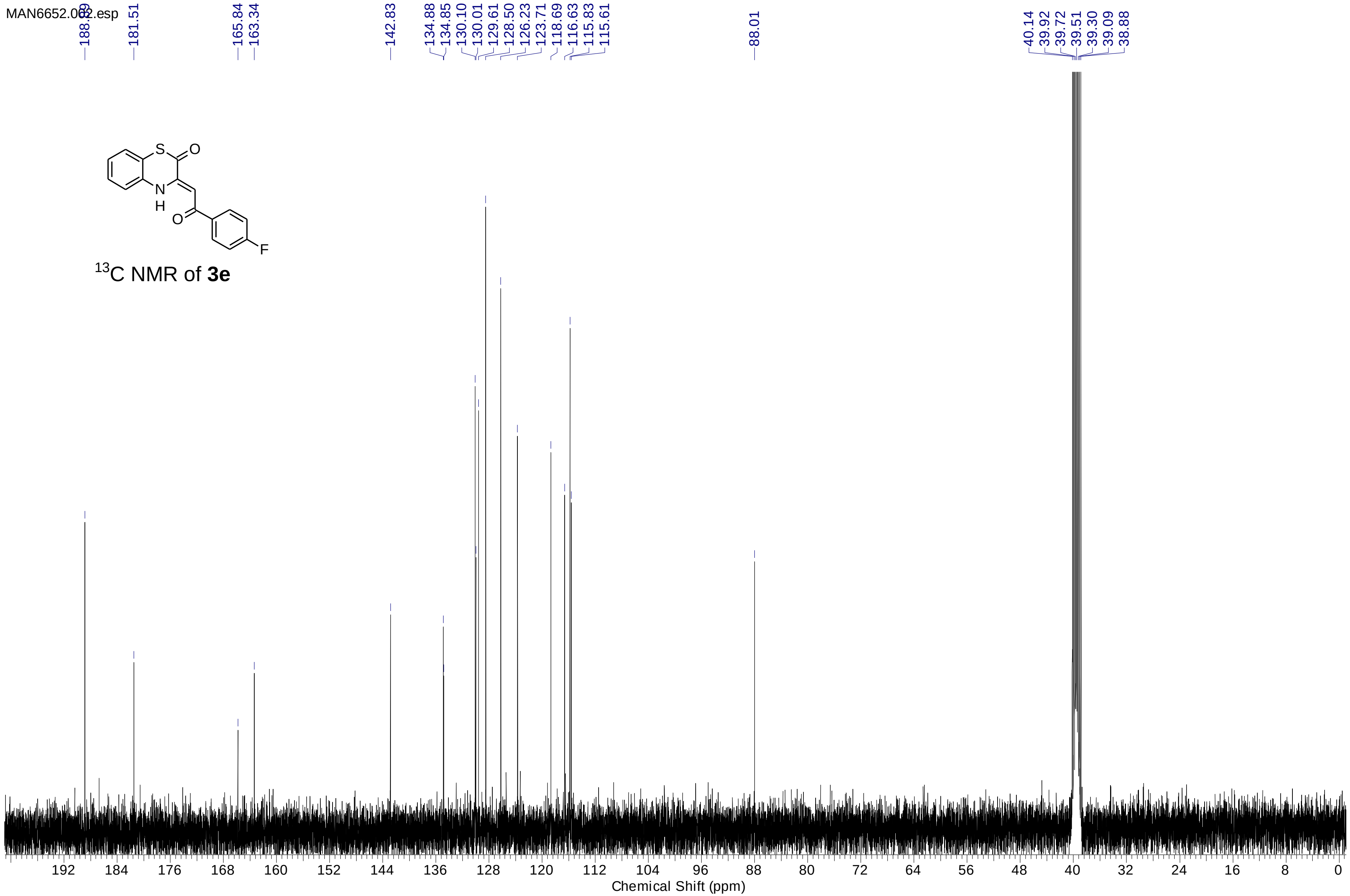
181.51
165.84
163.34
142.83
134.88
134.85
130.10
130.01
129.61
128.50
126.23
123.71
118.69
116.63
115.83
115.61
88.01
40.14
39.92
39.72
39.51
39.30
39.09
38.88
S
O
N
H
O
F
^{13}C NMR of **3e**
192 184 176 168 160 152 144 136 128 120 112 104 96 88 80 72 64 56 48 40 32 24 16 8 0
Chemical Shift (ppm)

MAN6652.004.esp

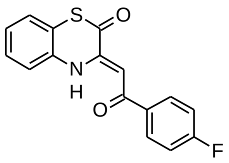

^{19}F NMR of **3e**

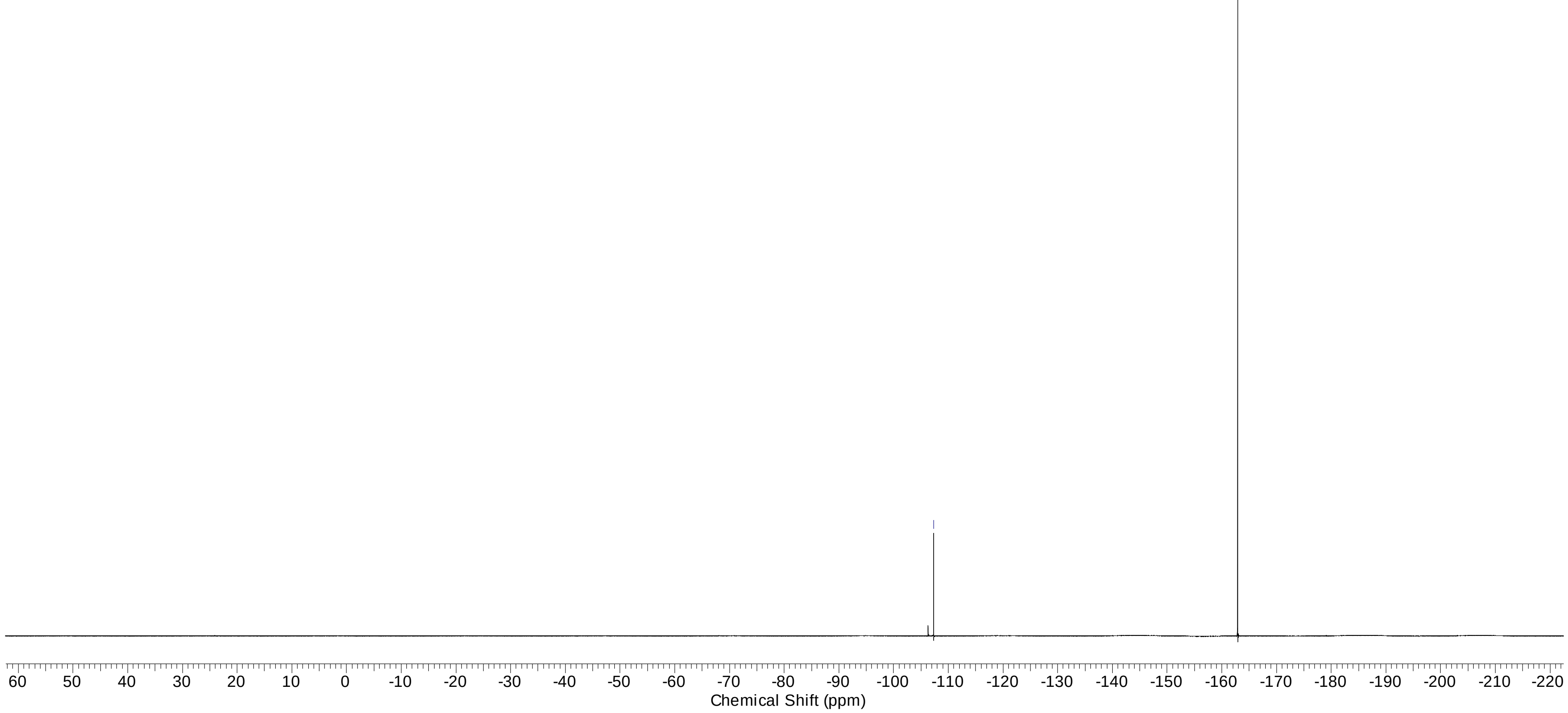

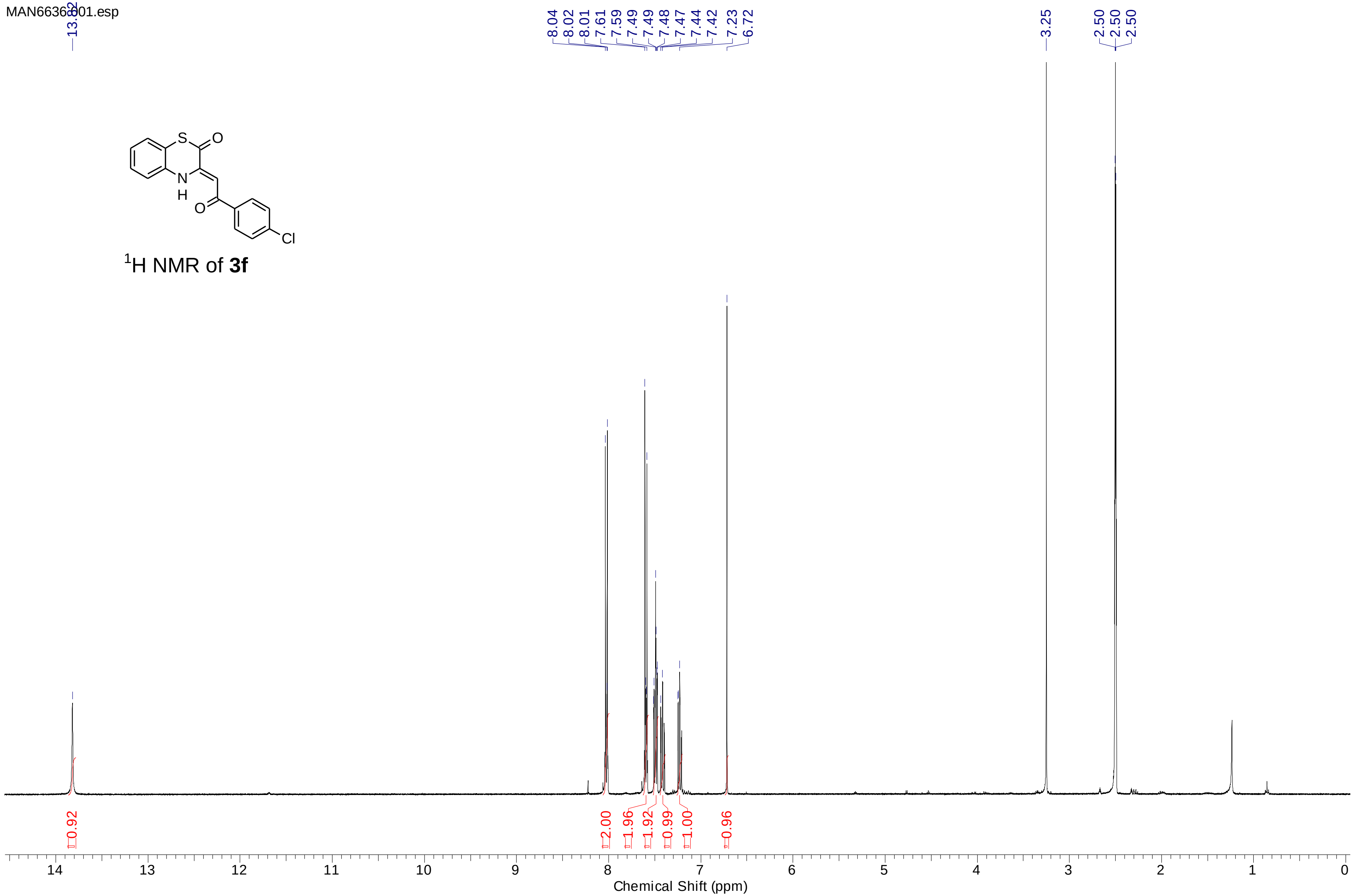
MAN6636s01.esp
13.82
8.04
8.02
8.01
7.61
7.59
7.49
7.49
7.48
7.47
7.44
7.42
7.23
6.72
3.25
2.50
2.50
2.50
0.92
2.00
1.96
1.92
0.99
1.00
0.96
14
13
12
11
10
9
8
7
6
5
4
3
2
1
0
Chemical Shift (ppm)
S
O
N
H
O
Cl
^{1}H NMR of 3f

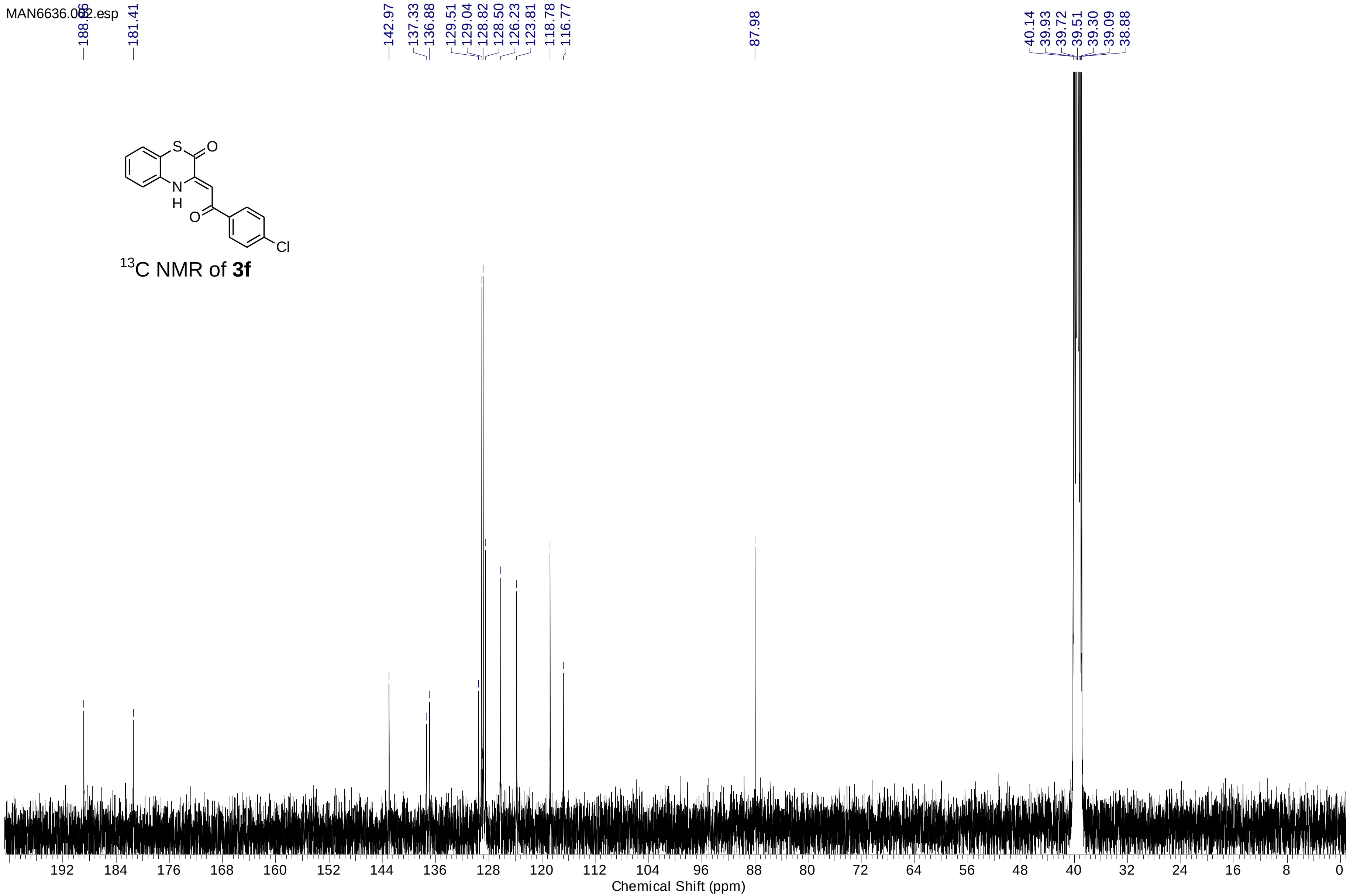
188.86
181.41
142.97
137.33
136.88
129.51
129.04
128.82
128.50
126.23
123.81
118.78
116.77
87.98
40.14
39.93
39.72
39.51
39.30
39.09
38.88
S
O
N
H
O
Cl
^{13}C NMR of **3f**
192
184
176
168
160
152
144
136
128
120
112
104
96
88
80
72
64
56
48
40
32
24
16
8
0
Chemical Shift (ppm)

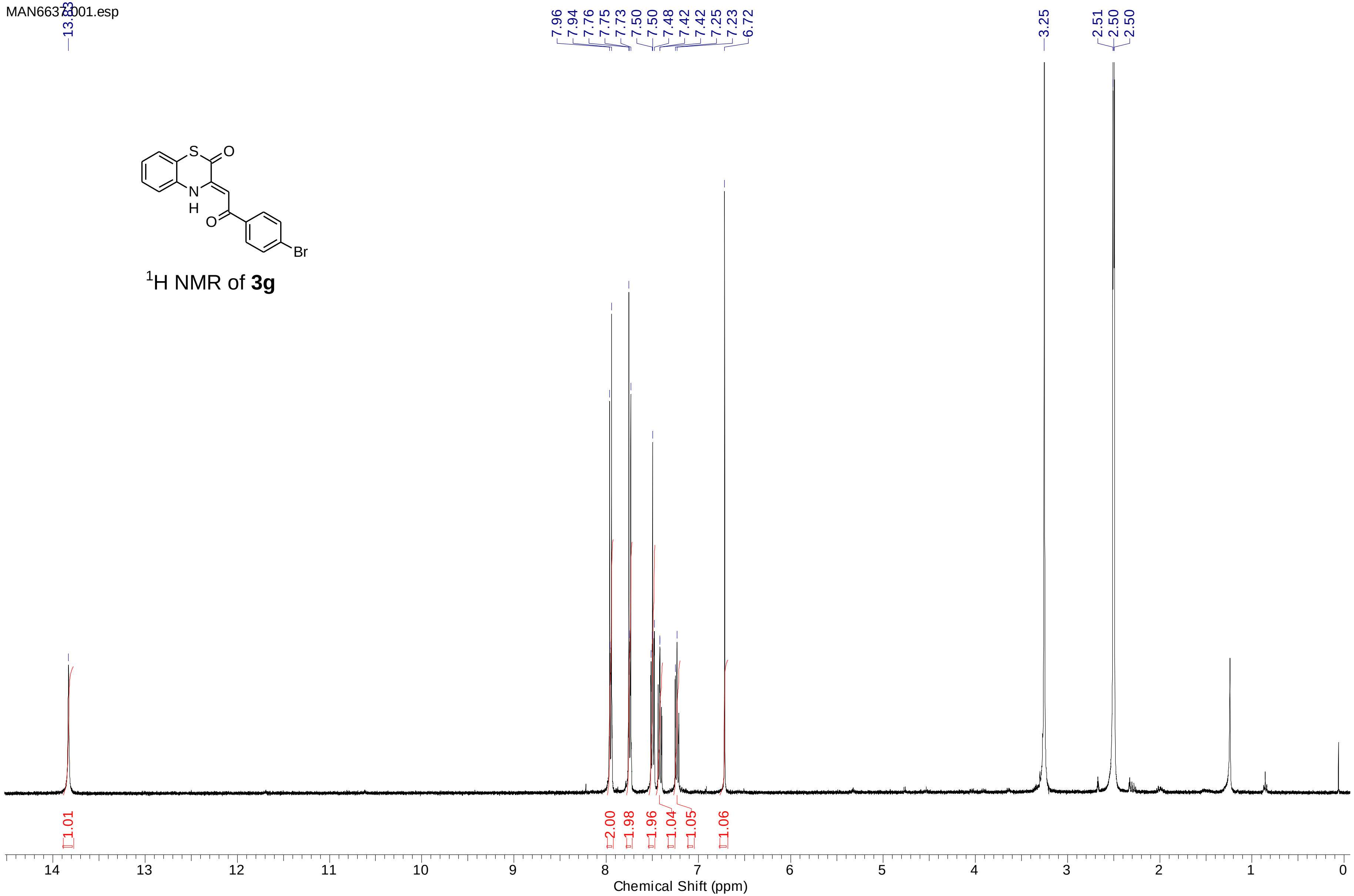
MAN6637.001.esp
13.83
7.96
7.94
7.76
7.75
7.73
7.50
7.50
7.48
7.42
7.42
7.25
7.23
6.72
3.25
2.51
2.50
2.50
S
O
N
H
O
Br
^{1}H NMR of **3g**
1.01
2.00
1.98
1.96
1.04
1.05
1.06
14
13
12
11
10
9
8
7
6
5
4
3
2
1
0
Chemical Shift (ppm)

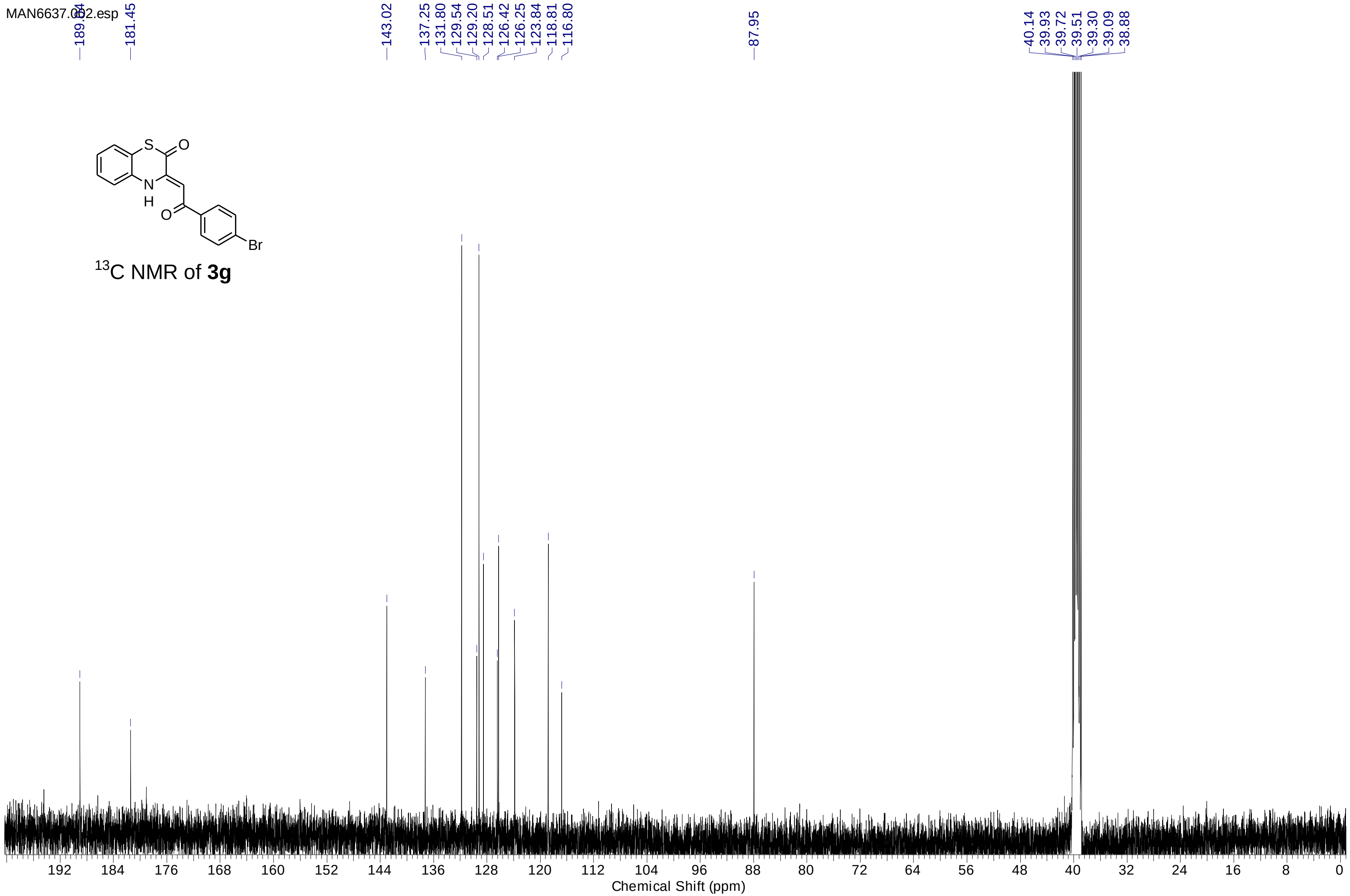
MAN6637.002.esp
189.04
181.45
143.02
137.25
131.80
129.54
129.20
128.51
126.42
126.25
123.84
118.81
116.80
87.95
40.14
39.93
39.72
39.51
39.30
39.09
38.88
S
O
N
H
O
Br
192
184
176
168
160
152
144
136
128
120
112
104
96
88
80
72
64
56
48
40
32
24
16
8
0
Chemical Shift (ppm)

^{13}C NMR of **3g**

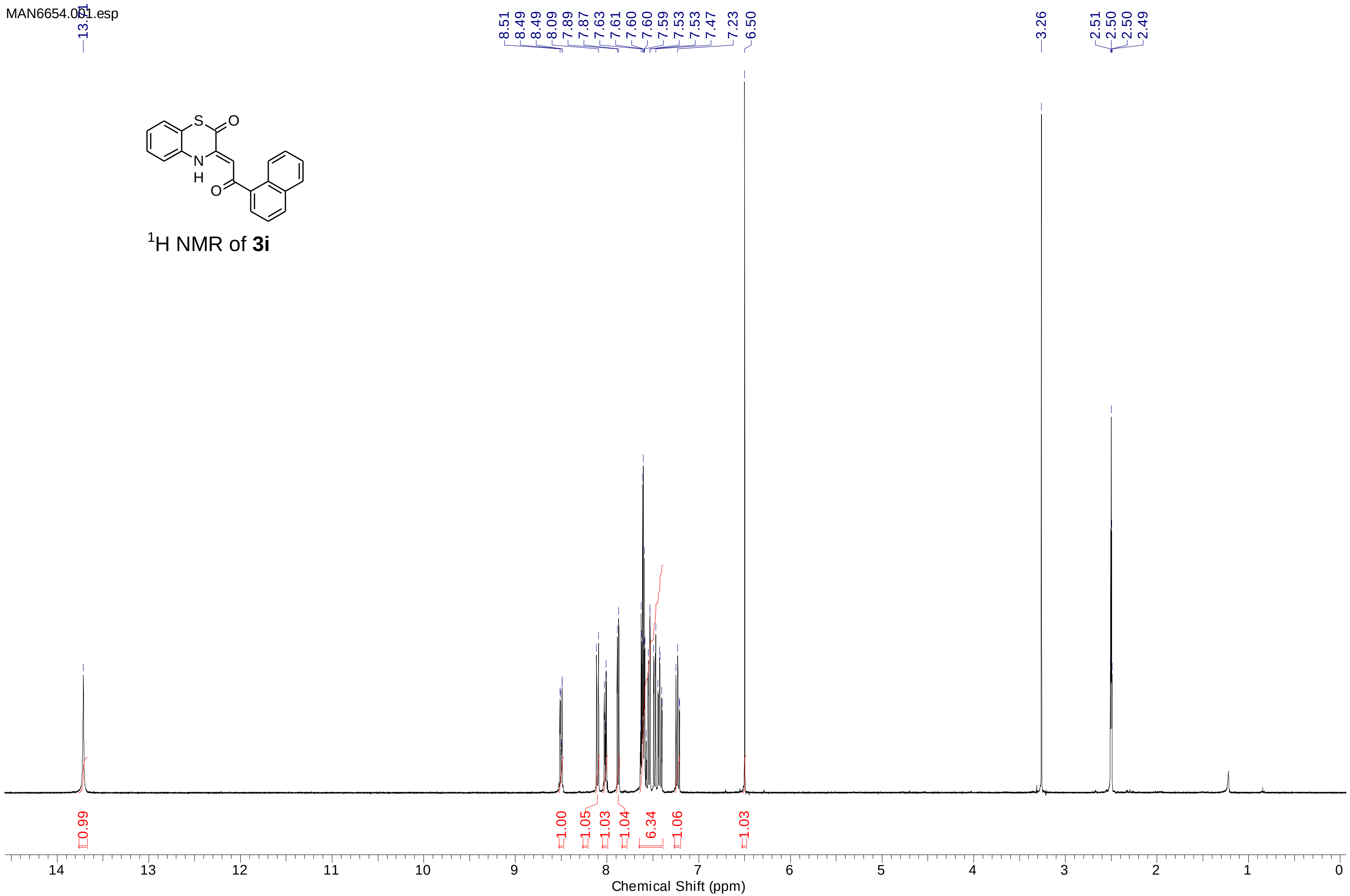
MAN6654.001.esp
13.71
8.51
8.49
8.49
8.09
7.89
7.87
7.63
7.61
7.60
7.60
7.59
7.53
7.53
7.47
7.23
6.50
3.26
2.51
2.50
2.50
2.49
S
O
N
H
O
0.99
1.00
1.05
1.03
1.04
6.34
1.06
1.03
14
13
12
11
10
9
8
7
6
5
4
3
2
1
0
Chemical Shift (ppm)

^{1}H NMR of **3i**

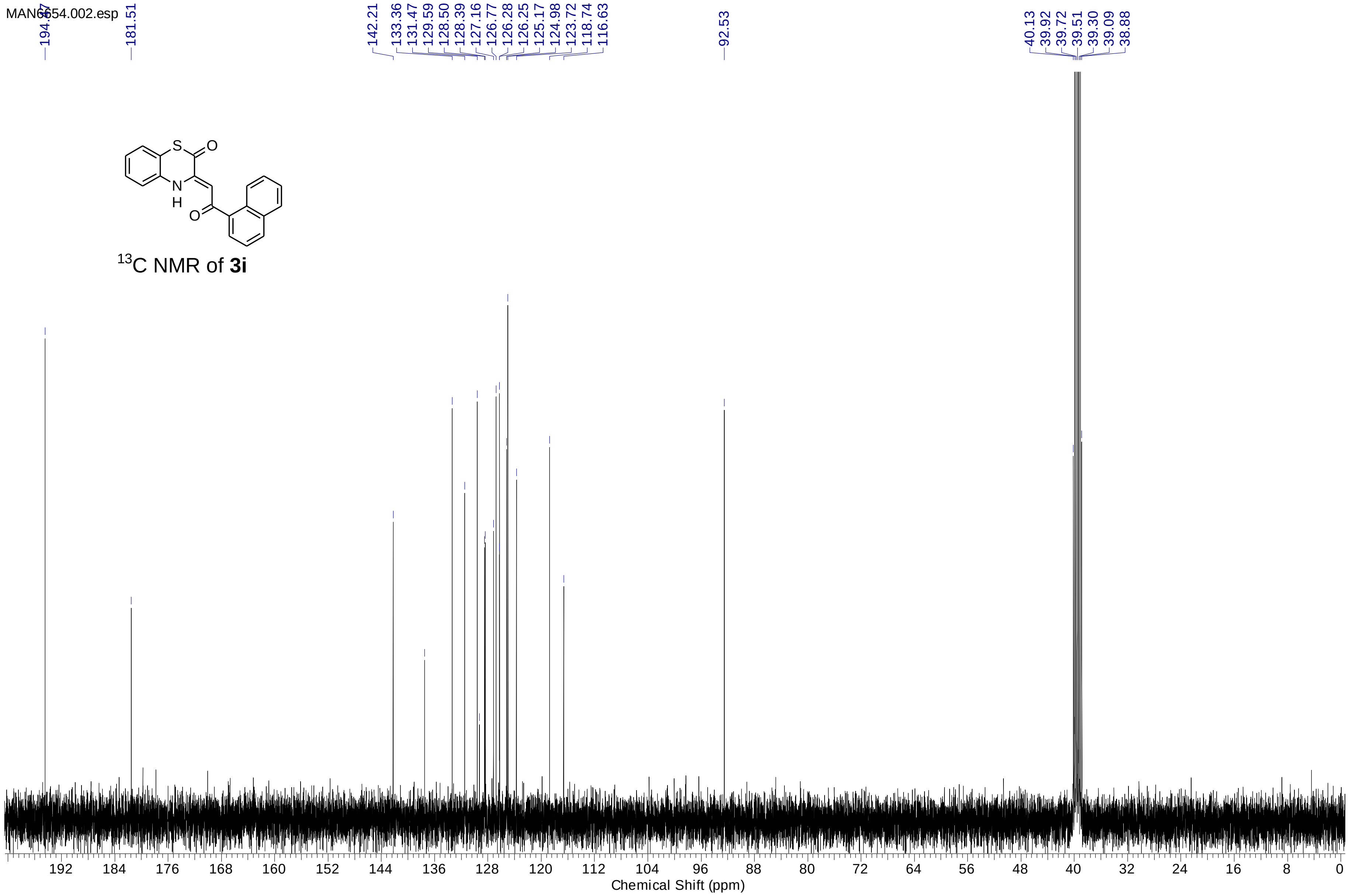
MAN6654.002.esp
194.47
181.51
142.21
133.36
131.47
129.59
128.50
128.39
127.16
126.77
126.28
126.25
125.17
124.98
123.72
118.74
116.63
92.53
40.13
39.92
39.72
39.51
39.30
39.09
38.88
192 184 176 168 160 152 144 136 128 120 112 104 96 88 80 72 64 56 48 40 32 24 16 8 0
Chemical Shift (ppm)

^{13}C NMR of **3i**

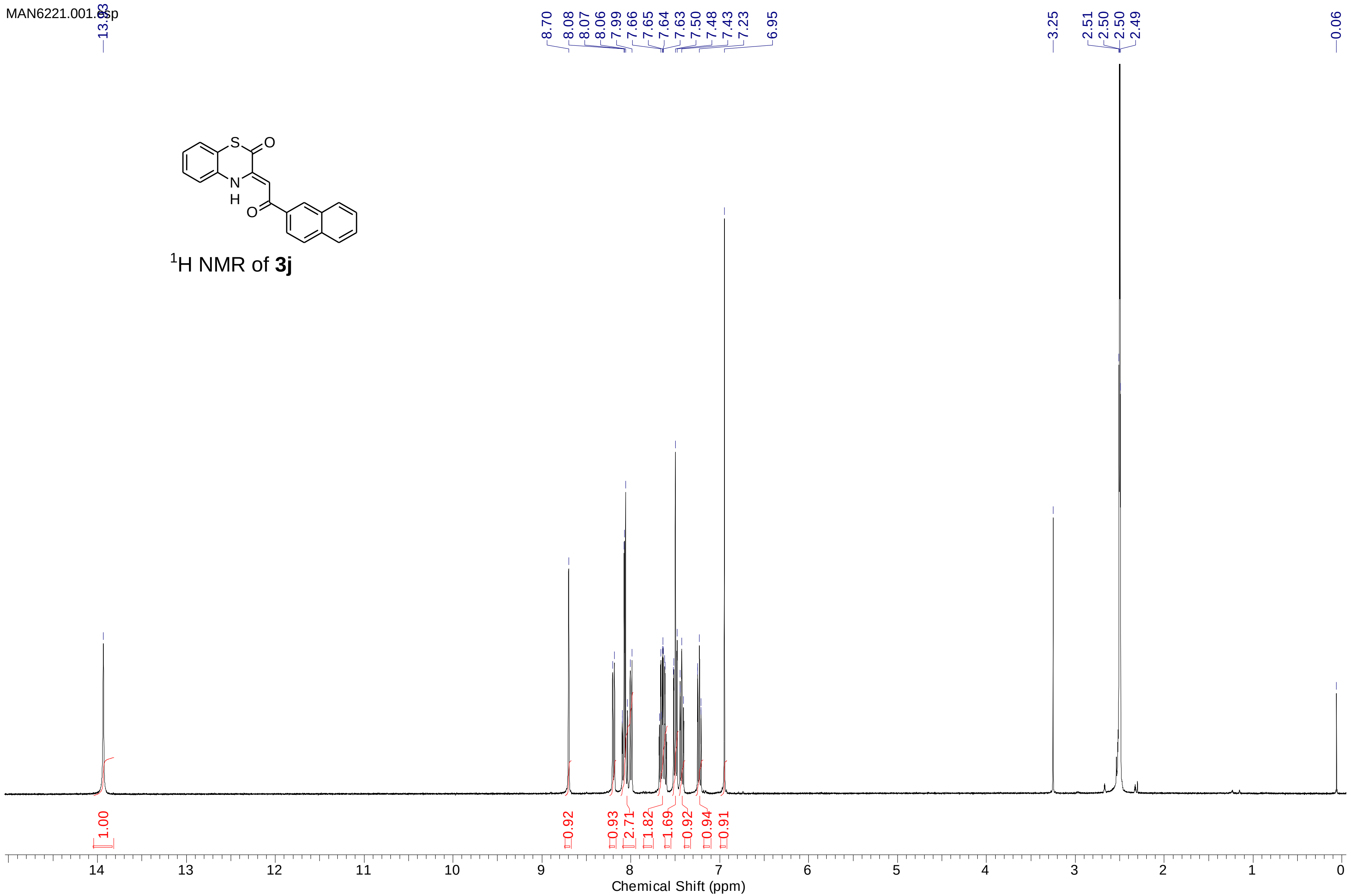
MAN6221.001.esp
13.93
8.70 8.08 8.07 8.06 7.99 7.66 7.65 7.64 7.63 7.50 7.48 7.43 7.23 6.95
3.25
2.51 2.50 2.50 2.49
0.06
S
O
N
H
O
^{1}H NMR of **3j**
1.00
0.92 0.93 2.71 1.82 1.69 0.92 0.94 0.91
14 13 12 11 10 9 8 7 6 5 4 3 2 1 0
Chemical Shift (ppm)

MAN6221.002.esp

^{13}C NMR of **3j**

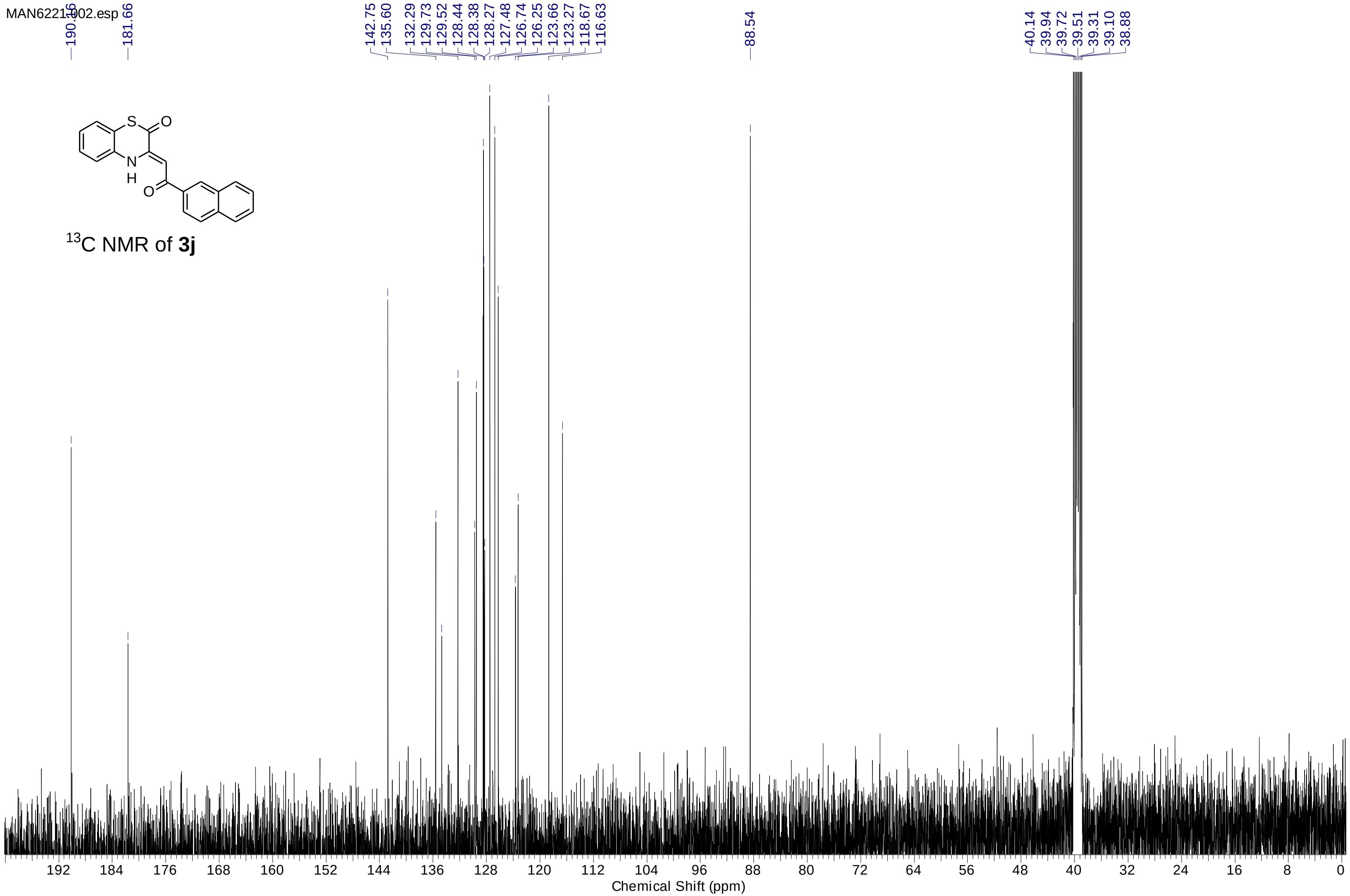

MAN6655.001.esp

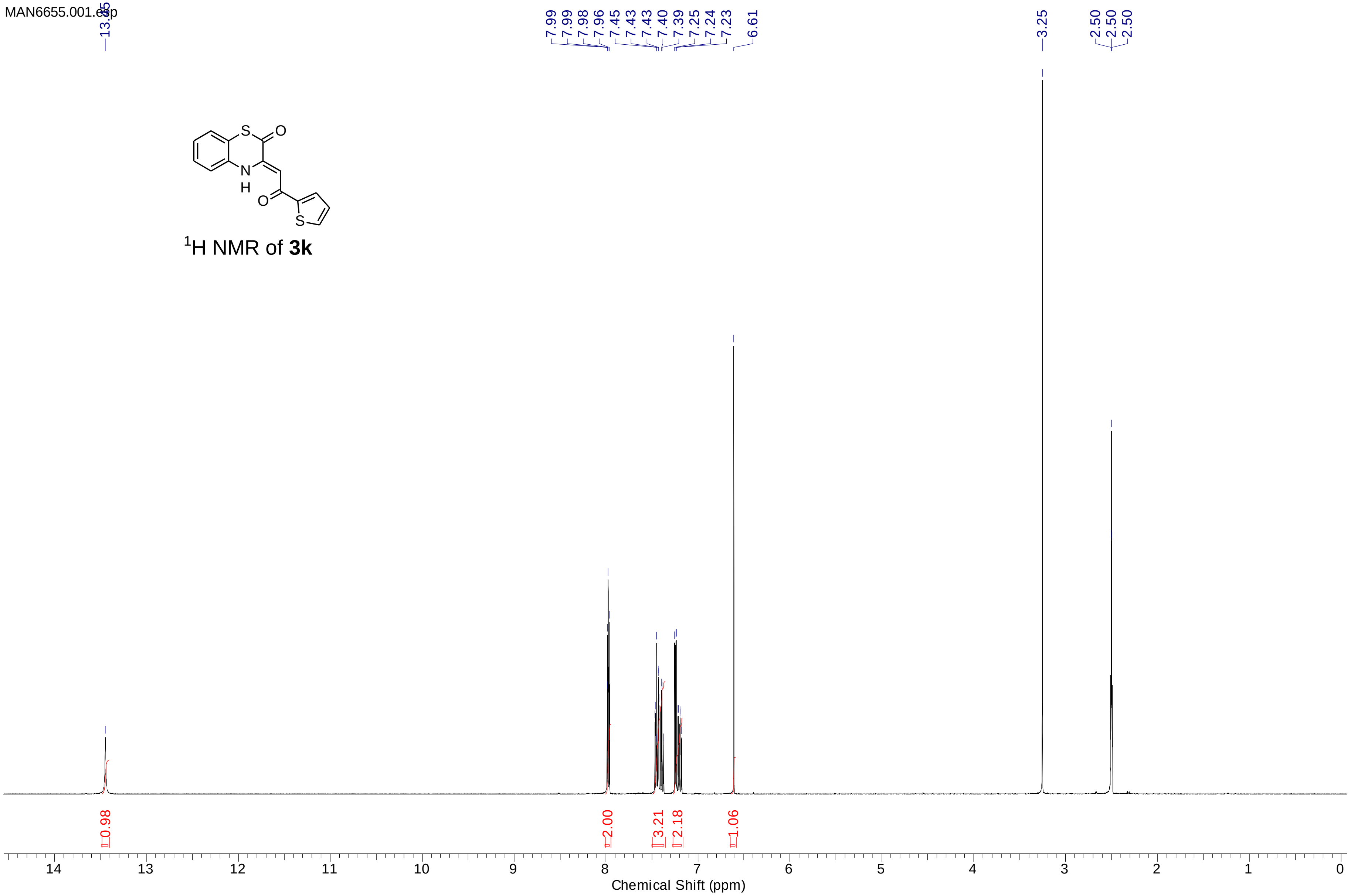

^{1}H NMR of **3k**

MAN6655.002.esp

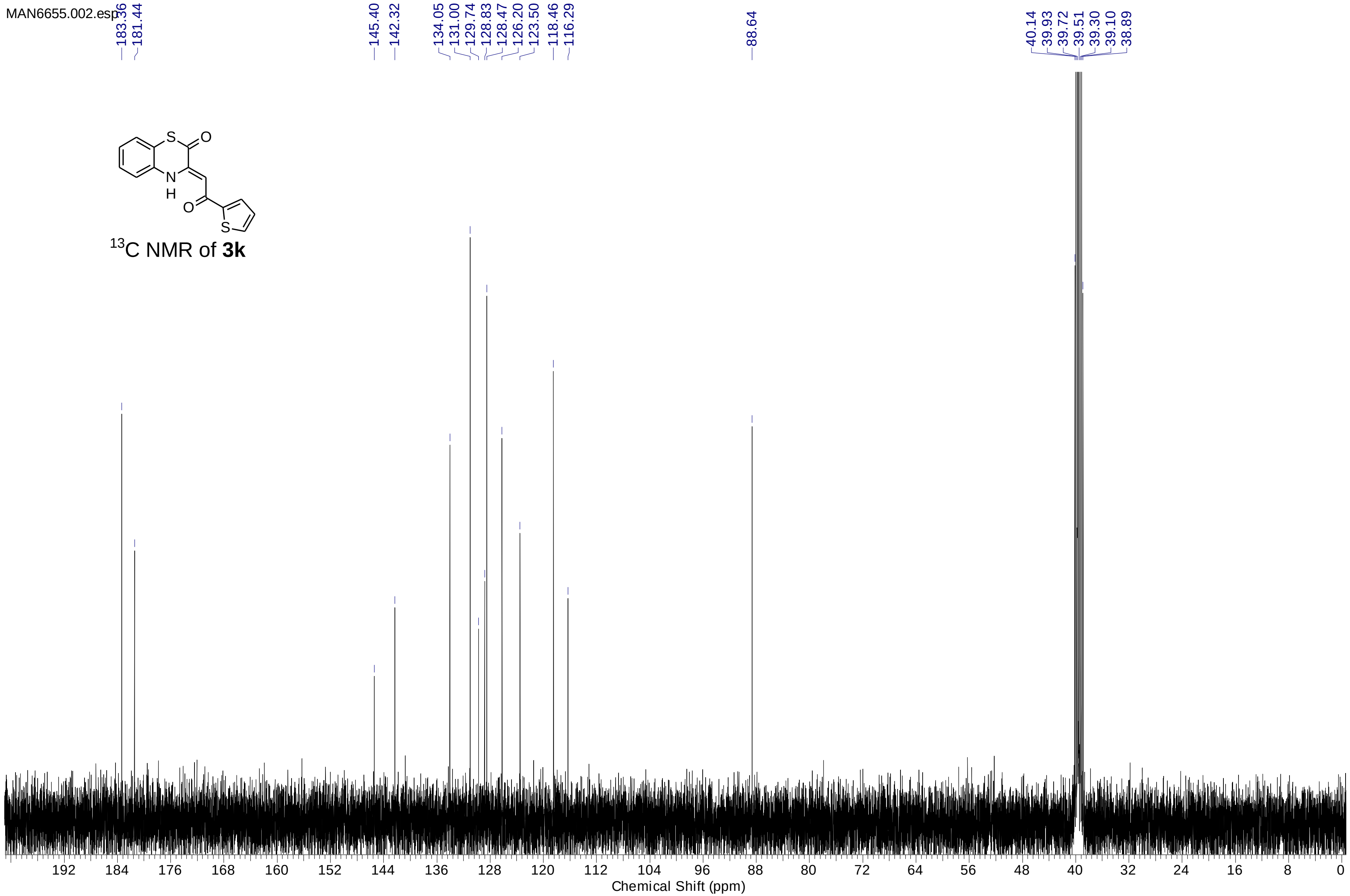

^{13}C NMR of **3k**

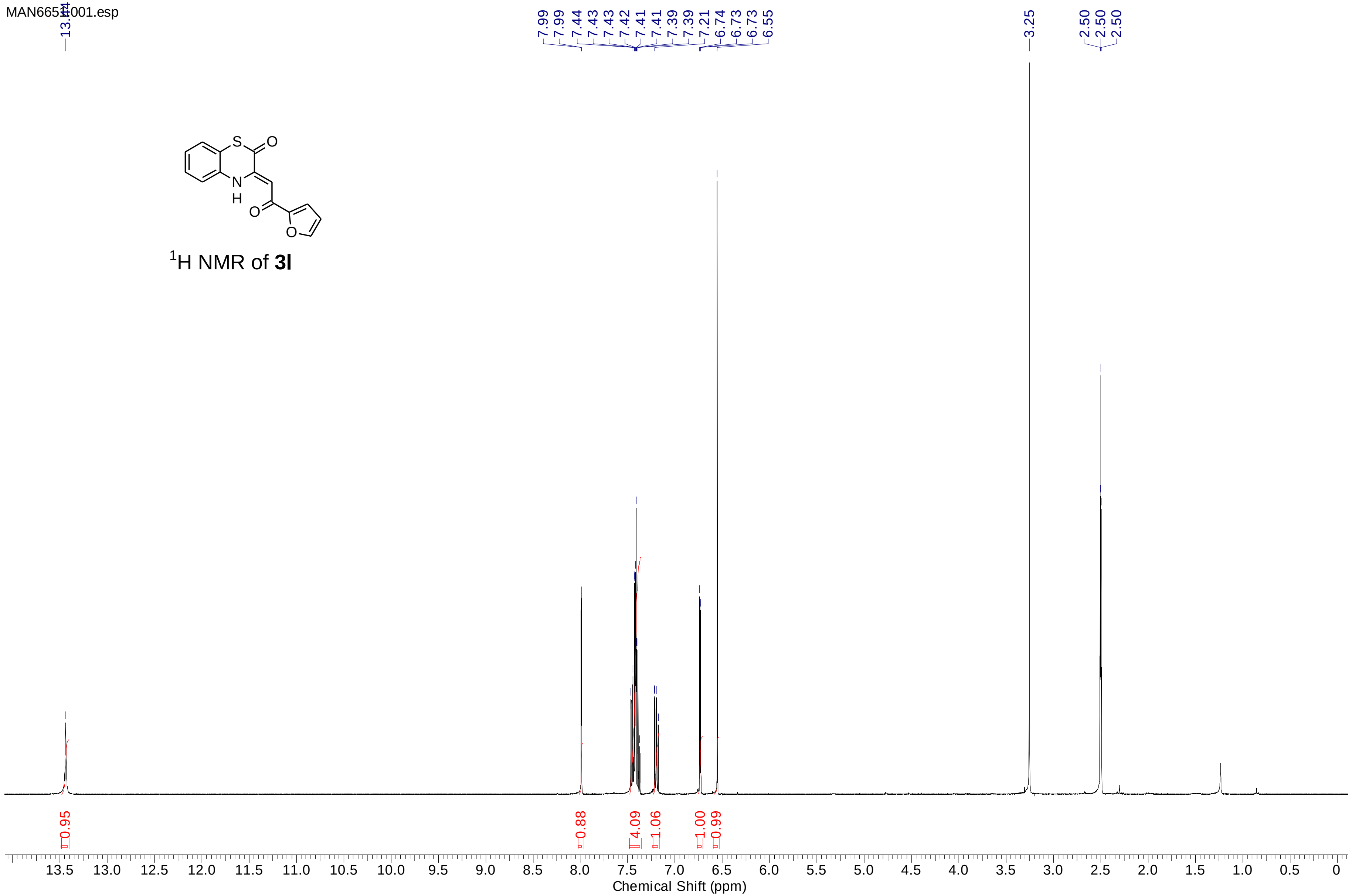
MAN6651-001.esp
13.44
7.99
7.99
7.44
7.43
7.43
7.42
7.41
7.41
7.39
7.39
7.21
6.74
6.73
6.73
6.55
3.25
2.50
2.50
2.50
0.95
0.88
4.09
1.06
1.00
0.99
13.5
13.0
12.5
12.0
11.5
11.0
10.5
10.0
9.5
9.0
8.5
8.0
7.5
7.0
6.5
6.0
5.5
5.0
4.5
4.0
3.5
3.0
2.5
2.0
1.5
1.0
0.5
0
Chemical Shift (ppm)

^{1}H NMR of **3l**

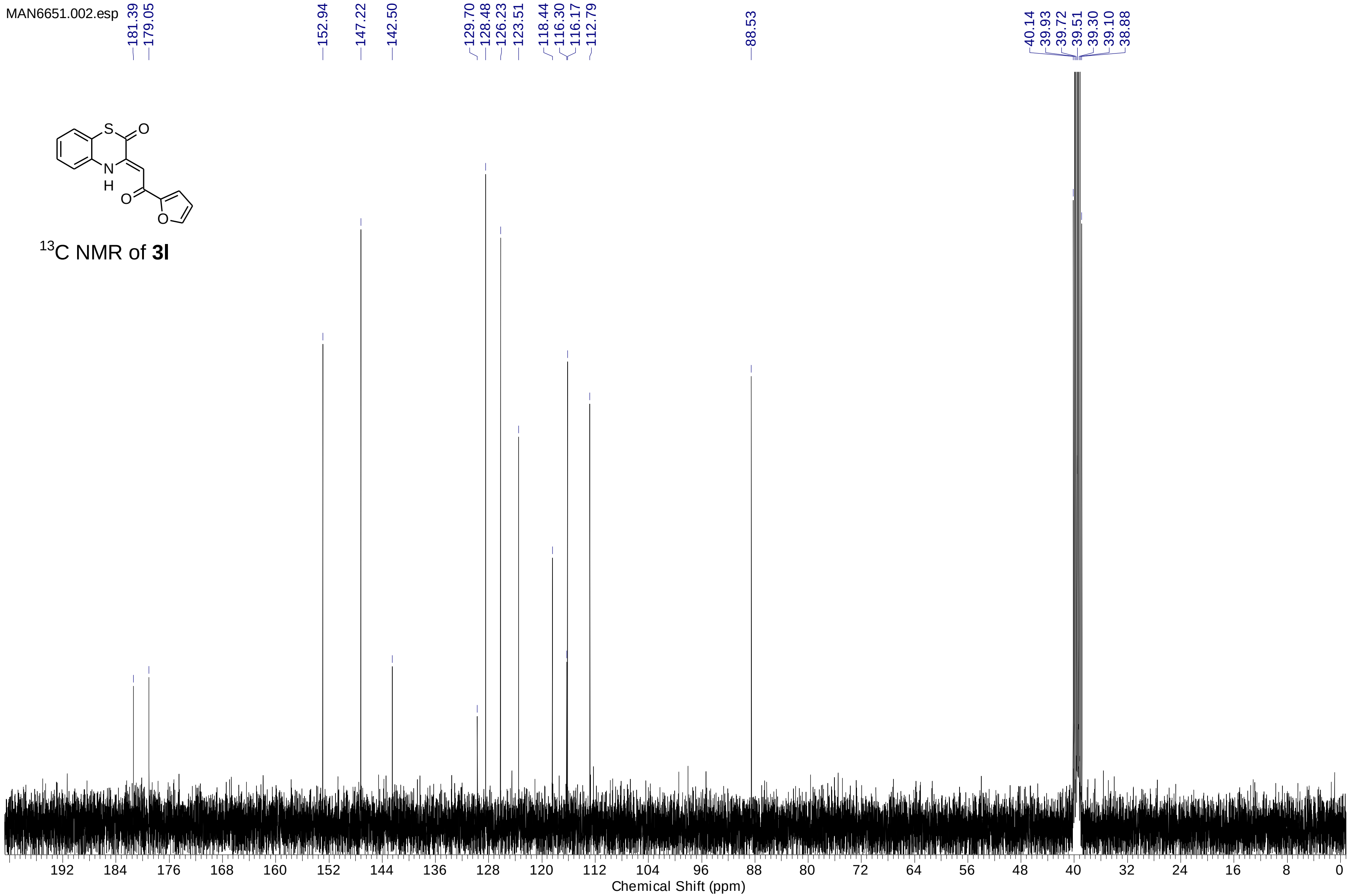
MAN6651.002.esp
181.39
179.05
152.94
147.22
142.50
129.70
128.48
126.23
123.51
118.44
116.30
116.17
112.79
88.53
40.14
39.93
39.72
39.51
39.30
39.10
38.88
S
O
N
H
O
O
192 184 176 168 160 152 144 136 128 120 112 104 96 88 80 72 64 56 48 40 32 24 16 8 0
Chemical Shift (ppm)

^{13}C NMR of **3l**

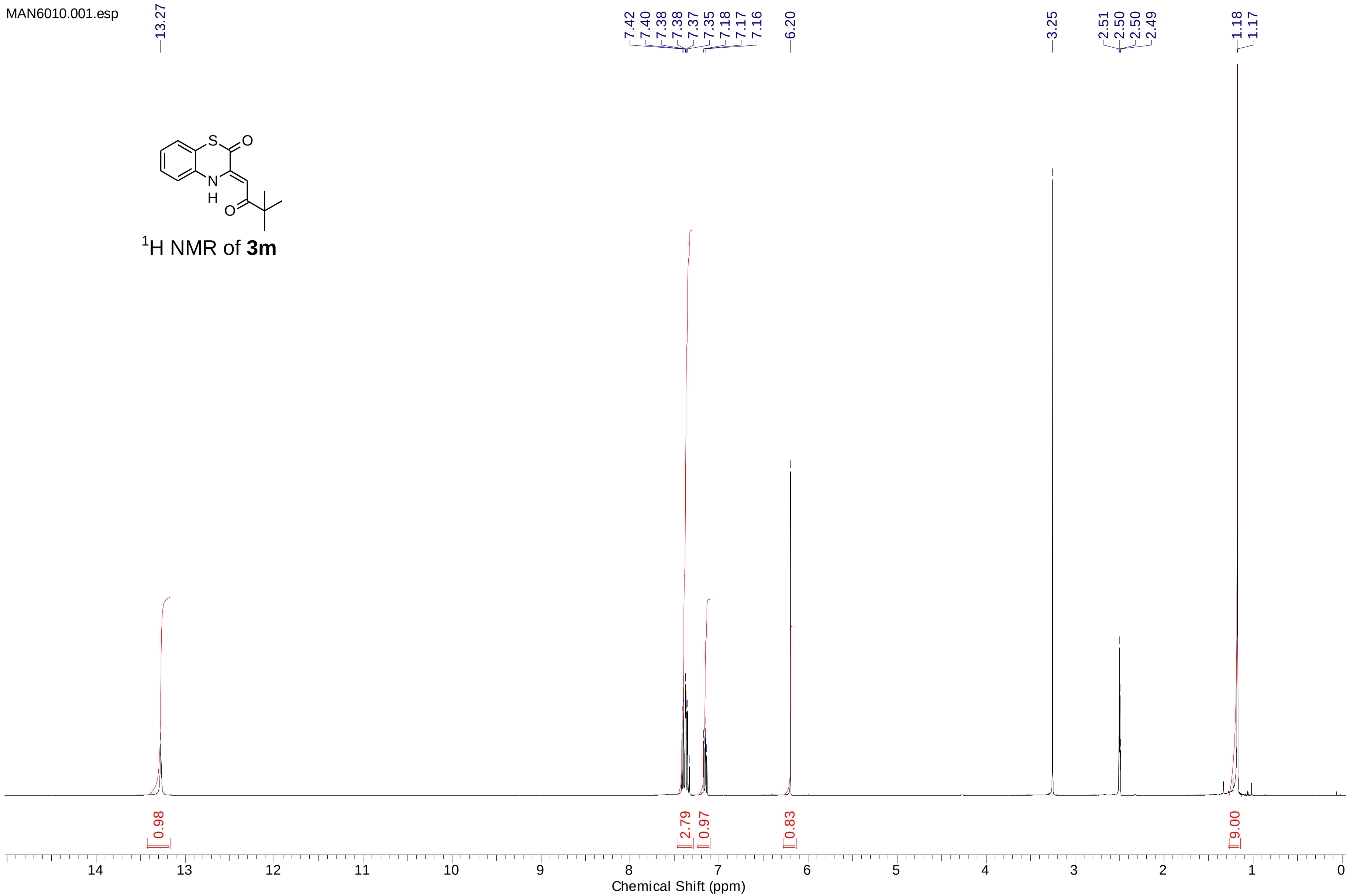
MAN6010.001.esp
13.27
7.42 7.40 7.38 7.38 7.37 7.35 7.18 7.17 7.16
6.20
3.25
2.51 2.50 2.50 2.49
1.18 1.17
S
O
N
H
O
^{1}H NMR of **3m**
0.98
2.79
0.97
0.83
9.00
14 13 12 11 10 9 8 7 6 5 4 3 2 1 0
Chemical Shift (ppm)

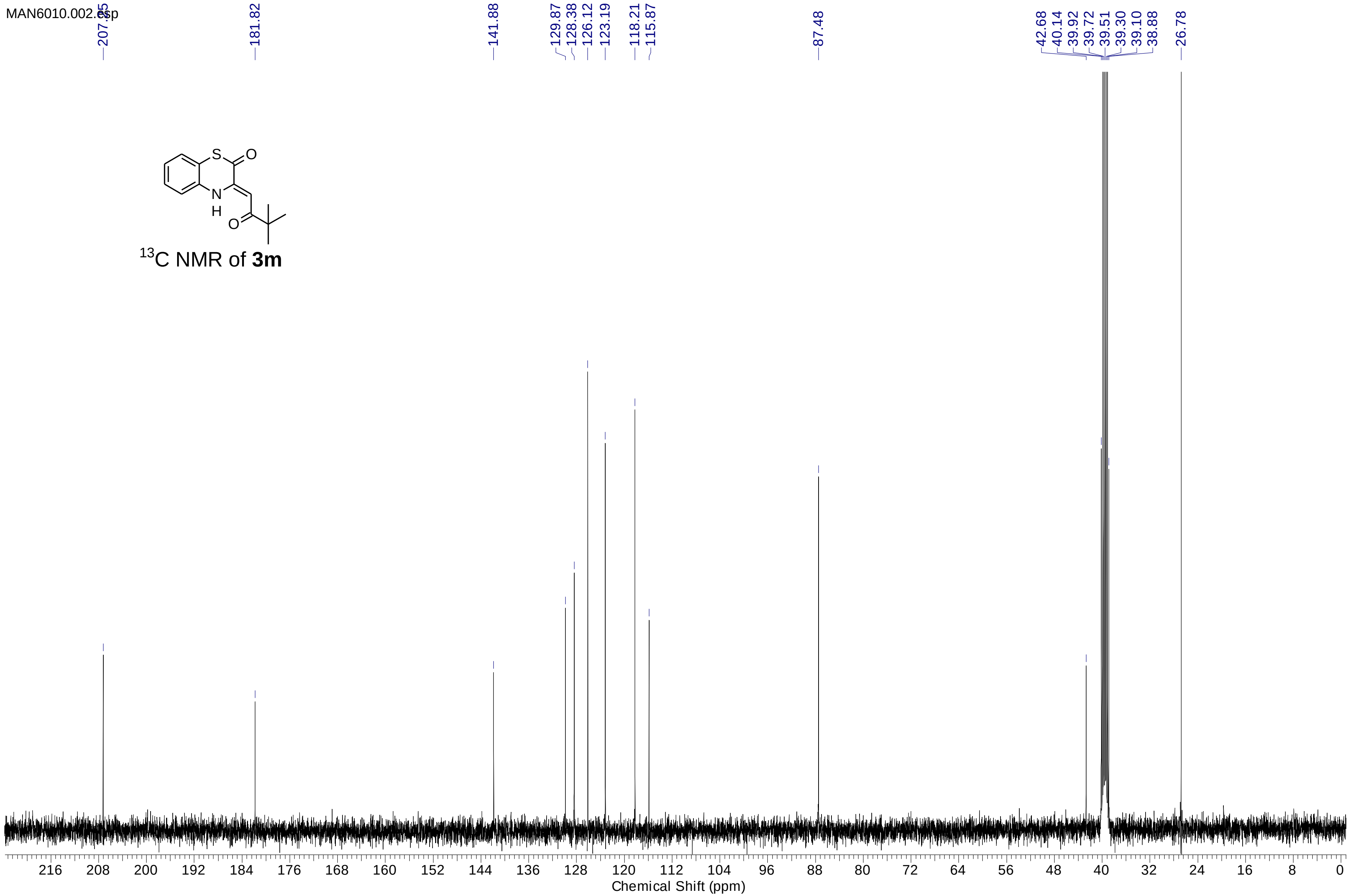
MAN6010.002.esp
207.25
181.82
141.88
129.87
128.38
126.12
123.19
118.21
115.87
87.48
42.68
40.14
39.92
39.72
39.51
39.30
39.10
38.88
26.78
S
O
N
H
O
^{13}C NMR of **3m**
216
208
200
192
184
176
168
160
152
144
136
128
120
112
104
96
88
80
72
64
56
48
40
32
24
16
8
0
Chemical Shift (ppm)

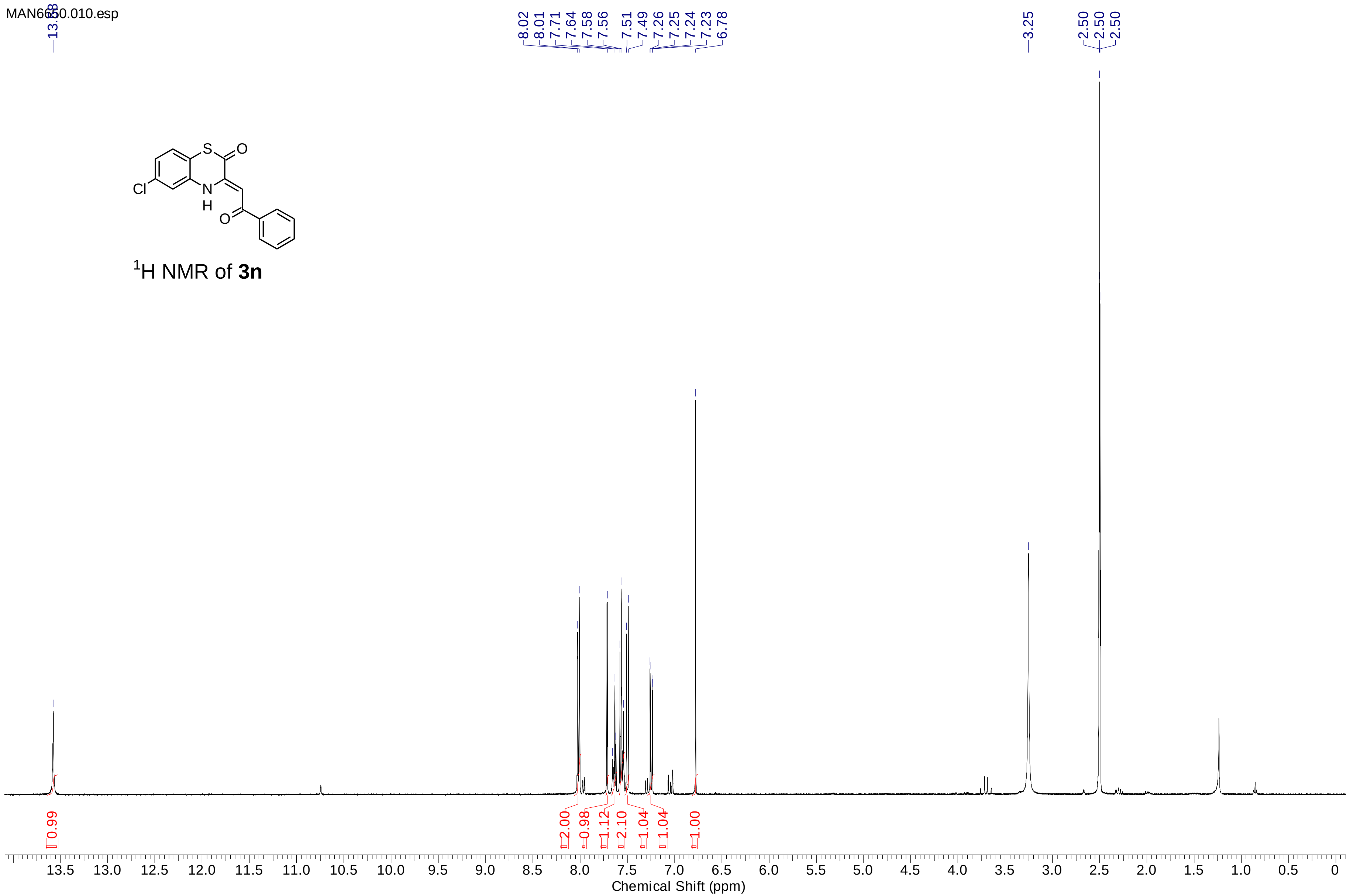
MAN6650.010.esp
13.58
8.02
8.01
7.71
7.64
7.58
7.56
7.51
7.49
7.26
7.25
7.24
7.23
6.78
3.25
2.50
2.50
2.50
Cl
S
O
N
H
O
^{1}H NMR of **3n**
0.99
2.00
0.98
1.12
2.10
1.04
1.04
1.00
13.5 13.0 12.5 12.0 11.5 11.0 10.5 10.0 9.5 9.0 8.5 8.0 7.5 7.0 6.5 6.0 5.5 5.0 4.5 4.0 3.5 3.0 2.5 2.0 1.5 1.0 0.5 0
Chemical Shift (ppm)

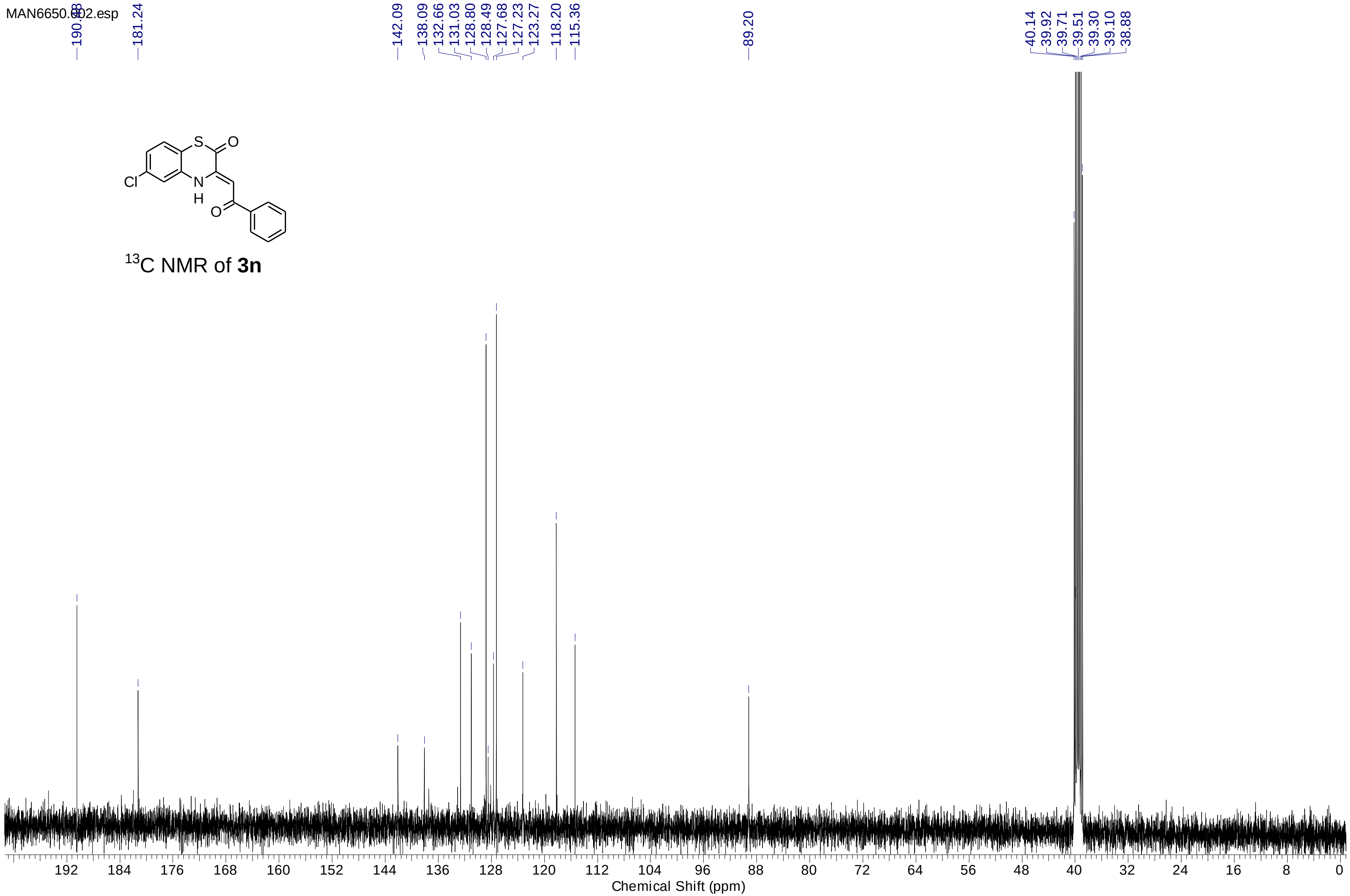
MAN6650.002.esp
190.48
181.24
142.09
138.09
132.66
131.03
128.80
128.49
127.68
127.23
123.27
118.20
115.36
89.20
40.14
39.92
39.71
39.51
39.30
39.10
38.88
S
O
Cl
N
H
O
192 184 176 168 160 152 144 136 128 120 112 104 96 88 80 72 64 56 48 40 32 24 16 8 0
Chemical Shift (ppm)

^{13}C NMR of **3n**

2-Hydroxy-2*H*-1,4-benzothiazin-3(4*H*)-ones 4a–n. General procedure.

To a stirring solution of furandione **5** (1 mmol) in 1,4-dioxane (3 mL), *o*-aminothiophenol (**1**, 1.05 mmol) was added. The mixture was stirred at room temperature overnight. Then the solvent was removed under vacuum. The solid residue was stirred with toluene (2–3 mL) for 1 h. Then the precipitate was filtered off and washed with acetone (2-3 mL) to afford the target compound **4**.

It should be mentioned that compounds **4a–n** are thermally stable. They did not eliminate water even when they were heated up to 110 °C (boiling toluene).

2-Hydroxy-2-(2-oxo-2-phenylethyl)-2*H*-1,4-benzothiazin-3(4*H*)-one (4a)

Yield: 228 mg (76%); pale yellow solid; mp 183–185 °C.

^{1}H NMR (400 MHz, DMSO-d_6): δ = 10.64 (s, 1 H), 7.97 (m, 2 H), 7.64 (m, 1 H), 7.53 (m, 2 H), 7.27 (m, 1 H), 7.19 (m, 1 H), 7.05 (m, 1 H), 6.98 (m, 1 H), 6.90 (s, 1 H), 3.70 (dd, *J* = 15.8, 33.6 Hz, 2 H) ppm.

^{13}C NMR (100 MHz, DMSO-d_6): δ = 195.9, 164.1, 137.2, 136.3, 133.1, 128.5 (2 C), 128.2 (2 C), 127.5, 126.4, 122.6, 118.4, 116.6, 76.8, 44.4 ppm.

IR (mineral oil): 3314, 3175, 1653 cm^{-1}.

MS (ESI+): m/z calcd for $C_{16}H_{13}NO_3S+H^+$: 300.07 [$M+H^+$]; found: 300.09.

Anal. Calcd (%) for $C_{16}H_{13}NO_3S$: C 64.20; H 4.38; N 4.68. Found: C 63.99; H 4.34; N 4.60.

λ_{max} = 240 nm.

2-(2-(4-Methylphenyl)-2-oxoethyl)-2-hydroxy-2*H*-1,4-benzothiazin-3(4*H*)-one (4b)

Yield: 204 mg (65%); pale yellow solid; mp 174–175 °C.

^{1}H NMR (400 MHz, DMSO-d_6): δ = 10.63 (s, 1 H), 7.87 (m, 2 H), 7.33 (m, 2 H), 7.26 (m, 1 H), 7.18 (m, 1 H), 7.05 (m, 1 H), 6.98 (m, 1 H), 6.86 (s, 1 H), 3.66 (dd, *J* = 16.3, 30.3 Hz, 2 H), 2.38 (s, 3 H) ppm.

^{13}C NMR (100 MHz, DMSO-d_6): δ = 195.4, 164.1, 143.5, 136.3, 134.7, 129.0 (2 C), 128.3 (2 C), 127.5, 126.4, 122.6, 118.4, 116.6, 76.8, 44.2, 21.0 ppm.

IR (mineral oil): 3322, 3186 cm^{-1}.

MS (ESI+): m/z calcd for $C_{17}H_{15}NO_3S+H^+$: 314.09 [$M+H^+$]; found: 314.12.

Anal. Calcd (%) for $C_{17}H_{15}NO_3S$: C 65.16; H 4.82; N 4.47. Found: C 65.34; H 4.84; N 4.51.

λ_{max} = 256 nm.

2-(2-(4-Methoxyphenyl)-2-oxoethyl)-2-hydroxy-2*H*-1,4-benzothiazin-3(4*H*)-one (4c)

Yield: 267 mg (81%); pale yellow solid; mp 169–171 °C.

^{1}H NMR (400 MHz, DMSO-d_6): δ = 10.62 (s, 1 H), 7.96 (m, 2 H), 7.26 (m, 1 H), 7.19 (m, 1 H), 7.04 (m, 3 H), 6.98 (m, 1 H), 6.85 (s, 1 H), 3.85 (s, 3 H), 3.63 (dd, *J* = 15.9, 28.6 Hz, 2 H) ppm.

^{13}C NMR (100 MHz, DMSO-d_6): δ = 194.4, 164.2, 163.2, 136.3, 130.6 (2 C), 130.1, 127.5, 126.4, 122.6, 118.5, 116.6, 113.7 (2 C), 76.8, 55.5, 43.9 ppm.

IR (mineral oil): 3303, 3183, 1672 cm^{-1}.

MS (ESI+): m/z calcd for $C_{17}H_{15}NO_4S+H^+$: 330.08 [M+H$^+$]; found: 330.11.

Anal. Calcd (%) for $C_{17}H_{15}NO_4S$: C 61.99; H 4.59; N 4.25. Found: C 62.34; H 4.51; N 4.25.

λ_{max} = 280 nm.

2-(2-(4-Ethoxyphenyl)-2-oxoethyl)-2-hydroxy-2*H*-1,4-benzothiazin-3(4*H*)-one (4d)

Yield: 285 mg (83%); pale yellow solid; mp 163–165 °C.

^{1}H NMR (400 MHz, DMSO-d_6): δ = 10.61 (s, 1 H), 7.94 (m, 2 H), 7.26 (m, 1 H), 7.18 (m, 1 H), 7.05-6.96 (m, 4 H), 6.84 (s, 1 H), 4.13 (q, *J* = 7.0 Hz, 2 H), 3.62 (dd, *J* = 15.8, 27.4 Hz, 2 H), 1.35 (t, *J* = 7.0 Hz, 3 H) ppm.

^{13}C NMR (100 MHz, DMSO-d_6): δ = 194.4, 164.1, 162.5, 136.3, 130.6, 129.9, 127.4, 126.4, 122.6, 118.4, 116.5, 114.1, 76.8, 63.5, 43.9, 14.3 ppm.

IR (mineral oil): 3276, 3184, 1678, 1643 cm^{-1}.

MS (ESI+): m/z calcd for $C_{18}H_{17}NO_4S+H^+$: 344.10 [M+H$^+$]; found: 344.14.

Anal. Calcd (%) for $C_{18}H_{17}NO_4S$: C 62.96; H 4.99; N 4.08. Found: C 62.81; H 5.11; N 4.16.

λ_{max} = 282 nm.

2-(2-(4-Fluorophenyl)-2-oxoethyl)-2-hydroxy-2*H*-1,4-benzothiazin-3(4*H*)-one (4e)

Yield: 213 mg (67%); pale yellow solid; mp 184–186 °C.

^{1}H NMR (400 MHz, DMSO-d_6): δ = 10.61 (s, 1 H), 8.04 (m, 2 H), 7.34 (m, 2 H), 7.26 (m, 1 H), 7.18 (m, 1 H), 7.04-6.96 (m, 2 H), 6.90 (s, 1 H), 3.65 (dd, *J* = 15.9, 28.4 Hz, 2 H) ppm.

^{13}C NMR (100 MHz, DMSO-d_6): δ = 194.6, 166.3, 164.2, 163.8, 136.3, 134.1, 134.0, 131.4, 131.3, 127.6, 126.6, 122.7, 118.5, 116.7, 115.6, 115.4, 76.9, 44.5 ppm.

^{19}F NMR (377 MHz, DMSO-d_6): δ = 106.44 ppm.
IR (mineral oil): 3326, 3178, 1695, 1651 cm^{-1}.
MS (ESI+): m/z calcd for $C_{16}H_{12}FNO_3S+H^+$: 318.06 [M+H$^+$]; found: 318.08.
Anal. Calcd (%) for $C_{16}H_{12}FNO_3S$: C 60.56; H 3.81; N 4.41. Found: C 60.67; H 3.84; N 4.35.
λ_{max} = 240 nm.

2-(2-(4-Chlorophenyl)-2-oxoethyl)-2-hydroxy-2*H*-1,4-benzothiazin-3(4*H*)-one (4f)
Yield: 243 mg (73%); pale yellow solid; mp 172–173 °C.
^1H NMR (400 MHz, DMSO-d_6): δ = 10.65 (s, 1 H), 7.98 (m, 2 H), 7.58 (m, 2 H), 7.26 (m, 1 H), 7.19 (m, 1 H), 7.05 (m, 1 H), 6.98 (m, 2 H), 3.67 (dd, *J* = 15.9, 25.6 Hz, 2 H) ppm.
^{13}C NMR (100 MHz, DMSO-d_6): δ = 194.9, 164.1, 138.1, 136.3, 135.9, 130.1 (2 C), 128.6 (2 C), 127.5, 126.5, 122.7, 118.4, 116.6, 76.8, 44.5 ppm.
IR (mineral oil): 3332, 3179, 1670, 1652 cm^{-1}.
MS (ESI+): m/z calcd for $C_{16}H_{12}ClNO_3S+H^+$: 334.03 [M+H$^+$]; found: 334.04.
Anal. Calcd (%) for $C_{16}H_{12}ClNO_3S$: C 57.57; H 3.62; N 4.20. Found: C 57.42; H 3.63; N 4.18.
λ_{max} = 256 nm.

2-(2-(4-Bromophenyl)-2-oxoethyl)-2-hydroxy-2*H*-1,4-benzothiazin-3(4*H*)-one (4g)
Yield: 294 mg (78%); pale yellow solid; mp 192–194 °C.
^1H NMR (400 MHz, DMSO-d_6): δ = 10.62 (s, 1 H), 7.89 (m, 2 H), 7.74 (m, 2 H), 7.26 (m, 1 H), 7.18 (m, 1 H), 7.04-6.96 (m, 2 H), 6.92 (br.s, 1 H), 3.65 (dd, *J* = 15.9, 24.7 Hz, 2 H) ppm.
^{13}C NMR (100 MHz, DMSO-d_6): δ = 195.1, 164.0, 136.2 (2 C), 131.5 (2 C), 130.2 (2 C), 127.5, 127.2, 126.5, 122.6, 118.4, 116.6, 76.8, 44.4 ppm.
IR (mineral oil): 3410, 3191, 1668 cm^{-1}.
MS (ESI+): m/z calcd for $C_{16}H_{12}BrNO_3S+H^+$: 377.98, 379.98 [M+H$^+$]; found: 377.96, 379.96.
Anal. Calcd (%) for $C_{16}H_{12}BrNO_3S$: C 50.81; H 3.20; N 3.70. Found: C 50.99; H 3.31; N 3.65.
λ_{max} = 256 nm.

2-(2-(4-Nitrophenyl)-2-oxoethyl)-2-hydroxy-2*H*-1,4-benzothiazin-3(4*H*)-one (4h)
was not isolated since it was formed only in trace amounts and detected by UPLC-UV-MS.
MS (ESI+): m/z calcd for $C_{16}H_{12}N_2O_5S+H^+$: 345.05 [M+H$^+$]; found: 344.90.
MS (ESI-): m/z calcd for $C_{16}H_{12}N_2O_5S-H^+$: 343.04 [M+H$^+$]; found: 342.94.
λ_{max} = 250 nm.

2-Hydroxy-2-(2-(naphthalen-1-yl)-2-oxoethyl)-2*H*-1,4-benzothiazin-3(4*H*)-one (4i)
Yield: 241 mg (69%); pale yellow solid; mp 190–191 °C.
^{1}H NMR (400 MHz, DMSO-d_6): δ = 10.65 (s, 1 H), 8.42 (m, 1 H), 8.12 (m, 1 H), 8.05 (m, 1 H), 8.00 (m, 1 H), 7.59 (m, 3 H), 7.26 (m, 1 H), 7.20 (m, 1 H), 7.01 (m, 2 H), 6.95 (s, 1 H), 3.76 (dd, *J* = 15.9, 44.3 Hz, 2 H) ppm.
^{13}C NMR (100 MHz, DMSO-d_6): δ = 199.7, 164.1, 136.4, 136.2, 133.3, 132.0, 129.3, 128.2, 127.8, 127.5, 127.3, 126.5, 126.2, 125.5, 124.6, 122.6, 118.4, 116.6, 77.1, 48.0 ppm.
IR (mineral oil): 3373, 3188, 1681, 1662 cm^{-1}.
MS (ESI+): m/z calcd for $C_{20}H_{15}NO_3S+H^+$: 350.09 [M+H$^+$]; found: 350.09.
Anal. Calcd (%) for $C_{20}H_{15}NO_3S$: C 68.75; H 4.33; N 4.01. Found: C 68.99; H 4.32; N 4.14.
λ_{max} = 300 nm.

2-Hydroxy-2-(2-(naphthalen-2-yl)-2-oxoethyl)-2*H*-1,4-benzothiazin-3(4*H*)-one (4j)
Yield: 238 mg (68%); pale yellow solid; mp 180–182 °C.
^{1}H NMR (400 MHz, DMSO-d_6): δ = 10.66 (s, 1 H), 8.68 (s, 1 H), 8.14 (m, 1 H), 8.00 (m, 3 H), 7.65 (m, 2 H), 7.27 (m, 1 H), 7.19 (m, 1 H), 7.06 (m, 1 H), 6.98 (m, 1 H), 6.94 (s, 1 H), 3.83 (dd, *J* = 15.8, 33.1 Hz, 2 H) ppm.
^{13}C NMR (100 MHz, DMSO-d_6): δ = 195.9, 164.1, 136.3, 134.9, 134.5, 132.0, 130.3, 129.6, 128.6, 128.0, 127.5 (2 C), 126.8, 126.5, 123.6, 122.6, 118.4, 116.6, 76.9, 44.4 ppm.
IR (mineral oil): 3245, 3183, 1668, 1643 cm^{-1}.
MS (ESI+): m/z calcd for $C_{20}H_{15}NO_3S+H^+$: 350.09 [M+H$^+$]; found: 350.06.
Anal. Calcd (%) for $C_{20}H_{15}NO_3S$: C 68.75; H 4.33; N 4.01. Found: C 69.03; H 4.19; N 4.00.
λ_{max} = 285 nm.

2-Hydroxy-2-(2-oxo-2-(thiophen-2-yl)ethyl)-2*H*-1,4-benzothiazin-3(4H)-one (4k)
Yield: 68 mg (22%); pale yellow solid; mp 170–172 °C.
^{1}H NMR (400 MHz, DMSO-d_6): δ = 10.64 (s, 1 H), 8.00 (m, 1 H), 7.95 (m, 1 H), 7.25 (m, 2 H), 7.18 (m, 1 H), 7.04 (m, 1 H), 6.98 (m, 1 H), 6.90 (br.s, 1 H), 3.61 (dd, *J* = 15.5, 23.4 Hz, 2 H) ppm.
^{13}C NMR (100 MHz, DMSO-d_6): δ = 188.6, 164.0, 144.5, 136.2, 135.1, 134.0, 128.6, 127.5, 126.5, 122.6, 118.3, 116.6, 76.5, 45.1 ppm.
IR (mineral oil): 3258, 3178, 1680 cm^{-1}.
MS (ESI+): m/z calcd for $C_{14}H_{11}NO_3S_2$+H$^+$: 306.03 [M+H$^+$]; found: 306.06.
Anal. Calcd (%) for $C_{14}H_{11}NO_3S_2$: C 55.07; H 3.63; N 4.59. Found: C 54.89; H 3.60; N 4.65.
λ_{max} = 258 nm.

2-(2-(Furan-2-yl)-2-oxoethyl)-2-hydroxy-2*H*-1,4-benzothiazin-3(4*H*)-one (4l)
Yield: 70 mg (24%); pale yellow solid; mp 171–173 °C.
^{1}H NMR (400 MHz, DMSO-d_6): δ = 10.63 (s, 1 H), 7.98 (m, 1 H), 7.45 (m, 1 H), 7.26 (m, 1 H), 7.18 (m, 1 H), 7.01 (m, 2 H), 6.88 (s, 1 H), 6.71 (m, 1 H), 3.48 (m, 2 H) ppm.
^{13}C NMR (100 MHz, DMSO-d_6): δ = 183.7, 163.9, 152.3, 147.8, 136.2, 127.5, 126.5, 122.6, 119.0, 118.3, 116.6, 112.5, 76.5, 44.3 ppm.
IR (mineral oil): 3255, 3189, 1676 cm^{-1}.
MS (ESI+): m/z calcd for $C_{14}H_{11}NO_4S$+H$^+$: 290.05 [M+H$^+$]; found: 290.07.
Anal. Calcd (%) for $C_{14}H_{11}NO_4S$: C 58.12; H 3.83; N 4.84. Found: C 58.32; H 3.81; N 4.78.
λ_{max} = 280 nm.

2-(3,3-Dimethyl-2-oxobutyl)-2-hydroxy-2*H*-1,4-benzothiazin-3(4*H*)-one (4m)
was not isolated since it was formed only in trace amounts and detected by UPLC-UV-MS.
MS (ESI+): m/z calcd for $C_{14}H_{17}NO_3S$+Na$^+$: 302.08 [M+H$^+$]; found: 301.96.
MS (ESI-): m/z calcd for $C_{14}H_{17}NO_3S$-H$^+$: 278.09 [M+H$^+$]; found: 278.10.
λ_{max} = 275 nm.

6-Chloro-2-hydroxy-2-(2-oxo-2-phenylethyl)-2*H*-1,4-benzothiazin-3(4*H*)-one (4n)
was not isolated since it was formed only in trace amounts and detected by UPLC-UV-MS.
MS (ESI+): m/z calcd for $C_{16}H_{12}ClNO_3S+Na^+$: 356.01 [M+H$^+$]; found: 355.63.
MS (ESI-): m/z calcd for $C_{16}H_{12}ClNO_3S-H^+$: 332.02 [M+H$^+$]; found: 331.94.
λ_{max} = 297 nm.

MAN6622.001.esp

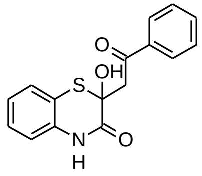

1H NMR of **4a**

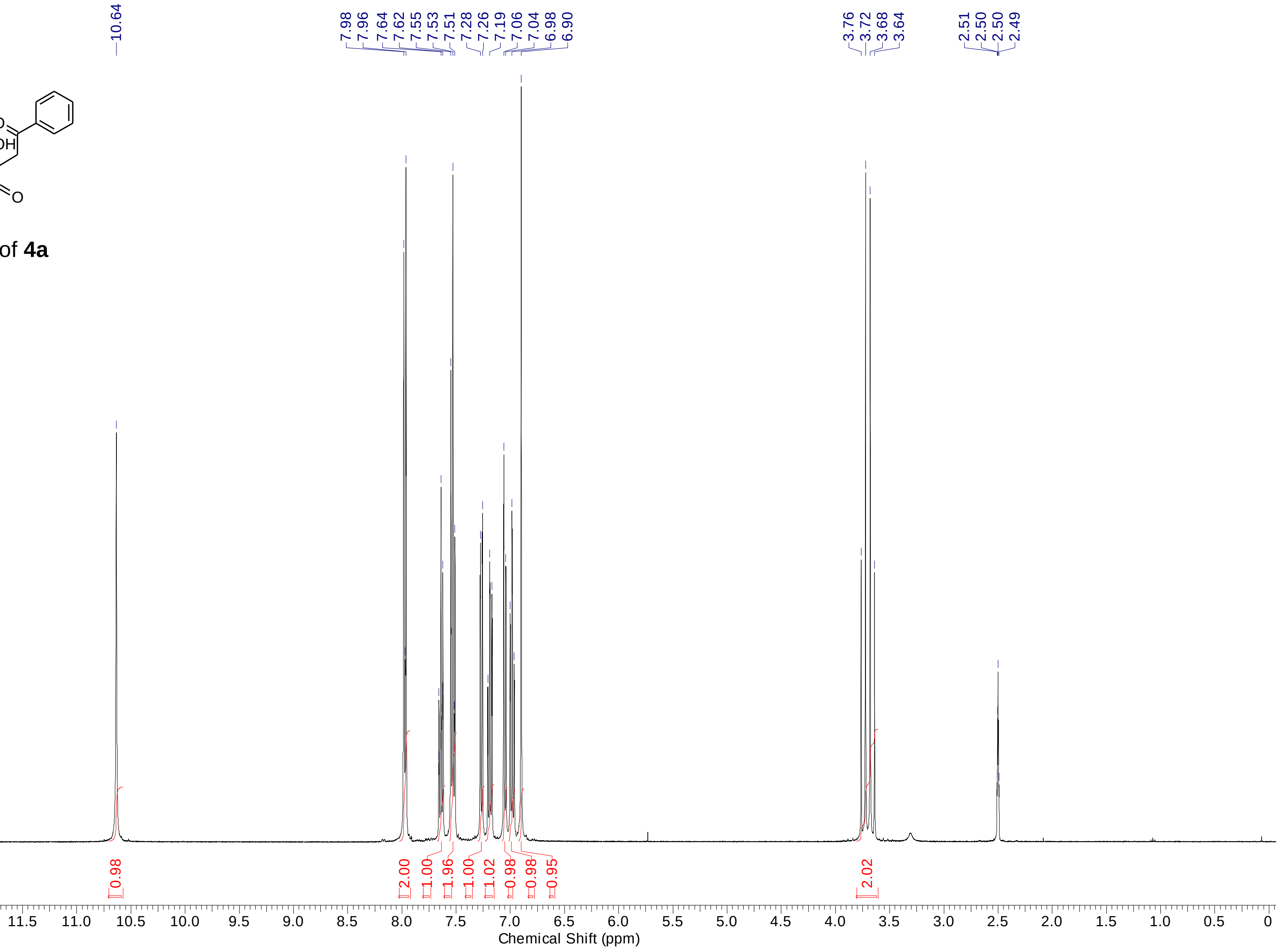

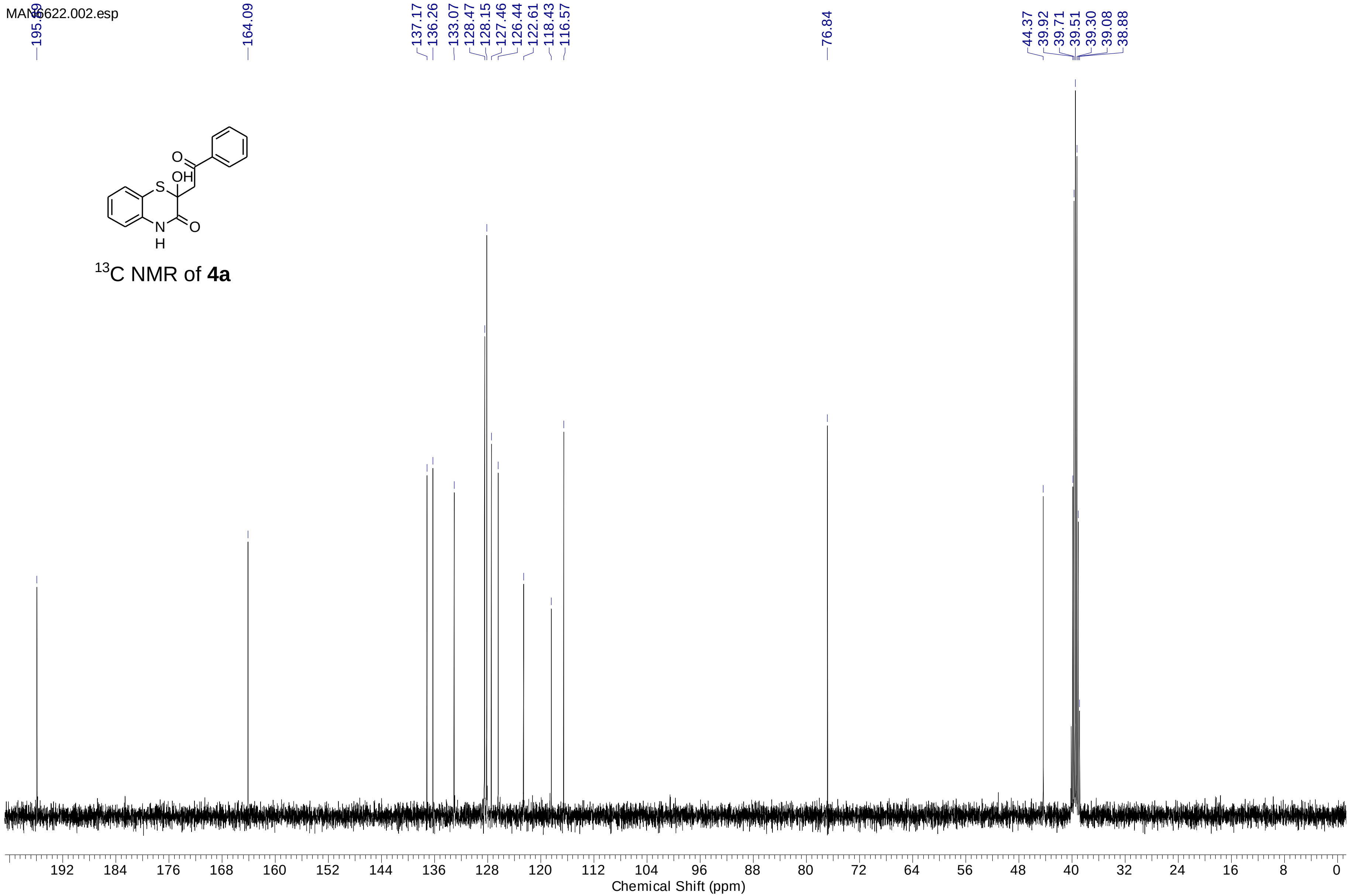
MAN6622.002.esp
195.89
164.09
137.17
136.26
133.07
128.47
128.15
127.46
126.44
122.61
118.43
116.57
76.84
44.37
39.92
39.71
39.51
39.30
39.08
38.88
O
OH
S
N
H
O
192 184 176 168 160 152 144 136 128 120 112 104 96 88 80 72 64 56 48 40 32 24 16 8 0
Chemical Shift (ppm)

^{13}C NMR of **4a**

MAN6626.001.esp

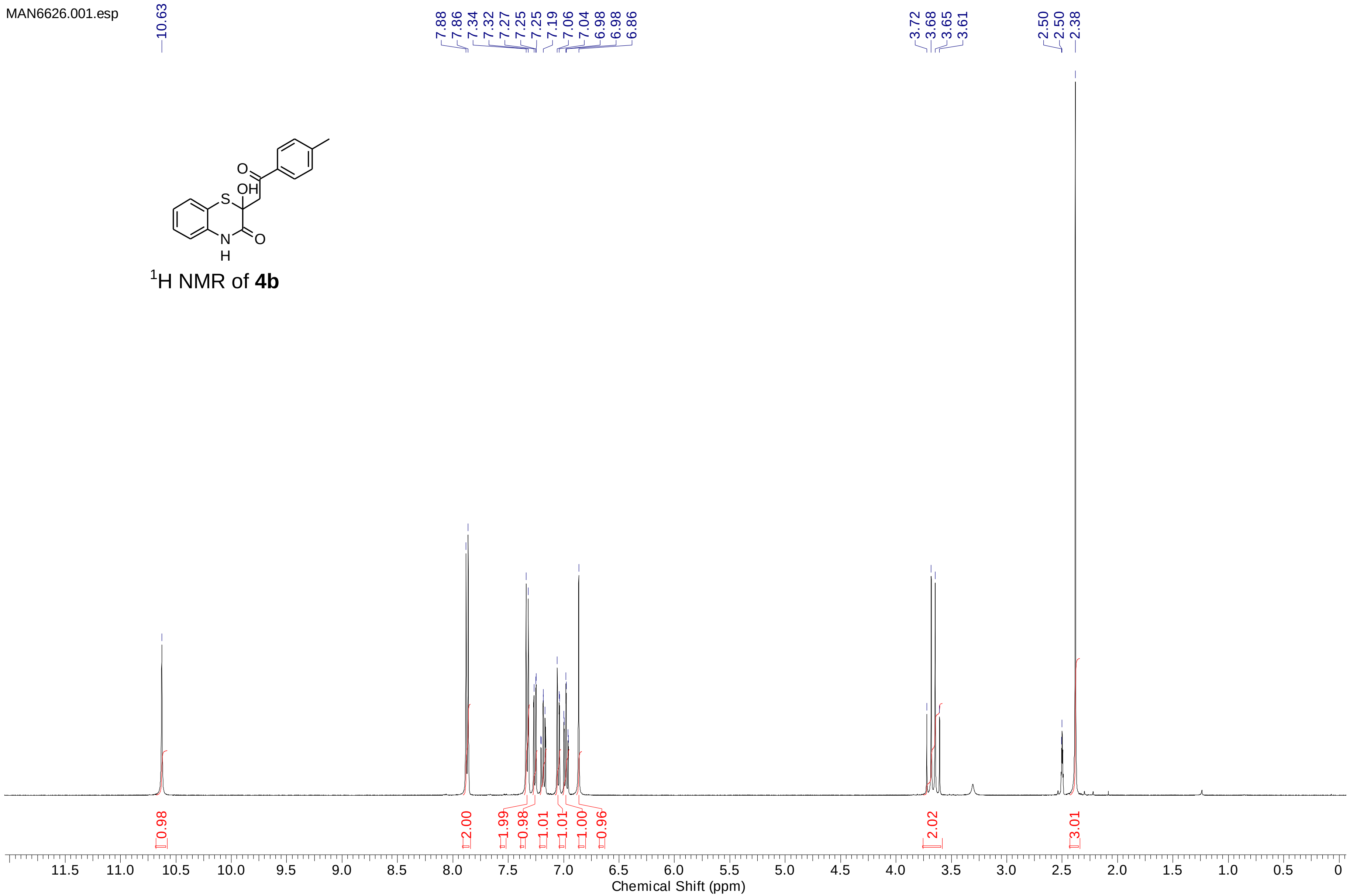

^{1}H NMR of **4b**

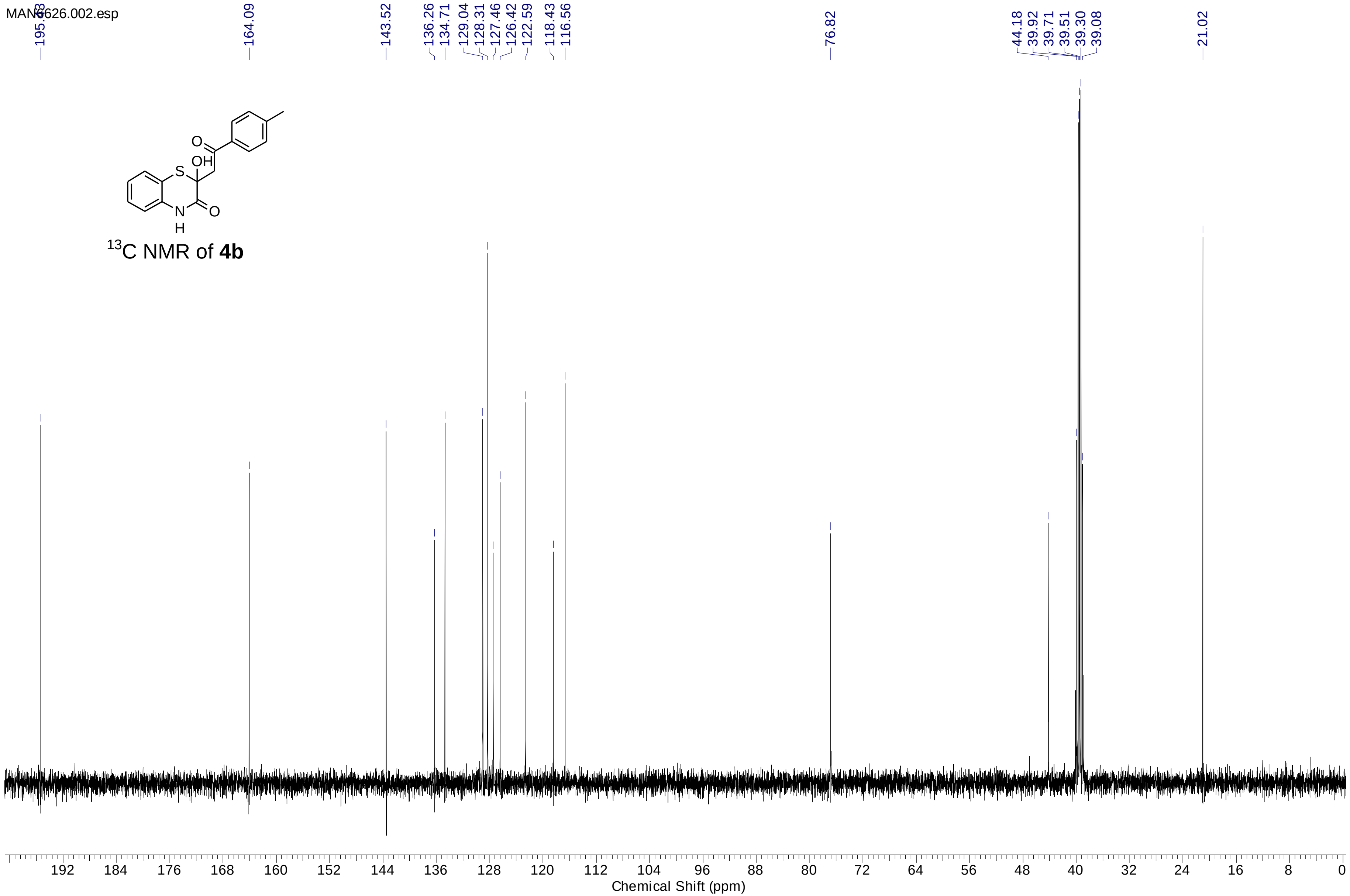

MAN6626.002.esp
195.43
164.09
143.52
136.26
134.71
129.04
128.31
127.46
126.42
122.59
118.43
116.56
76.82
44.18
39.92
39.71
39.51
39.30
39.08
21.02
O
OH
S
N
H
O
192
184
176
168
160
152
144
136
128
120
112
104
96
88
80
72
64
56
48
40
32
24
16
8
0
Chemical Shift (ppm)

^{13}C NMR of **4b**

MAN6626.001.esp

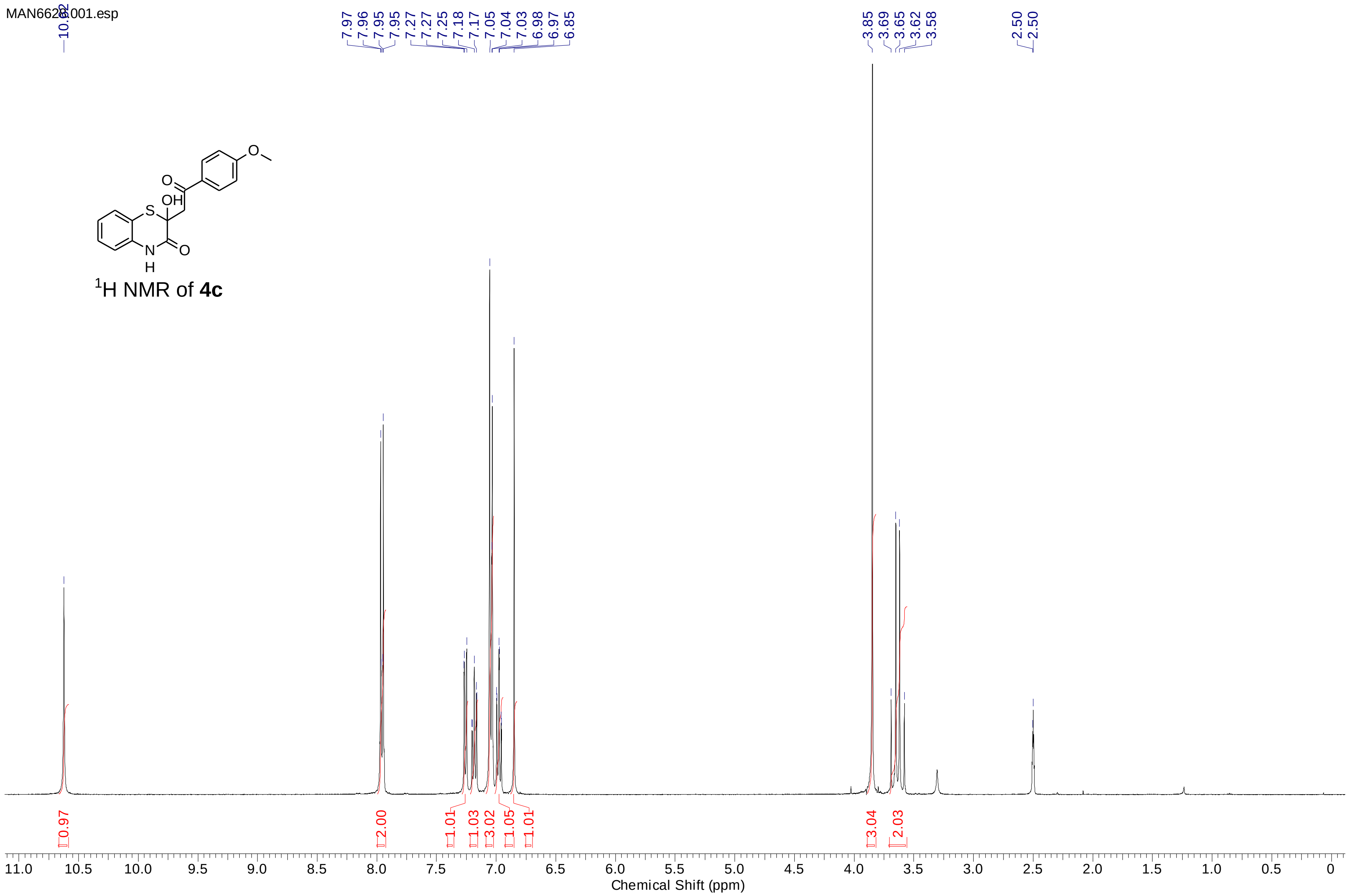

^{1}H NMR of **4c**

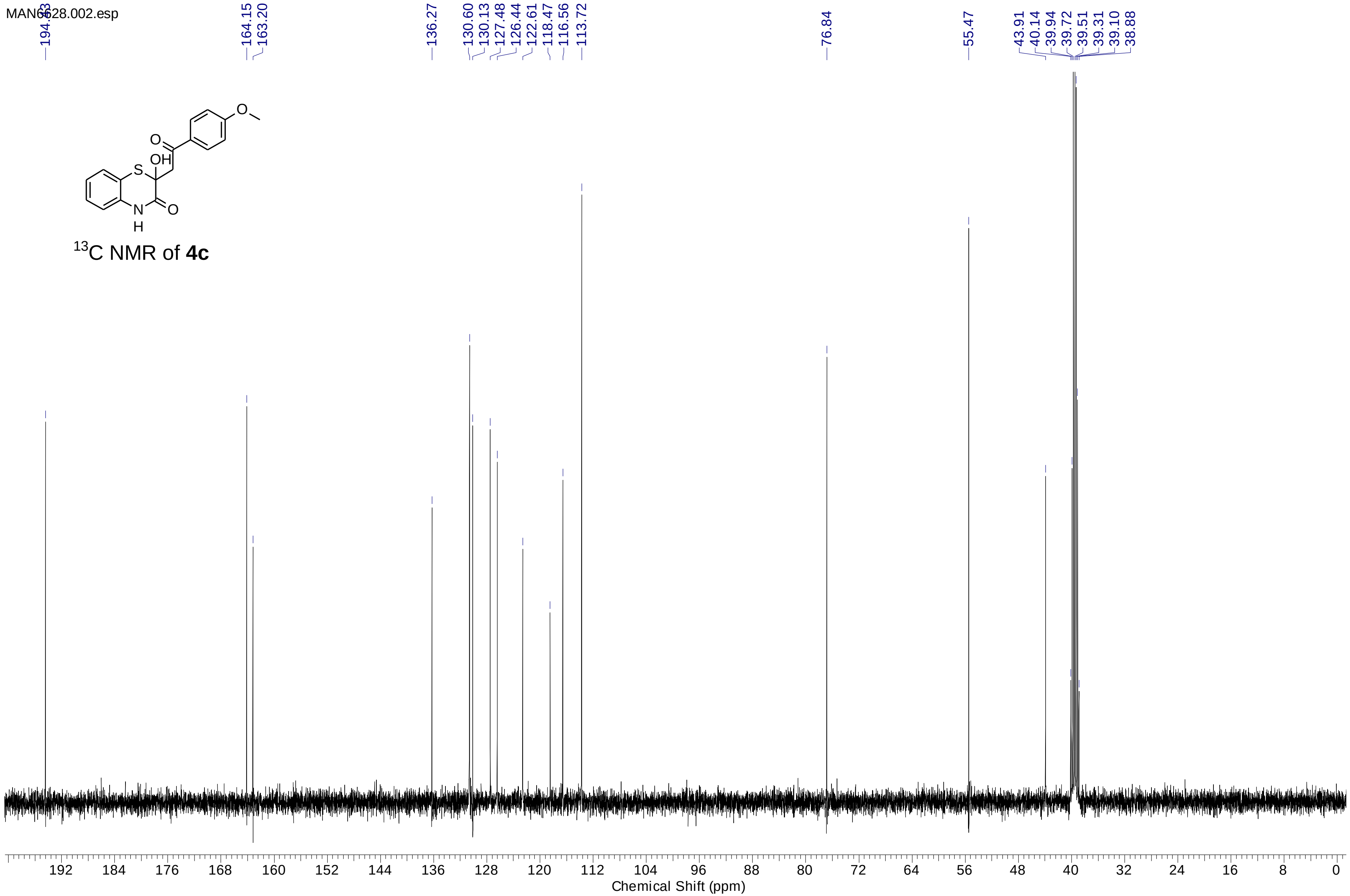
MAN6628.002.esp
194.63
164.15
163.20
136.27
130.60
130.13
127.48
126.44
122.61
118.47
116.56
113.72
76.84
55.47
43.91
40.14
39.94
39.72
39.51
39.31
39.10
38.88
S
OH
O
O
N
H
Chemical Shift (ppm)

^{13}C NMR of **4c**

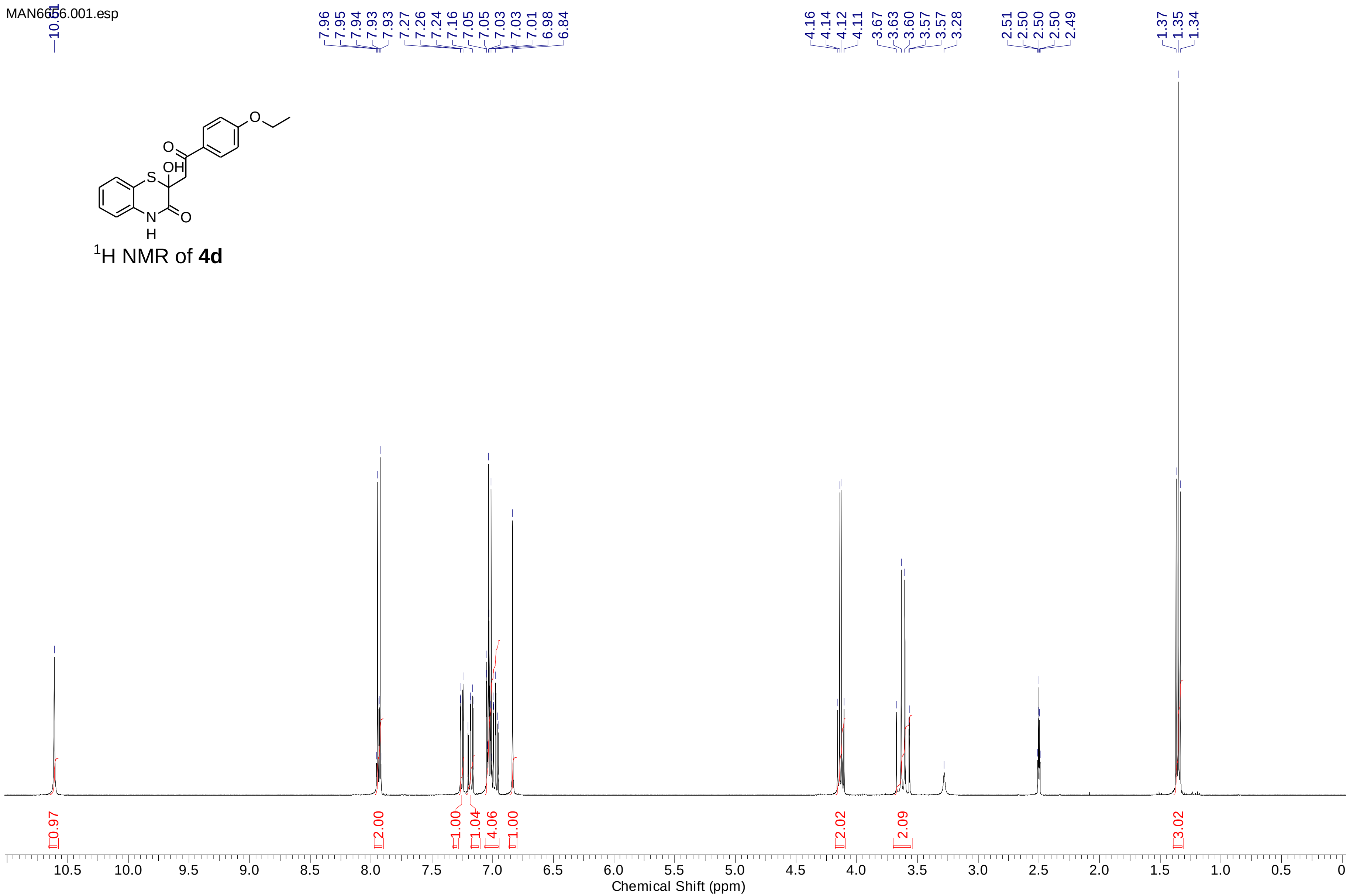

MAN6656.001.esp
10.61
7.96
7.95
7.94
7.93
7.93
7.27
7.26
7.24
7.16
7.05
7.05
7.03
7.03
7.01
6.98
6.84
4.16
4.14
4.12
4.11
3.67
3.63
3.60
3.57
3.57
3.28
2.51
2.50
2.50
2.50
2.49
1.37
1.35
1.34
O
OH
S
N
H
O
O
¹H NMR of **4d**
0.97
2.00
1.00
1.04
4.06
1.00
2.02
2.09
3.02
10.5
10.0
9.5
9.0
8.5
8.0
7.5
7.0
6.5
6.0
5.5
5.0
4.5
4.0
3.5
3.0
2.5
2.0
1.5
1.0
0.5
0
Chemical Shift (ppm)

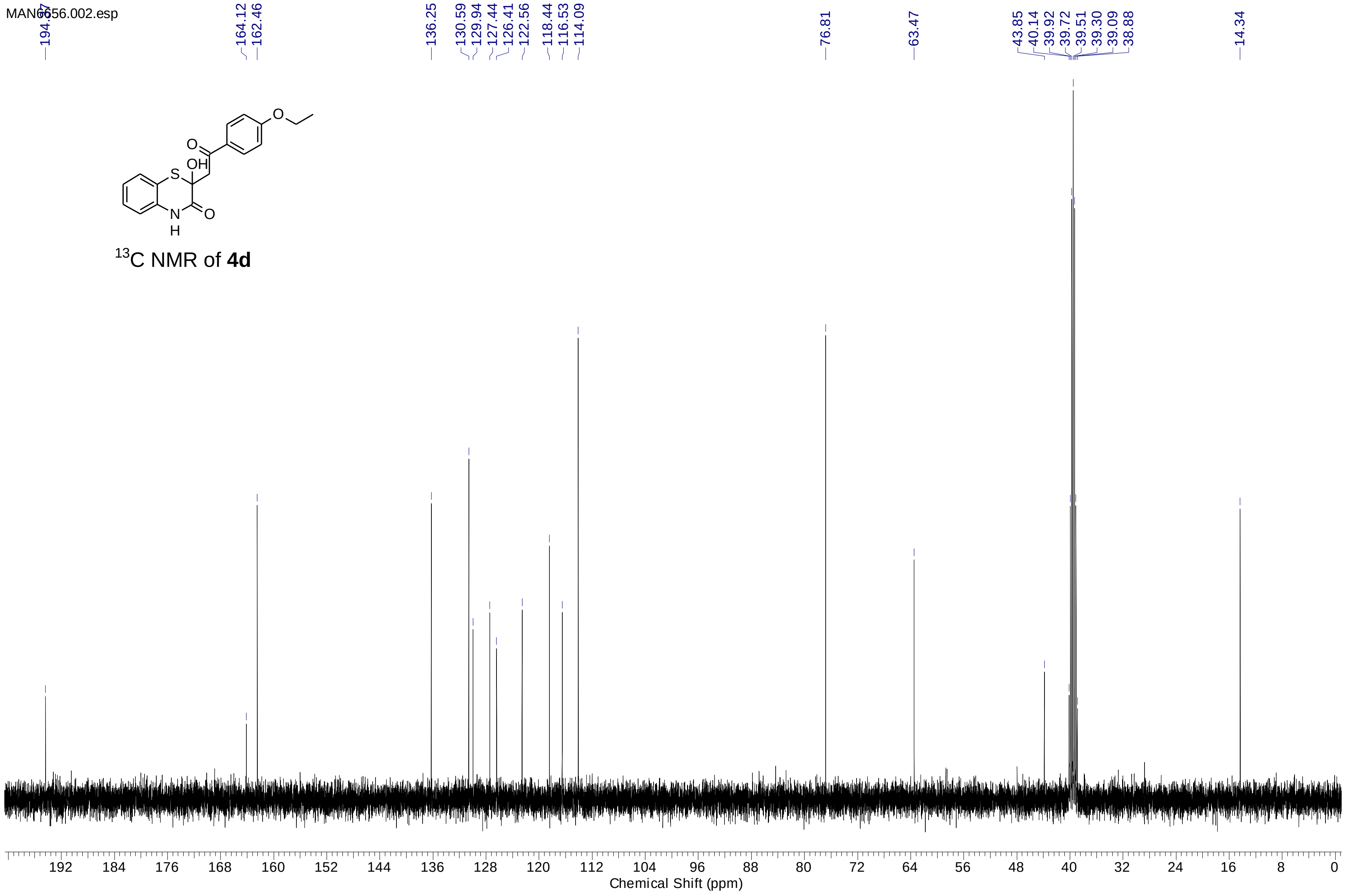
MAN6656.002.esp
194.67
164.12
162.46
136.25
130.59
129.94
127.44
126.41
122.56
118.44
116.53
114.09
76.81
63.47
43.85
40.14
39.92
39.72
39.51
39.30
39.09
38.88
14.34
O
OH
S
N
H
O
O
192 184 176 168 160 152 144 136 128 120 112 104 96 88 80 72 64 56 48 40 32 24 16 8 0
Chemical Shift (ppm)

^{13}C NMR of **4d**

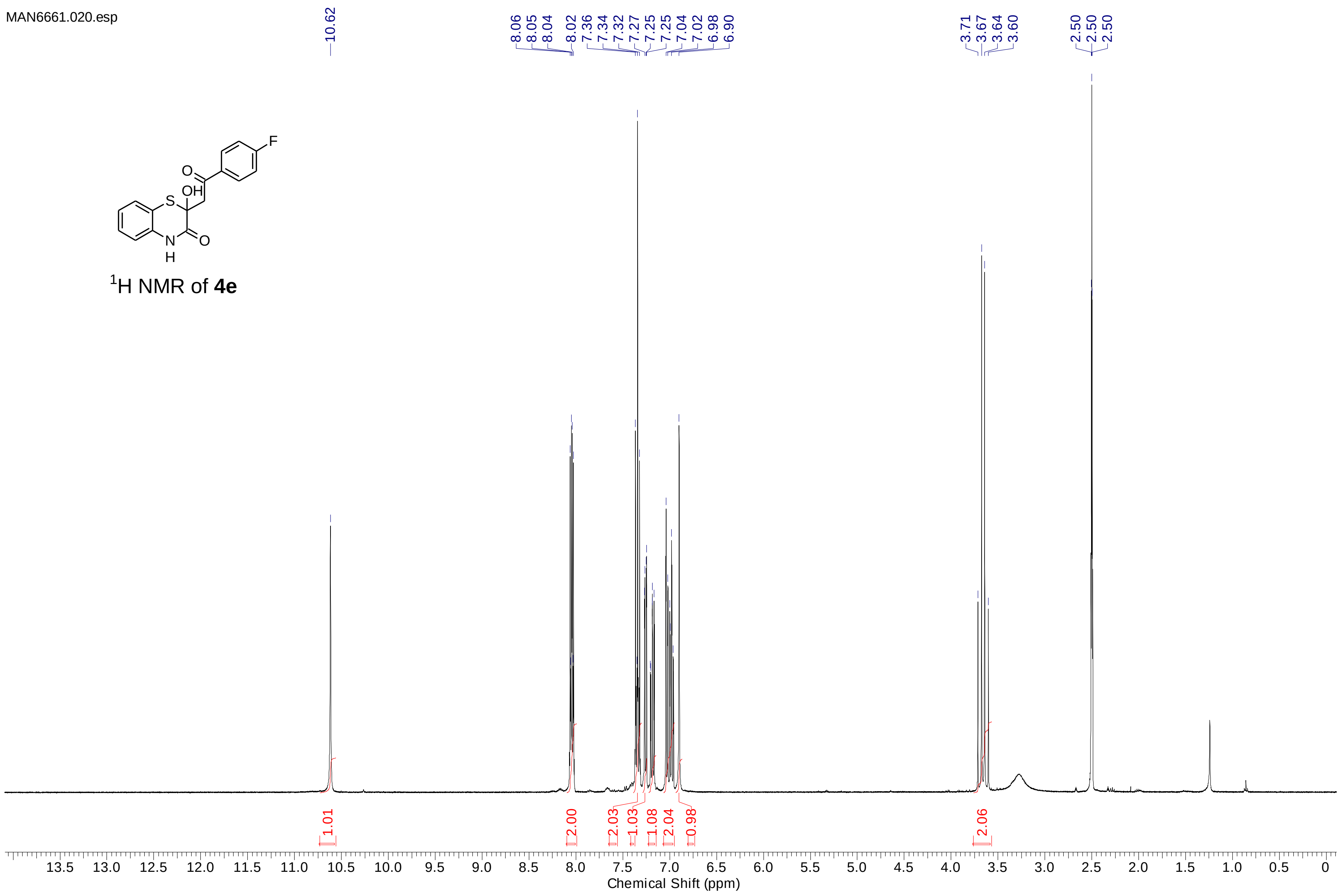
MAN6661.020.esp
10.62
8.06 8.05 8.04 8.02 7.36 7.34 7.32 7.27 7.25 7.25 7.04 7.02 6.98 6.90
3.71 3.67 3.64 3.60
2.50 2.50 2.50
O
OH
F
S
N
H
O
^{1}H NMR of **4e**
1.01
2.00
2.03
1.03
1.08
2.04
0.98
2.06
13.5 13.0 12.5 12.0 11.5 11.0 10.5 10.0 9.5 9.0 8.5 8.0 7.5 7.0 6.5 6.0 5.5 5.0 4.5 4.0 3.5 3.0 2.5 2.0 1.5 1.0 0.5 0
Chemical Shift (ppm)

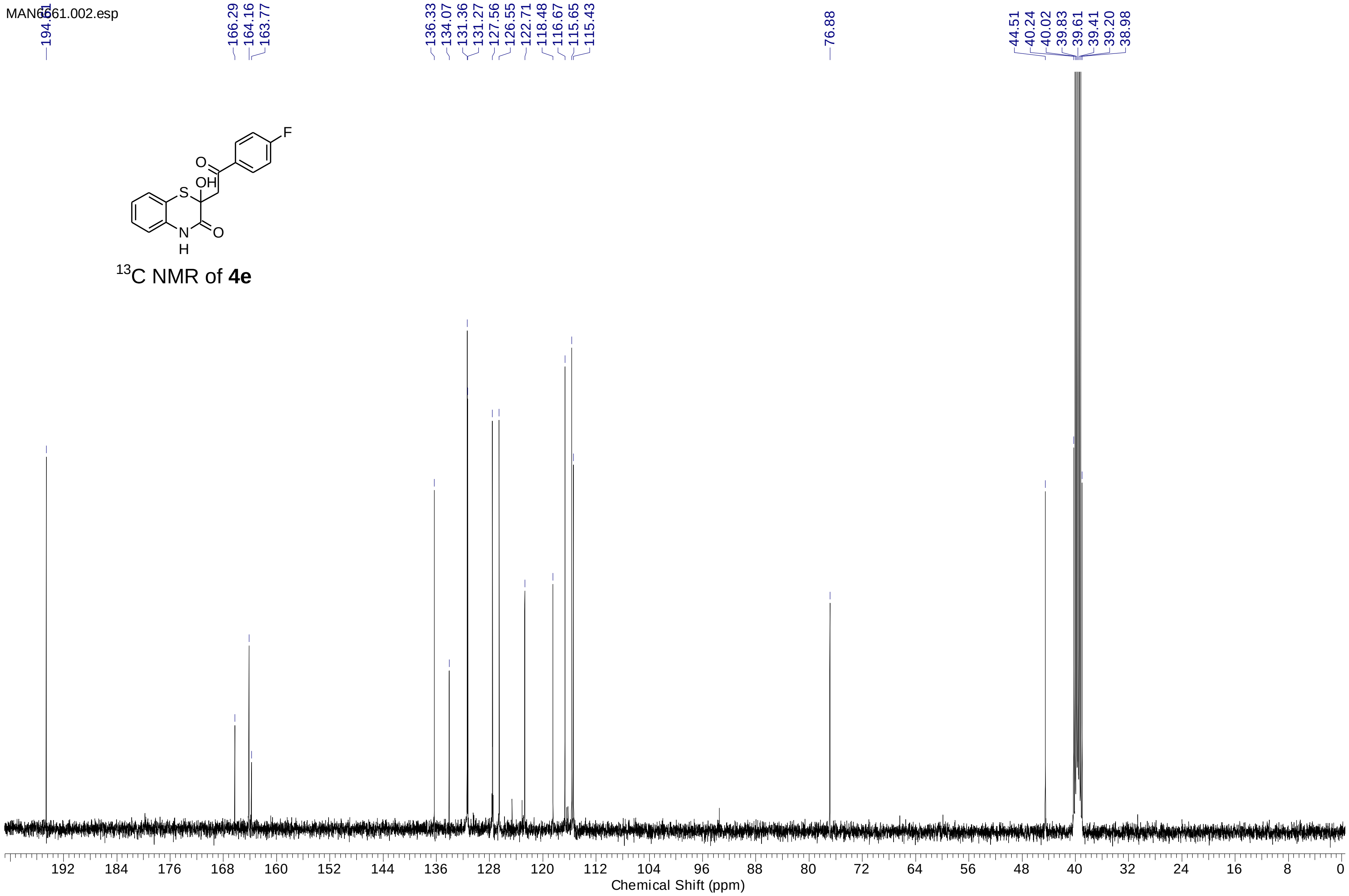
MAN6661.002.esp
194.61
166.29
164.16
163.77
136.33
134.07
131.36
131.27
127.56
126.55
122.71
118.48
116.67
115.65
115.43
76.88
44.51
40.24
40.02
39.83
39.61
39.41
39.20
38.98
O
F
OH
S
N
H
O
^{13}C NMR of **4e**
192 184 176 168 160 152 144 136 128 120 112 104 96 88 80 72 64 56 48 40 32 24 16 8 0
Chemical Shift (ppm)

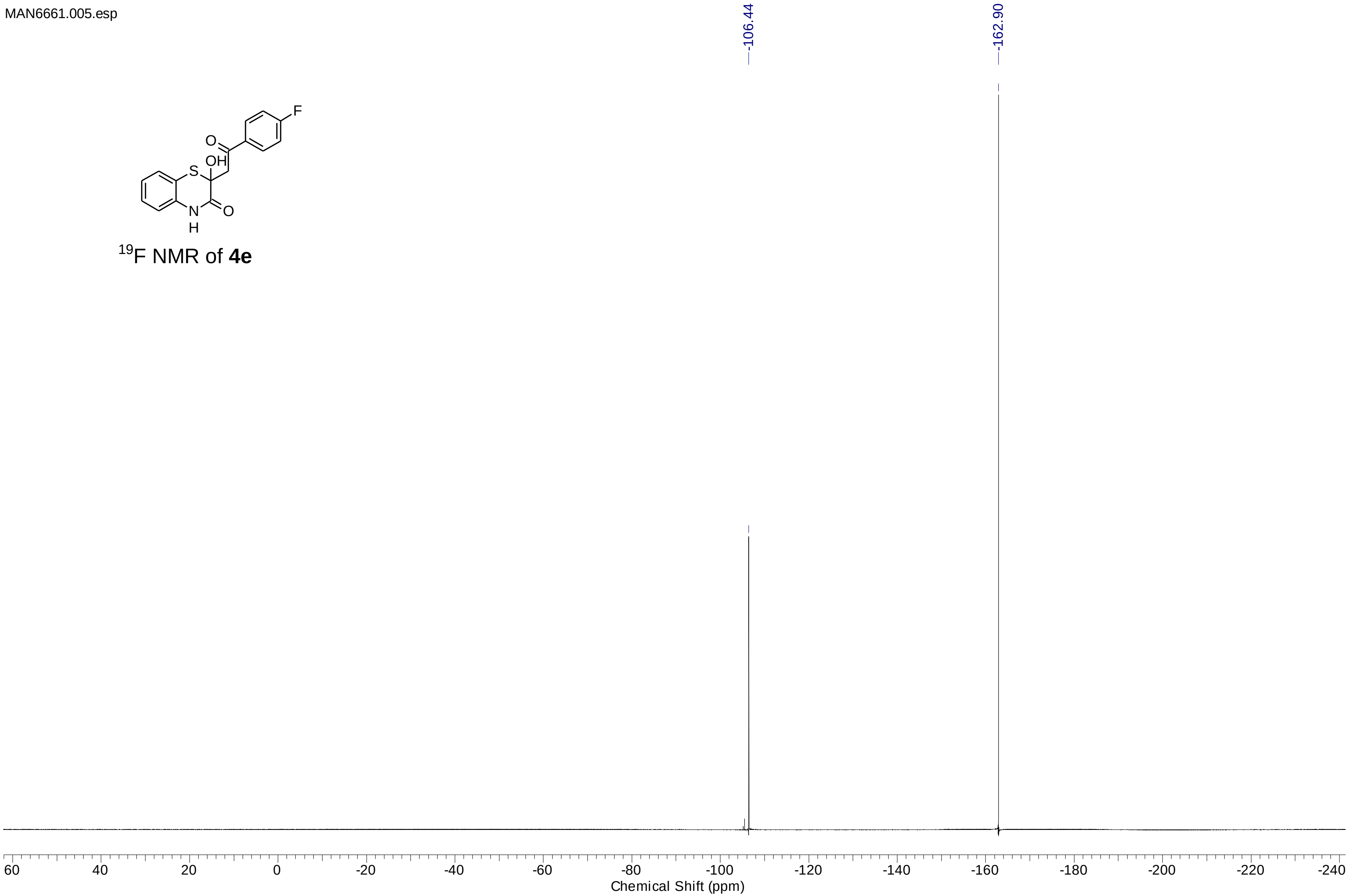
MAN6661.005.esp
-106.44
-162.90
60 40 20 0 -20 -40 -60 -80 -100 -120 -140 -160 -180 -200 -220 -240
Chemical Shift (ppm)

^{19}F NMR of **4e**

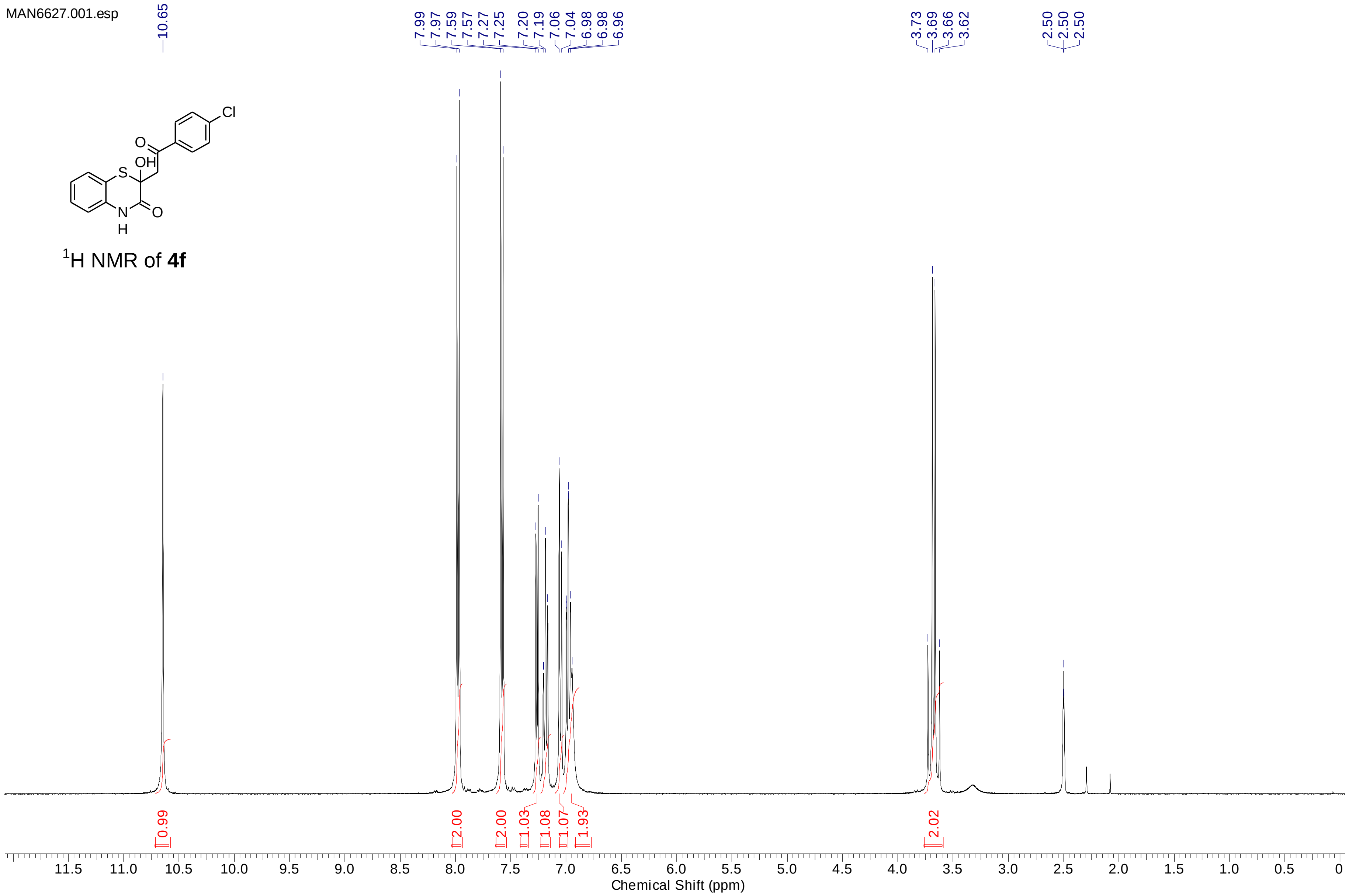
MAN6627.001.esp
10.65
7.99
7.97
7.59
7.57
7.27
7.25
7.20
7.19
7.06
7.04
6.98
6.98
6.96
3.73
3.69
3.66
3.62
2.50
2.50
2.50
Cl
O
OH
S
N
H
O
0.99
2.00
2.00
1.03
1.08
1.07
1.93
2.02
Chemical Shift (ppm)

^{1}H NMR of **4f**

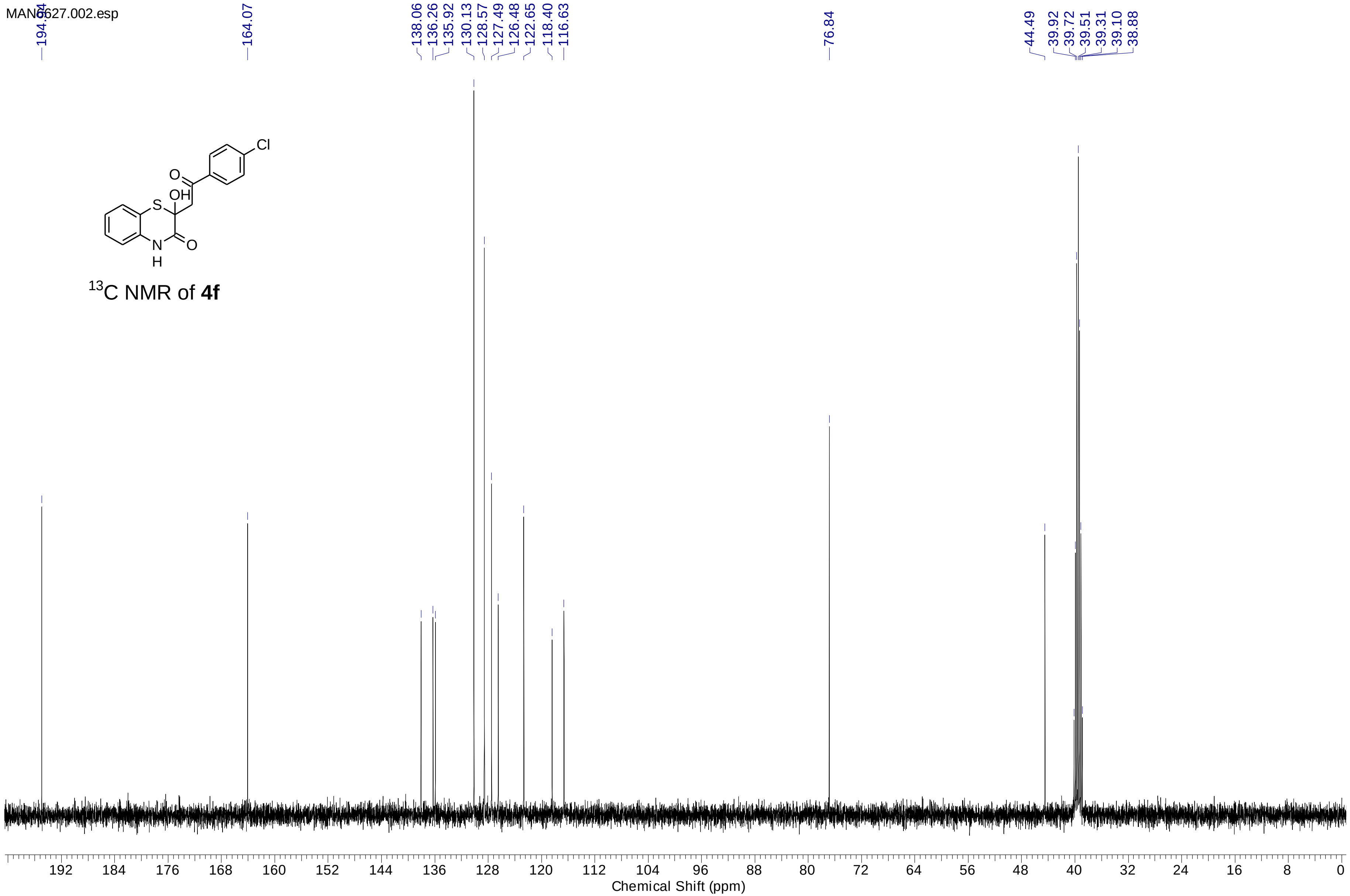
MAN6627.002.esp
194.04
164.07
138.06
136.26
135.92
130.13
128.57
127.49
126.48
122.65
118.40
116.63
76.84
44.49
39.92
39.72
39.51
39.31
39.10
38.88
O
Cl
OH
S
N
H
O
^{13}C NMR of **4f**
192
184
176
168
160
152
144
136
128
120
112
104
96
88
80
72
64
56
48
40
32
24
16
8
0
Chemical Shift (ppm)

MAN5085.001.esp

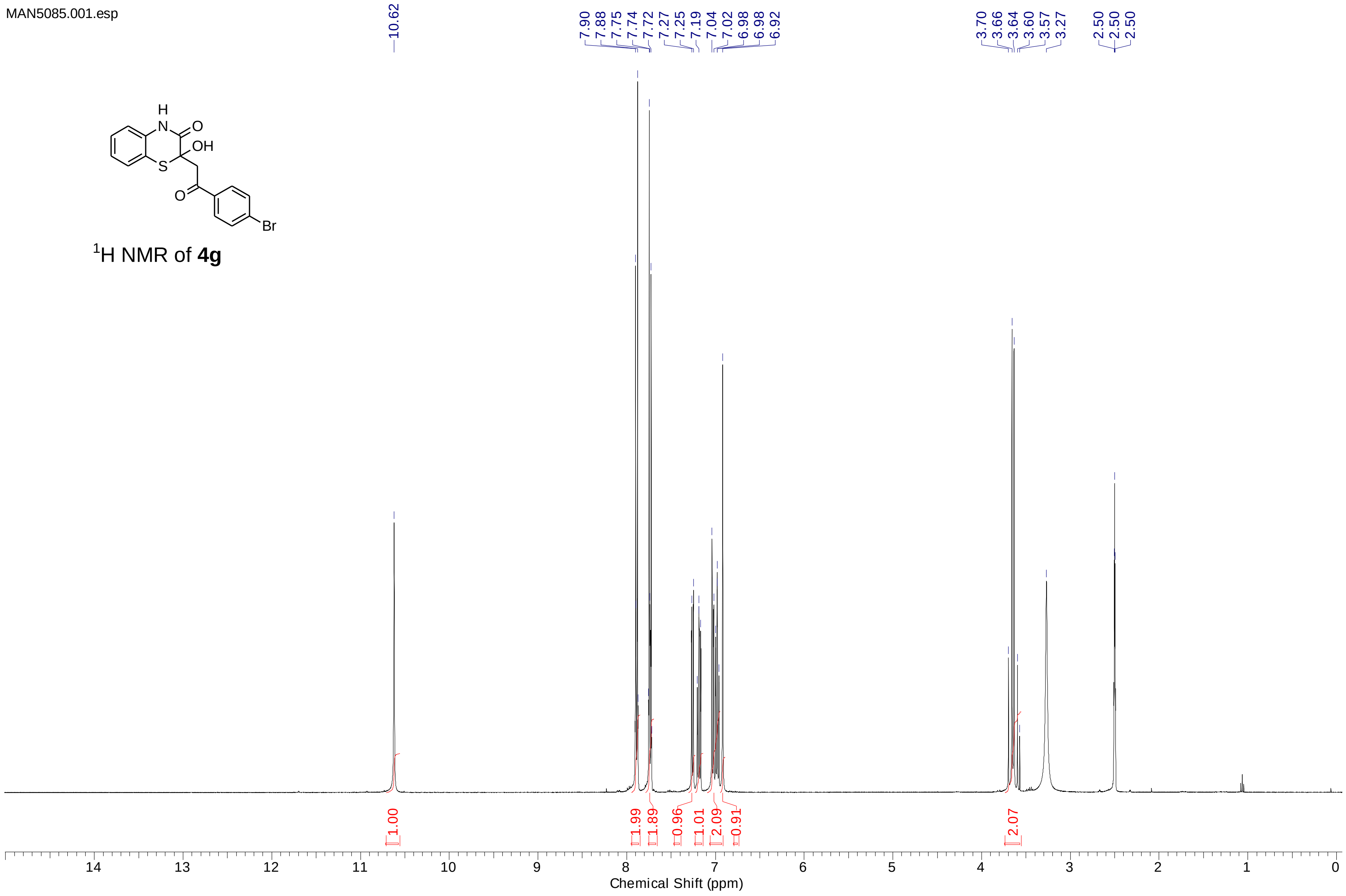

^{1}H NMR of **4g**

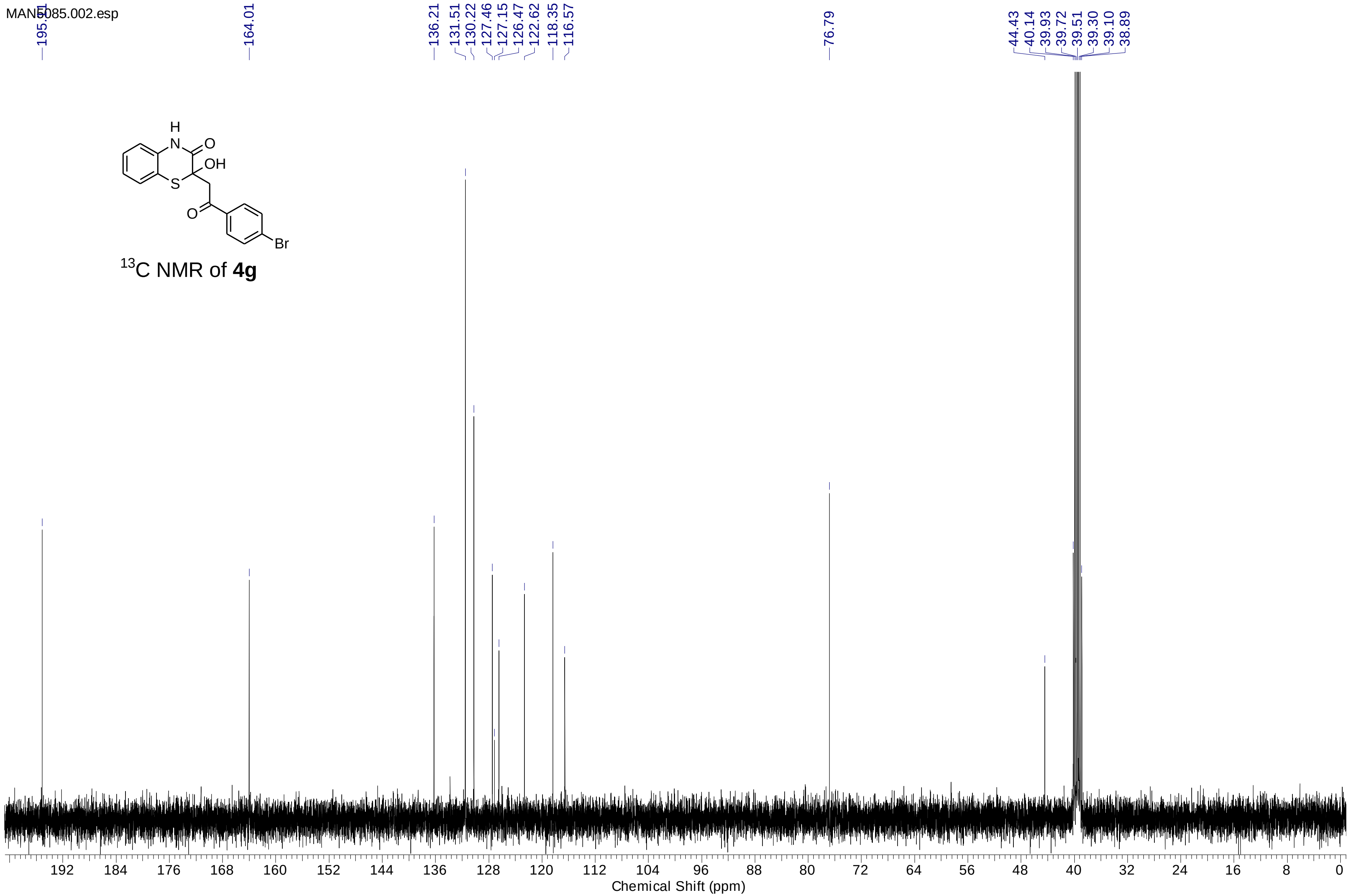
MAN5085.002.esp
195.51
164.01
136.21
131.51
130.22
127.46
127.15
126.47
122.62
118.35
116.57
76.79
44.43
40.14
39.93
39.72
39.51
39.30
39.10
38.89
H
N
O
OH
S
O
Br
^{13}C NMR of **4g**
192
184
176
168
160
152
144
136
128
120
112
104
96
88
80
72
64
56
48
40
32
24
16
8
0
Chemical Shift (ppm)

MAN6657.001.esp

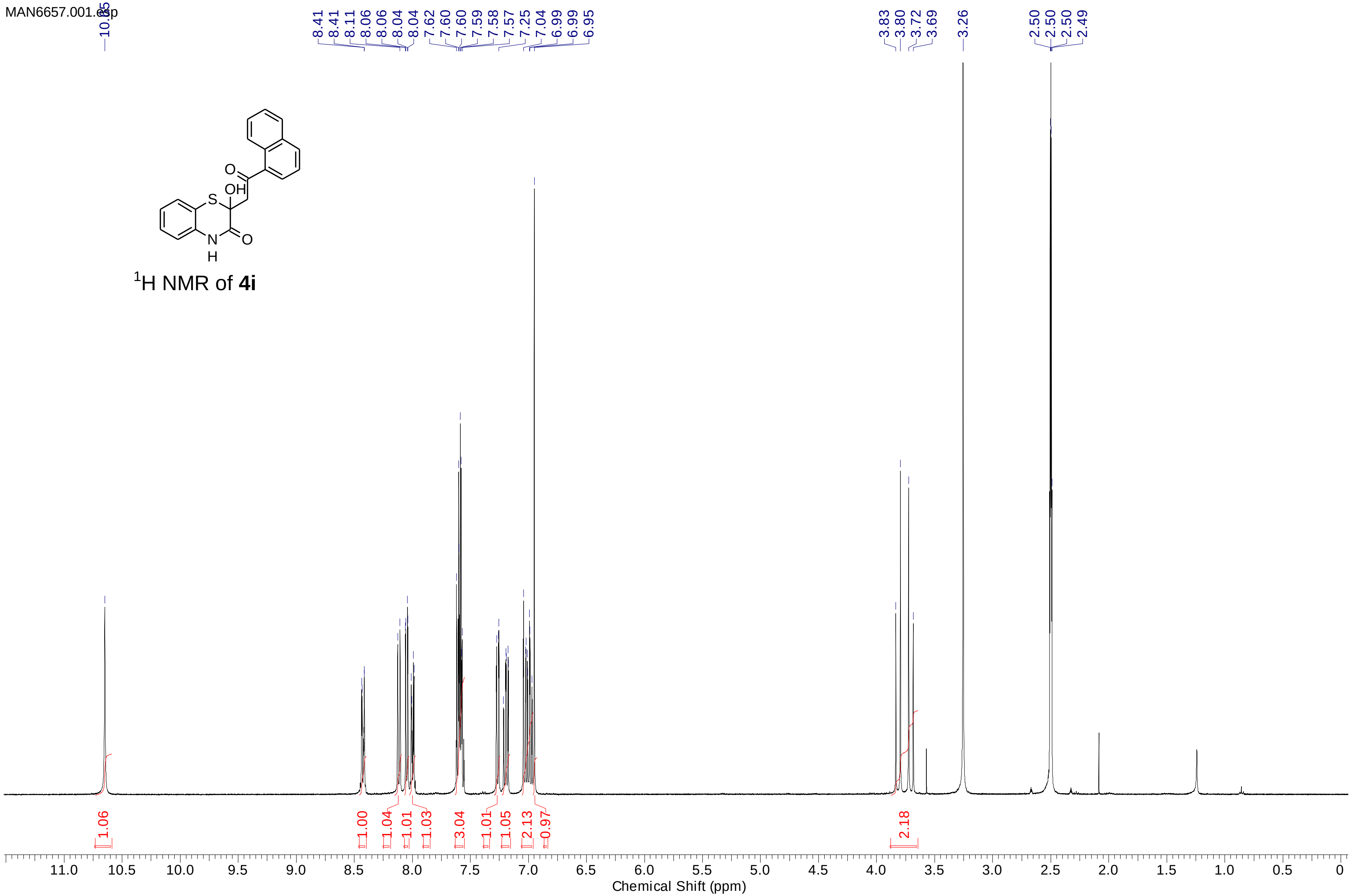

^{1}H NMR of **4i**

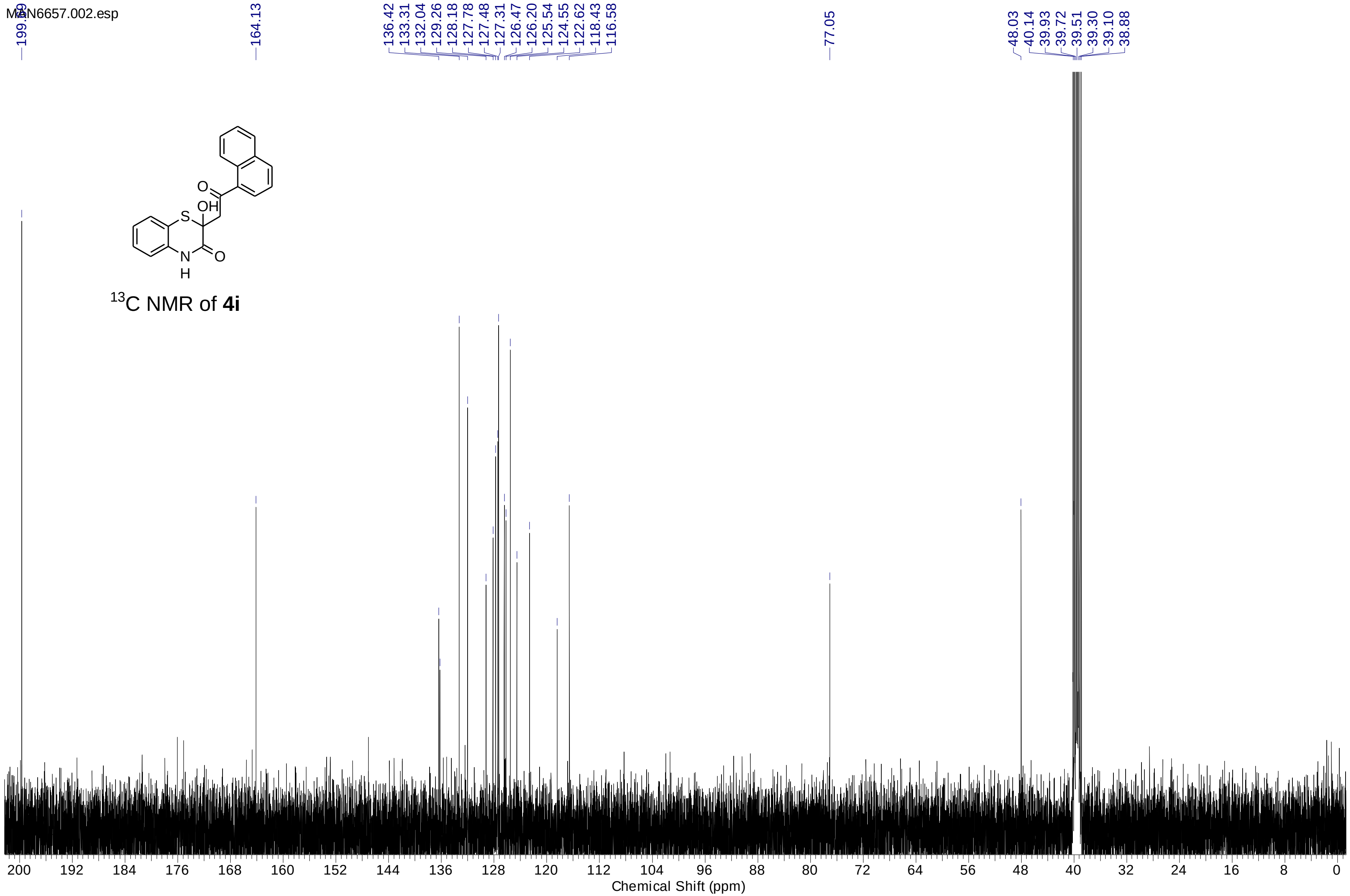
MAN6657.002.esp
199.19
164.13
136.42 133.31 132.04 129.26 128.18 127.78 127.48 127.31 126.47 126.20 125.54 124.55 122.62 118.43 116.58
77.05
48.03 40.14 39.93 39.72 39.51 39.30 39.10 38.88
O
OH
S
N
H
O
200 192 184 176 168 160 152 144 136 128 120 112 104 96 88 80 72 64 56 48 40 32 24 16 8 0
Chemical Shift (ppm)

^{13}C NMR of **4i**

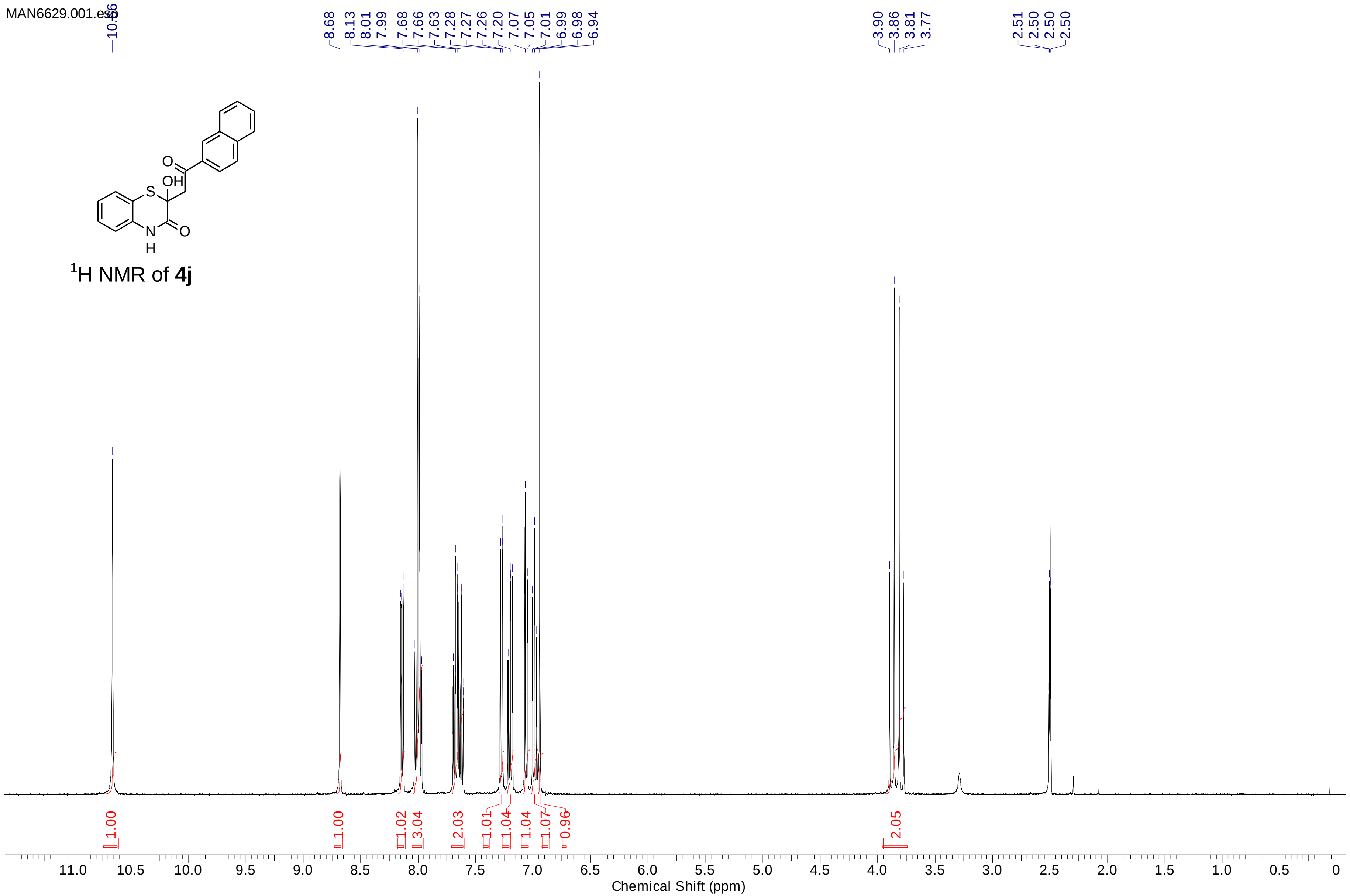
MAN6629.001.esp
10.66
8.68
8.13
8.01
7.99
7.68
7.66
7.63
7.28
7.27
7.26
7.20
7.07
7.05
7.01
6.99
6.98
6.94
3.90
3.86
3.81
3.77
2.51
2.50
2.50
2.50
O
OH
S
N
H
O
1.00
1.00
1.02
3.04
2.03
1.01
1.04
1.04
1.07
0.96
2.05
11.0 10.5 10.0 9.5 9.0 8.5 8.0 7.5 7.0 6.5 6.0 5.5 5.0 4.5 4.0 3.5 3.0 2.5 2.0 1.5 1.0 0.5 0
Chemical Shift (ppm)

^{1}H NMR of **4j**

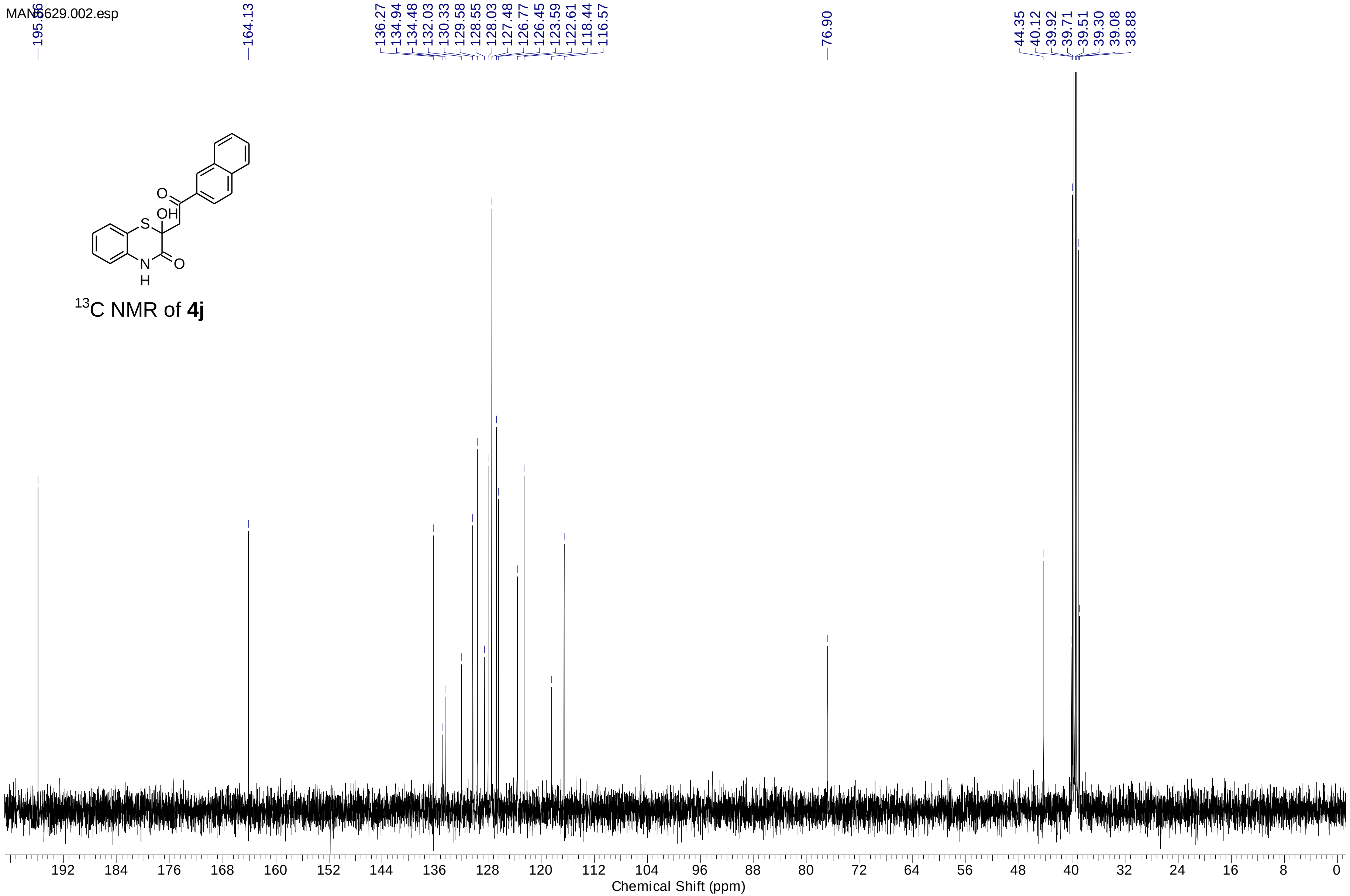
MAN6629.002.esp
195.86
164.13
136.27
134.94
134.48
132.03
130.33
129.58
128.55
128.03
127.48
126.77
126.45
123.59
122.61
118.44
116.57
76.90
44.35
40.12
39.92
39.71
39.51
39.30
39.08
38.88
O
OH
S
N
H
O
^{13}C NMR of **4j**
192
184
176
168
160
152
144
136
128
120
112
104
96
88
80
72
64
56
48
40
32
24
16
8
0
Chemical Shift (ppm)

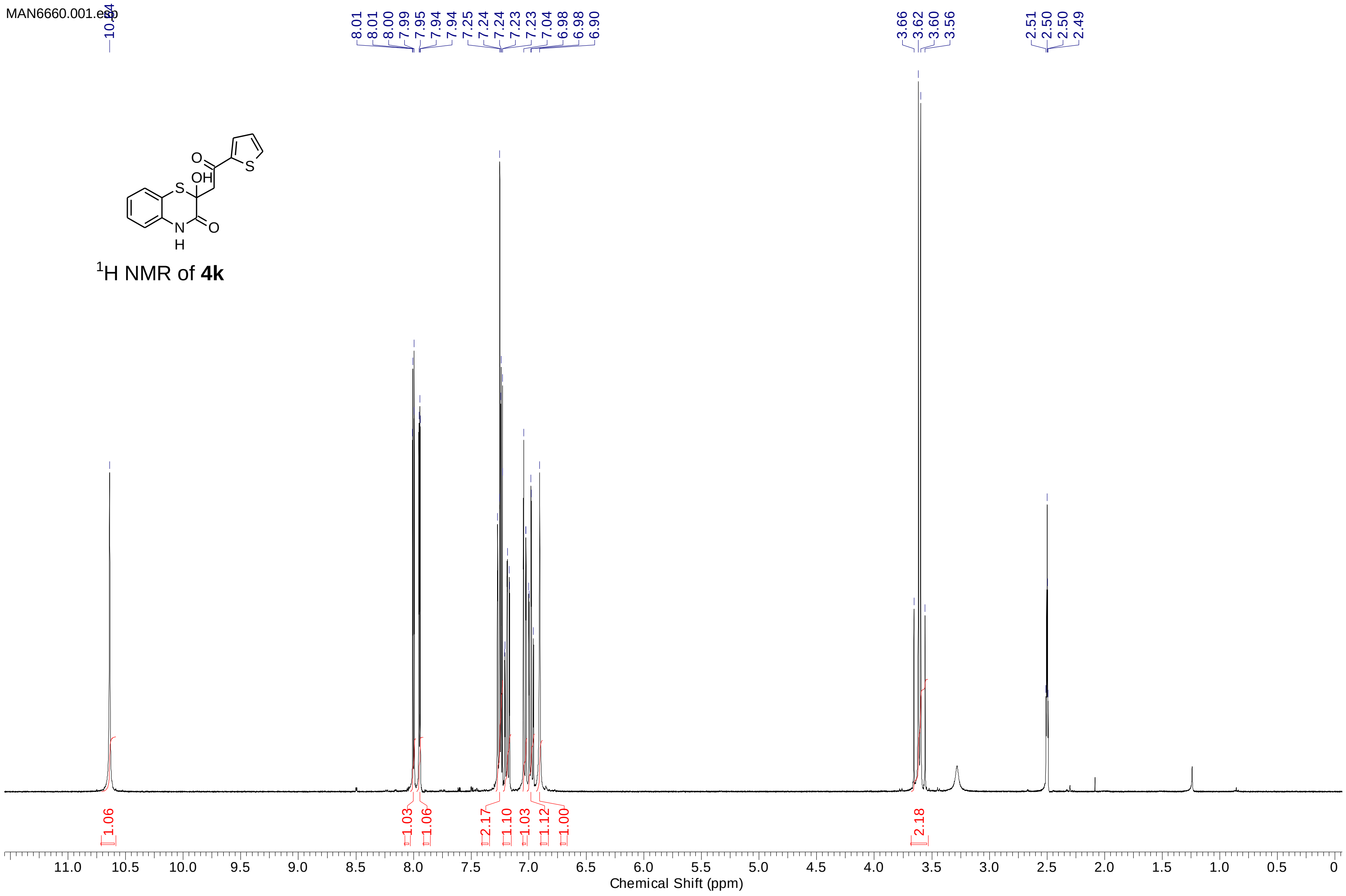
MAN6660.001.esp
10.64
8.01
8.01
8.00
7.99
7.95
7.94
7.94
7.25
7.24
7.24
7.23
7.23
7.04
6.98
6.98
6.90
3.66
3.62
3.60
3.56
2.51
2.50
2.50
2.49
1.06
1.03
1.06
2.17
1.10
1.03
1.12
1.00
2.18
11.0 10.5 10.0 9.5 9.0 8.5 8.0 7.5 7.0 6.5 6.0 5.5 5.0 4.5 4.0 3.5 3.0 2.5 2.0 1.5 1.0 0.5 0
Chemical Shift (ppm)
O
OH
S
S
N
H
O

^1H NMR of **4k**

MAN6660.002.esp

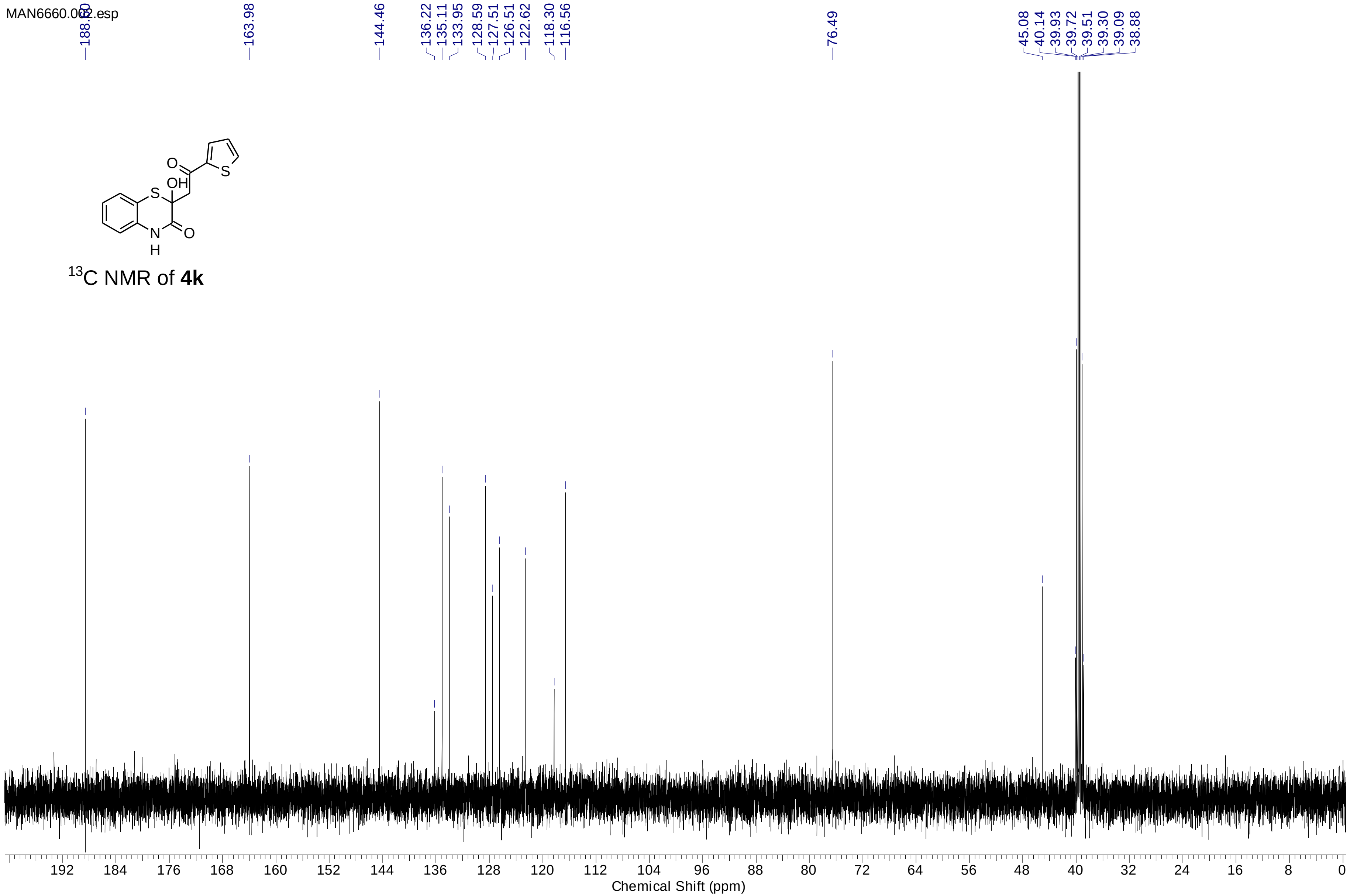

^{13}C NMR of **4k**

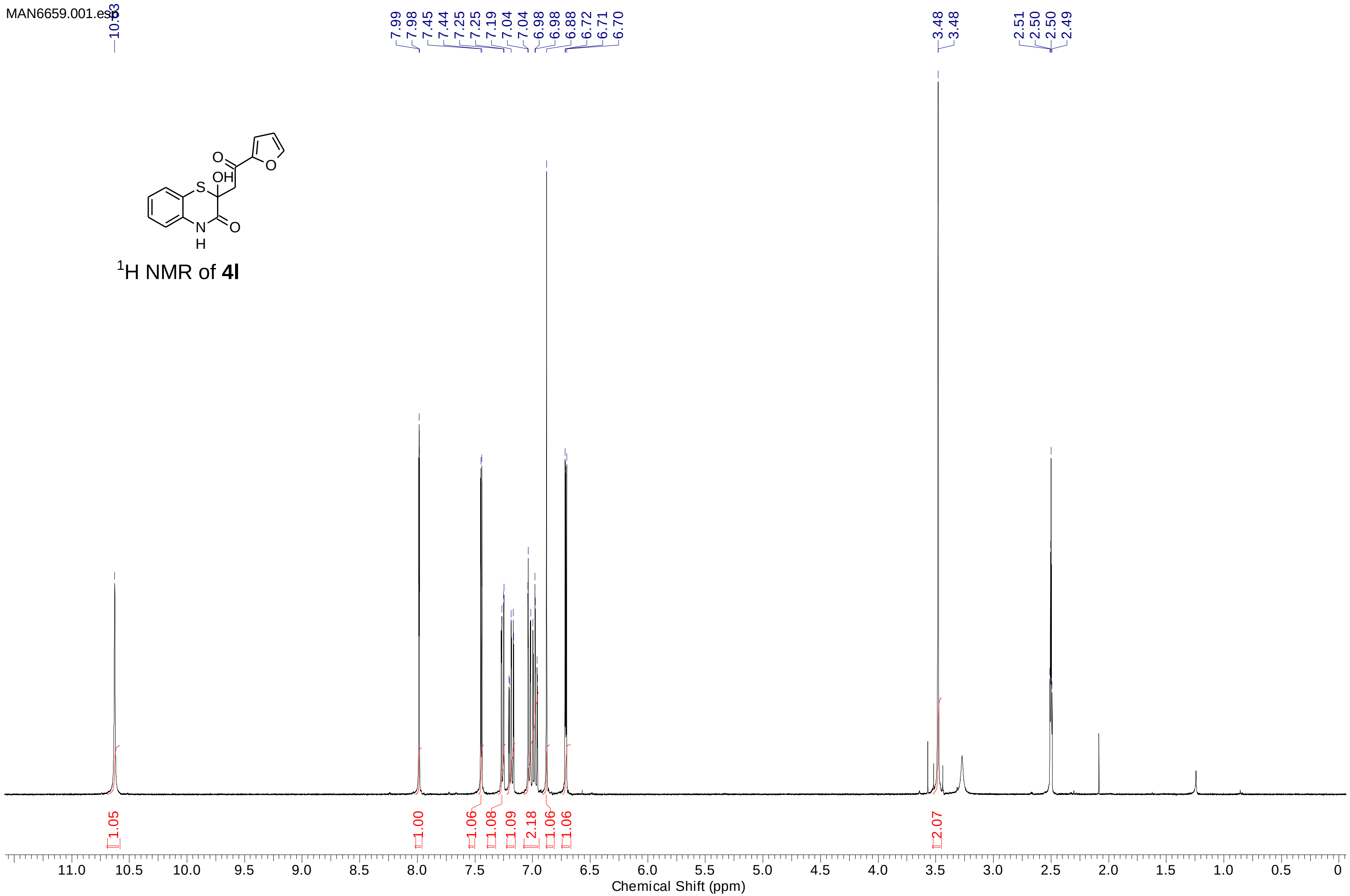
MAN6659.001.esp
10.63
7.99 7.98 7.45 7.44 7.25 7.25 7.19 7.04 7.04 6.98 6.98 6.88 6.72 6.71 6.70
3.48 3.48
2.51 2.50 2.50 2.49
O
OH
S
N
H
O
O
1.05
1.00
1.06
1.08
1.09
2.18
1.06
1.06
2.07
11.0 10.5 10.0 9.5 9.0 8.5 8.0 7.5 7.0 6.5 6.0 5.5 5.0 4.5 4.0 3.5 3.0 2.5 2.0 1.5 1.0 0.5 0
Chemical Shift (ppm)

^{1}H NMR of **4l**

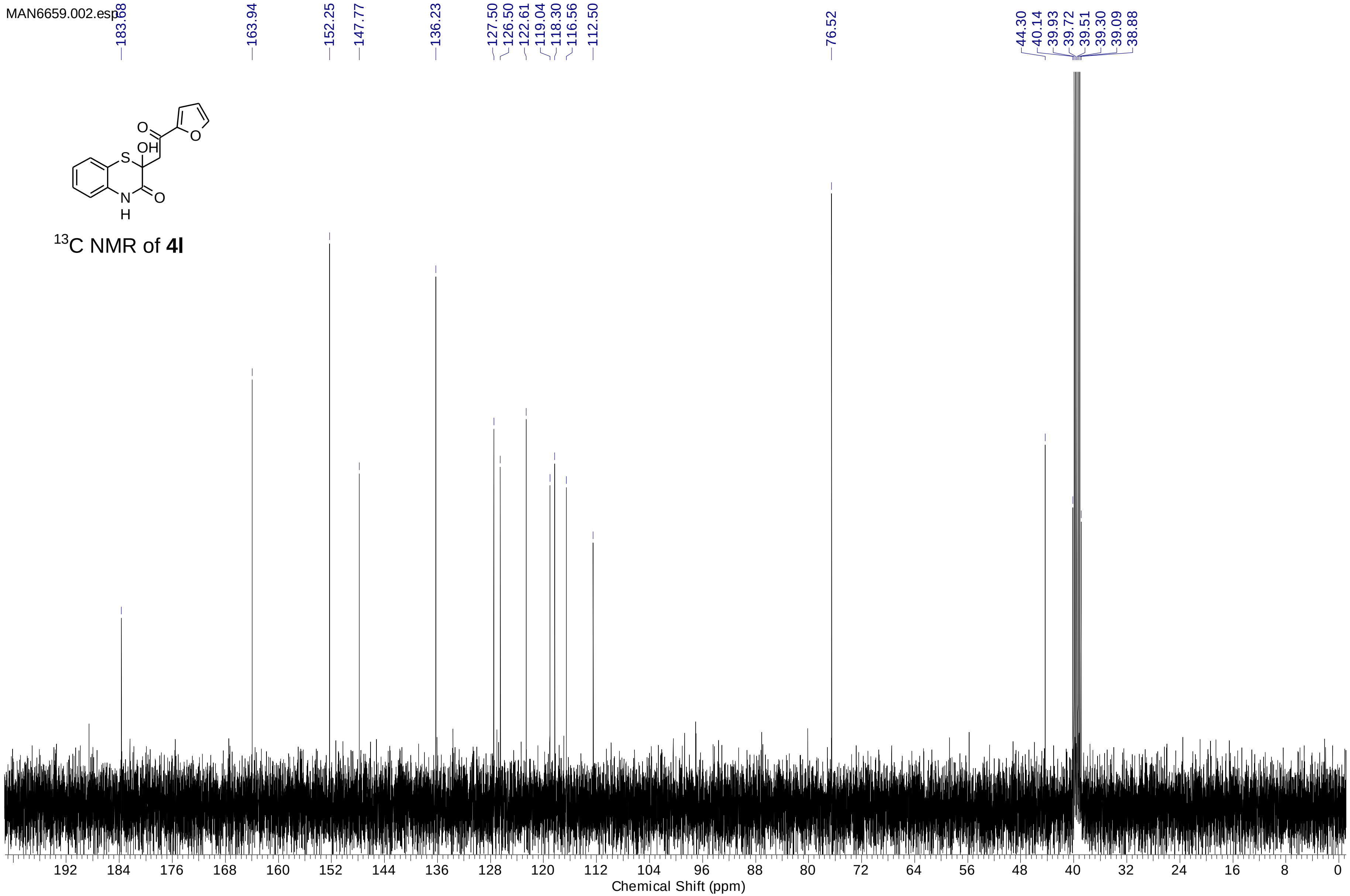
MAN6659.002.esp
183.68
163.94
152.25
147.77
136.23
127.50
126.50
122.61
119.04
118.30
116.56
112.50
76.52
44.30
40.14
39.93
39.72
39.51
39.30
39.09
38.88
Chemical Shift (ppm)

^{13}C NMR of **4l**

1-(5-Chloro-1,3-benzothiazol-2-yl)-3-hydroxy-3-phenylprop-2-en-1-one (1-(5-chloro-1,3-benzothiazol-2-yl)-3-phenylpropane-1,3-dione) (6)

was synthesized and isolated according to general procedure to compounds **4**.

Yield: 214 mg (68%); yellow; mp 150–152 °C.
^{1}H NMR (400 MHz, DMSO-d_6): δ = 8.31 (m, 2 H), 8.09 (m, 2 H), 7.69 (m, 2 H), 7.61 (m, 2 H), 7.42 (s, 1 H), 5.03 (s, CH_2 in diketoform) ppm.
^{13}C NMR (100 MHz, DMSO-d_6): δ = 182.8, 179.5, 167.4, 153.8, 134.9, 133.6, 133.0, 132.1, 129.1 (2 C), 127.6, 127.2 (2 C), 124.6, 123.7, 93.4 ppm.
IR (mineral oil): 3172, 1606 cm^{-1}.
MS (ESI+): m/z calcd for $C_{16}H_{10}ClNO_2S$ +H^+: 316.02 [M+H^+]; found: 316.01.
Anal. Calcd (%) for $C_{16}H_{10}ClNO_2S$: C 60.86; H 3.19; N 4.44. Found: C 61.11; H 3.25; N 4.40.
λ_{max} = 376 nm.

MAN6658.001.esp

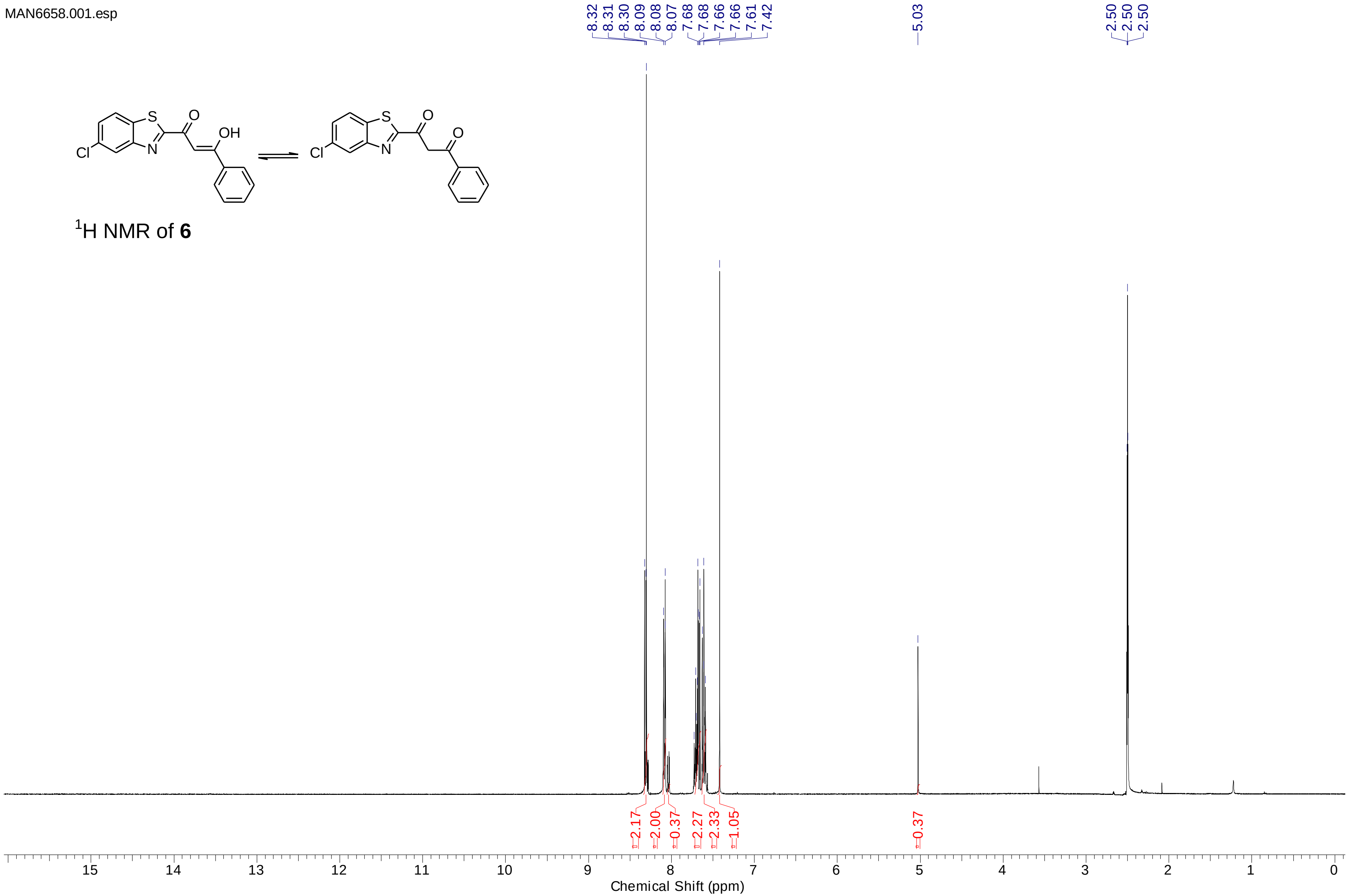

^{1}H NMR of **6**

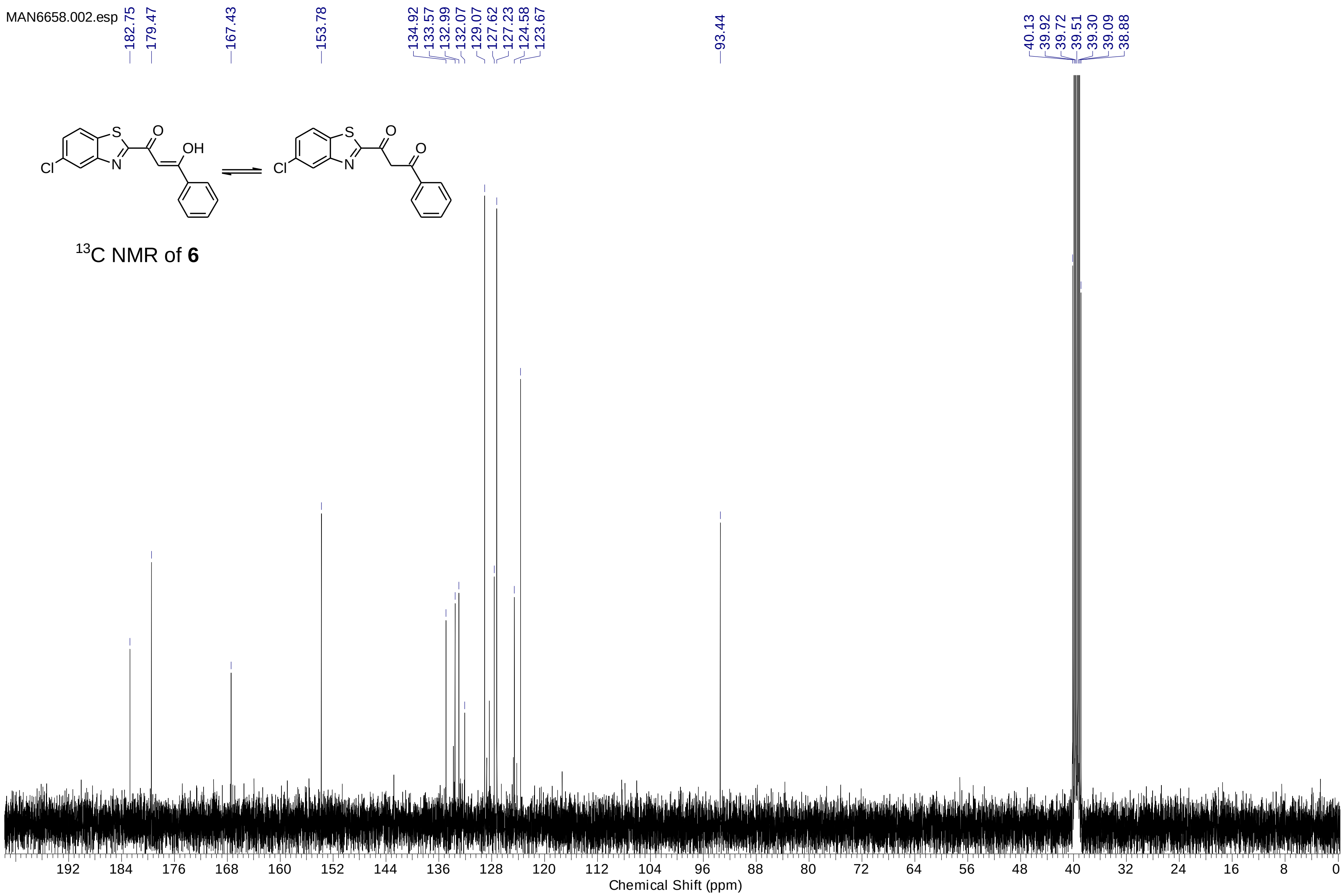
MAN6658.002.esp
182.75
179.47
167.43
153.78
134.92
133.57
132.99
132.07
129.07
127.62
127.23
124.58
123.67
93.44
40.13
39.92
39.72
39.51
39.30
39.09
38.88
Cl
S
N
O
OH
192 184 176 168 160 152 144 136 128 120 112 104 96 88 80 72 64 56 48 40 32 24 16 8 0
Chemical Shift (ppm)

^{13}C NMR of **6**

Antimicrobial assay by CO-ADD (ESKAPE pathogens) [4]

Table 1. Results of antimicrobial assay

CompoundID	Compound	ProjectID	RunID	Sel	Act	Sa	Ec	Kp	Pa	Ab	Ca	Cn	Conc
C0104064	**3a**	P0795	PSR00168	0	0	22,16	5,73	12,69	9,42	13,3	10,49	-1,77	32 ug/mL
C0104065	**3b**	P0795	PSR00168	0	0	21,04	4,16	5,51	10,86	12,34	7,25	-2,31	32 ug/mL
C0104043	**3m**	P0795	PSR00168	0	0	12,34	3,88	2,09	11,23	9,56	10,56	-5,07	32 ug/mL
C0103901	**3g**	P0795	PSR00168	0	0	25,88	-1,01	18,25	10,14	4,11	4,2	3,07	32 ug/mL
C0103900	**3f**	P0795	PSR00168	0	0	23,93	-0,67	8	10,75	3,94	3,42	4,6	32 ug/mL
C0103902	**3c**	P0795	PSR00168	0	0	22	0,04	3,13	7,93	6,66	3,27	1,54	32 ug/mL
C0103954	**3j**	P0795	PSR00168	0	0	27,37	1,81	4,94	7,3	18,98	1,31	5,63	32 ug/mL

Table 2. Tested microorganisms' strains

Abbreviation	Code	Name	Description	Strain	Organsim	Type
Sa	GP_020	*Staphylococcus aureus*	MRSA	ATCC 43300	Bacteria	G+ve
Ec	GN_001	*Escherichia coli*	FDA control	ATCC 25922	Bacteria	G-ve
Kp	GN_003	*Klebsiella pneumoniae*	MDR	ATCC 700603	Bacteria	G-ve
Ab	GN_034	*Acinetobacter baumannii*	Type strain	ATCC 19606	Bacteria	G-ve
Pa	GN_042	*Pseudomonas aeruginosa*	Type strain	ATCC 27853	Bacteria	G-ve
Ca	FG_001	*Candida albicans*	CLSI reference	ATCC 90028	Fungi	Yeast
Cn	FG_002	*Cryptococcus neoformans* var. *grubii*	Type strain	H99; ATCC 208821	Fungi	Yeast

Antibacterial data collection

Inhibition of bacterial growth was determined measuring the absorbance at 600 nm (OD_{600}), using a Tecan M1000 Pro monochromator plate reader. The percentage of growth inhibition was calculated for each well, using the negative control (media only) and positive control (bacteria without inhibitors) on the same plate as references.

Antifungal data collection

The growth inhibition of *C. albicans* was determined measuring absorbance at 530 nm (OD_{530}), while the growth inhibition of *C. neoformans* was determined measuring the difference in absorbance between 600 and 570 nm ($OD_{600-570}$), after the addition of resazurin (0.001% final concentration) and incubation at 35 °C for additional 2 h. The absorbance was measured using a Biotek Synergy HTX plate reader. The percentage of growth inhibition was calculated for each well, using the negative control (media only) and positive control (bacteria without inhibitors) on the same plate as references.

Inhibition (Table 1)

Percentage growth inhibition of an individual sample is calculated based on negative controls (media only) and positive controls (bacterial/fungal media without inhibitors). Please note negative inhibition values indicate that the growth rate (or OD600) is higher compared to the negative control (Bacteria/fungi only, set to 0% inhibition). The growth rates for all bacteria and fungi has a variation of -/+ 10%, which is within the reported normal distribution of bacterial/fungal growth. Any significant variation (or outliers/hits) is identified by the modified Z-Score, and actives are selected by a combination of inhibition value and Z-Score.

Z-Score

Z-Score analysis is done to investigate outliers or hits among the samples. The Z-Score is calculated based on the sample population using a modified Z-Score method which accounts for possible skewed sample population. The modified method uses median and MAD (median average derviation) instead of average and sd, and a scaling factor [Iglewicz, B. & Hoaglin, D. C. Volume 16: How to Detect and Handle Outliers. The ASQC Basic Reference in Quality Control: Statistical Techniques, 1993]: M(i) = 0.6745 * (x(i) - median(x))/MAD). M(i) values of > |2.5| (absolute) label outliers or hits.

Quality Control

All screening is performed as two replica (n = 2), with both replicas on different assay plates, but from single plating and performed in a single screening experiment (microbial incubation). Each individual value is reported in the table (see ..1 and ..2). In addition, two values are used as quality controls for individual plates: Z'-Factor [1 - (3 * (sd(NegCtrl)+sd(PosCtrl))/(average(PosCtrl)-average(NegCtrl)))] and standard antibiotic controls at different concentrations (>MIC and <MIC). The plate passes the quality control if Z'-Factor >0.4 and Standards are active and inactive at highest and lowest concentrations, respectively. Data not supplied.

Selection of Actives

A - [Active] Samples with inhibition values equal to or above 80% and abs(Z-Score) above |2.5| for either replicate (n=2 on different plates) were classed as active.

P - [Partial Active] compounds with inhibition values between 50.9% – 79.9% or abs(Z-Sore) below |2.5|.

I - Inactive compounds with inhibition values below 50% and/or abs(Z-Sore) below |2.5|.

Act_XX

Act_XX: Indicates if a compound is active in any of the assays against a specific organism (Sa, Ec, Kp, Pa, Kp, Ca or Cn), or organism classses (GN: Gram-negative, GP: Gram-positive). Please note that the flag indicates single activities even if the average Inhibition values suggests otherwise, in which case a manual adjustment of the flag might be appropiate.

Act

Act: Indicates the number of organism-classes (GN,GP and FG) the compound has been found active against, 0 = no activity.

Sel

Sel: Indicates compounds that have been selected for further dose response studies, Hit-Confirmation. The selection includes all active as well as compounds with ambiguous results requiring confirmation of activity or inactivity.

Antitubercular assays

1. **Bactericidal activity by BACTEC**

Thw bactericidal activity of chemical compounds against Mtb (H37Rv) was studied by a standard BACTEC MGIT 960 radiometric growth system (Becton Dickinson).

Initial stock solutions of test compounds (10 mg/mL) were prepared in DMSO. Then they were diluted with Middlebrook 7H9 sterile nutrient broth (9 mL) to result in the concentration of test compounds of 1000 μg/mL. The aliquots of obtained solutions were treated by two-fold serial dilutions and added to MGIT tubes in quantities that provide the final concentrations (μg/mL): 20.0, 10.0, 5.0, 2.5, 1.25, 0.6, 0.31. Each MGIT tube contained 7 mL of Middlebrook 7H9 sterile nutrient broth. Then 0.8 mL of BACTEC MGIT OADC (oleic acid, albumin, dextrose, and catalase) growth supplement was added to each tube. In addition to the liquid medium, the tubes contained an oxygen-free fluorochrome, tris(4,7-diphenyl-1,10-phenanthroline)ruthenium chloride pentahydrate [Ru(dpp)], placed on the bottom of the tube and coated with silicone.

A 1.0 McFarland suspension of Mtb (5×10^8 microbial cells per 1 mL) was prepared using a densitometer. Next, a solution was prepared by diluting the initial suspension 10 times with sterile physiological saline solution, thus resulting in 5×10^7 microbial cells per 1 mL. Then a suspension of Mtb (0.5 mL) was added to each of the above prepared MGIT tubes. In parallel, the inoculum of Mtb was loaded into control MGIT tubes with Middlebrook 7H9 broth (0.5 mL) each containing no test compounds. For control, similar experiments were carried out with isoniazid (isonicotinic acid hydrazide, 99%, Sigma-Aldrich).

All tubes were incubated at 37 °C and analyzed by BACTEC MGIT 960. If the compound is active against Mtb, it inhibits its growth and suppresses fluorescence of Ru(dpp), while in the control tube growth is not inhibited and, accordingly, the level of fluorescence in this tube is pronounced. During bacterial growth, free oxygen is consumed inside the tubes and replaced with CO_2. As free oxygen is consumed, inhibition of the fluorochrome, Ru(dpp), is stopped. Fluorescence becomes detectable when the test tube is irradiated with UV light and is automatically registered by photosensors of BACTEC MGIT 960. The minimum dilution of the examined compound, in which the growth was not registered by BACTEC, was taken as MBC. All assays were carried out in duplicate.

The results are given in Table 3.

Table 3. Results of anti-TB assay by BACTEC.

Entry	Compound	MBC (µg/mL)[a]
1	**3a**	–
2	**3m**	–
3	Isoniazid	1.25

[a] The lowest dilution of the examined compound, in which no bacterial growth was detected on the 41th day of the assay.

2. Tuberculostatic activity by REMA [5,6]

A 1.0 McFarland suspension of Mtb (H37Rv) was prepared and 0.1 mL added to each of the following bottles: a direct control (bottle containing diluent, DMSO, but no test compound), a control containing a 1:100 organism dilution (also without test compound), and each concentration with the test compound.

From a culture of Mtb, which was in a log phase of growth on the Löwenstein–Jensen nutrient medium, a 1.0 McFarland suspension was prepared with sterile physiological saline solution and 0.2 % solution of Tween 80. The resulting suspension (50 µL) was transferred into a tube with 7H9 broth and OADC additive to give Mtb concentration of 1.5×10^6 CFU/mL.

Initial stock solutions (1000 µg/mL) and subsequent dilutions of test compounds were prepared in DMSO. Further dilutions were performed by twofold dilutions of the stock solution with pure DMSO, i.e. solutions with concentrations of 500, 250, 125, 62.5, 31.25, 15.63, 7.81 µg/mL were obtained. The resulting solutions of the studied compounds (3.1 µL) were loaded into the wells of a 96-well plate, in each of which there was culture medium (97 µL). Then, a suspension of Mtb (100 µL) prepared as described above was added to all wells of the plate. Final concentrations of test compounds in wells of a 96-well plate were 15.6, 7.8, 3.9, 1.95, 0.97, 0.49, 0.24, 0.12 µg/mL; final concentration of DMSO in wells was 1.6 %. A Mtb culture without addition of compounds (100 µL of pure medium + 100 µL of a suspension of Mtb) and a culture of Mtb with DMSO (97 µL of a pure medium + 3.1 µL of DMSO + 100 µL of a suspension of Mtb) were used as a positive control. For control, similar experiments were carried out with isoniazid (initial stock

solution and subsequent dilutions of isoniazid were prepared in water; final concentrations in wells of a 96-well plate were 0.007, 0.015, 0.031, 0.063, 0.125, 0.25, 0.5 μg/mL).

The plates were incubated at 37 °C for 7 days. Then 50 μL of a resazurin (7-hydroxy-3*H*-phenoxazin-3-one 10-oxide) solution (with the addition of Tween 80) was added to the wells and the incubation was continued at 37 °C. The results were monitored after 24, 48 and 72 h. The minimum dilution of the examined compound, in which there was no change in the blue-violet color of resazurin in all replicates, was taken as MIC. All assays were carried out in triplicate.
The results are given in Table 4.

Table 4. Results of anti-TB assay by REMA.

Entry	Compound	MIC (μg/mL)
1	**3a**	–
2	Isoniazid	0.032

Acute toxicity in mice

Adult Balb/C mice (22-28 g) were used for acute toxicity studies (male).

Test animals were kept under vivarium conditions (with natural lighting at 22–24°C and relative humidity 40–50%) on a standard diet (GOST R 50258-92). Experiments were conducted according to good laboratory practice (GLP) rules for preclinical studies in the RF (GOST 3 51000.3-96 and 1000.4-96) and rules of the European Convention for the Protection of Vertebrate Animals Used for Experimental and Other Scientific Purposes (1986). Animals were quarantined for 10–14 d before the experiments.

The test compound **3a** was suspended in a 1% starch solution and administered to the animals orally (per os) at doses of 100, 500, 1000 mg/kg. As an equistress effect, the animals in the control group were administered with 1% starch solution. The animals had unlimited access to water and food.

The results are given in Table 5.

Table 5. Results of the acute toxicity study on the second day of treatment by compound **3a**

Dose, mg/kg	Total number of animals in the group	Number of alive animals	Number of dead animals
100	6	6	0
500	6	6	0
1000	6	6	0
control	6	6	0

Crystal structure determination

The unit cell parameters and the X-ray diffraction intensities were measured on a Xcalibur Ruby diffractometer. The empirical absorption correction was introduced by multi-scan method using SCALE3 ABSPACK algorithm [7]. Using the Olex2 [8], the structures were solved with the SHELXS [9] or SUPERFLIP [10] programs and refined by the full-matrix least-squares method in the anisotropic approximation for all non-hydrogen atoms with the SHELXL program [11]. Hydrogen atoms bound to carbon were located from the Fourier synthesis of the electron density and refined using a riding model. The hydrogen atoms of NH and OH groups were refined independently with isotropic displacement parameters.

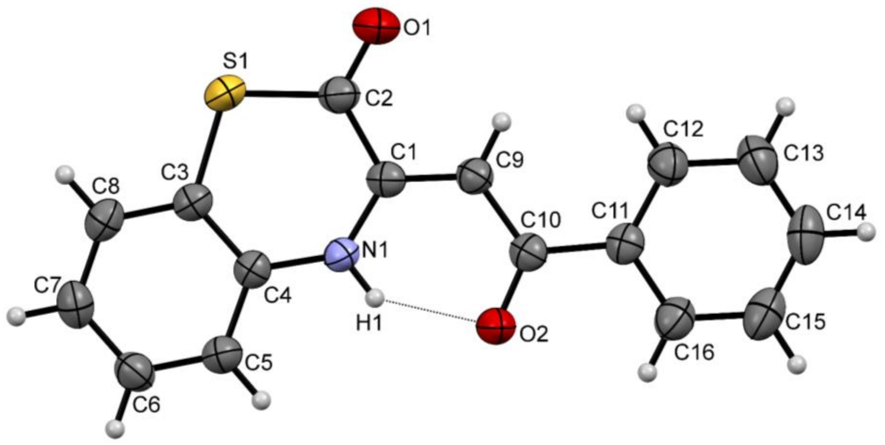

Figure 1. Molecular structure of compound **3a** showing 50% probability amplitude displacement ellipsoids (CCDC 2019498).

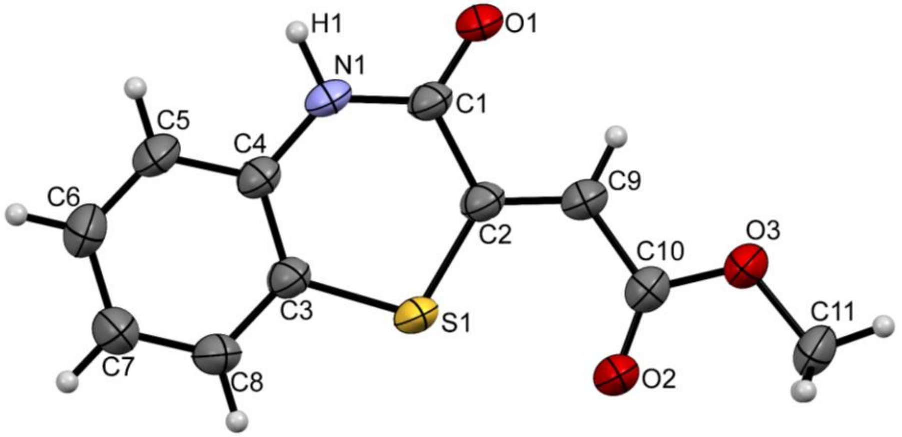

Figure 2. Molecular structure of compound **B** showing 50% probability amplitude displacement ellipsoids (CCDC 2019497).

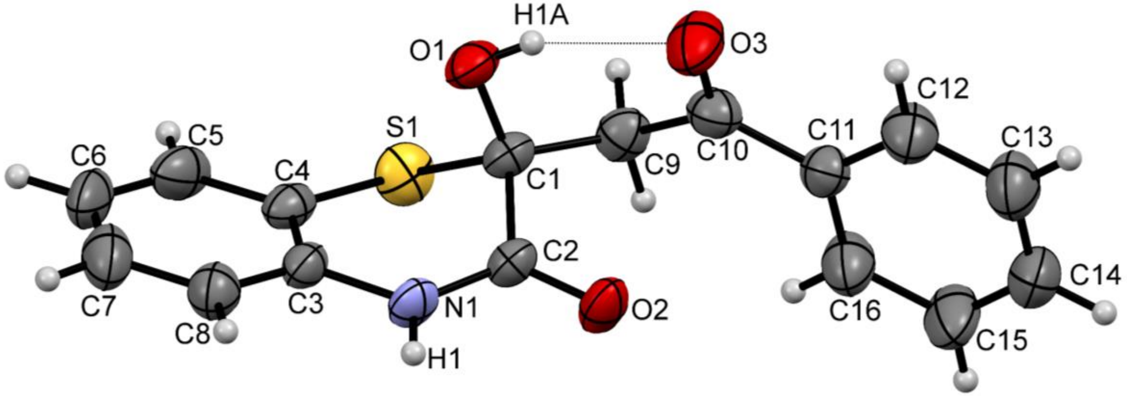

Figure 3. Molecular structure of compound **4a** showing 50% probability amplitude displacement ellipsoids (CCDC 2019501).

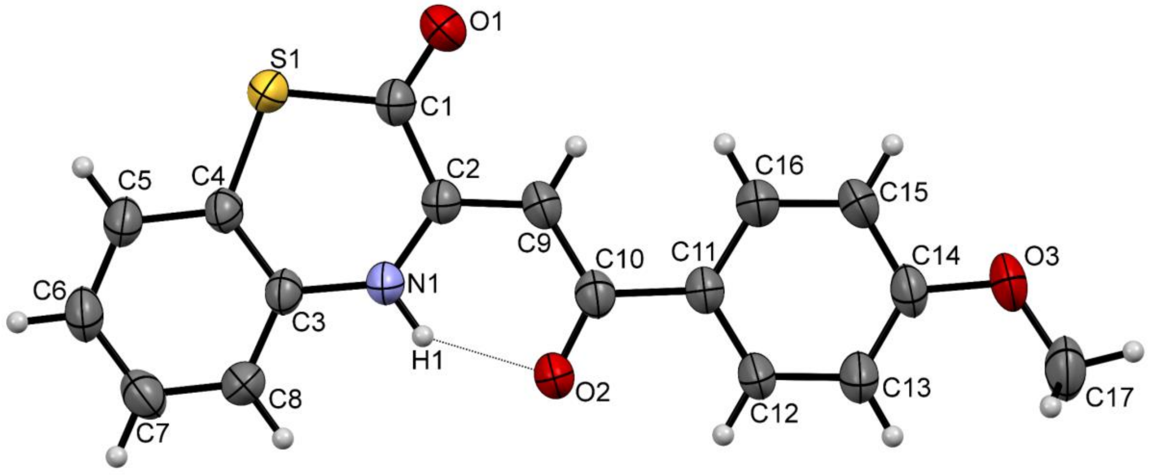

Figure 4. Molecular structure of compound **3c** showing 50% probability amplitude displacement ellipsoids (CCDC 2019500).

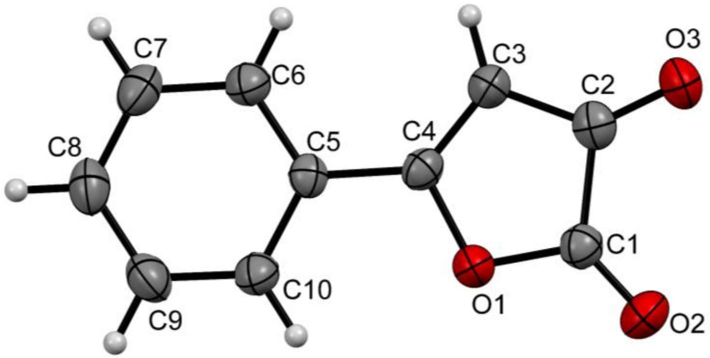

Figure 5. Molecular structure of compound **5a** showing 50% probability amplitude displacement ellipsoids (CCDC 2019499).

Table 6. Crystal data and structure refinement for compound **3a**

Empirical formula	$C_{16}H_{11}NO_2S$
M_r	281.32

Temperature (K)	295
Radiation type, wavelength	Mo*K*α radiation, λ = 0.71073 Å
Scan mode	ω scans
Crystal system, space group	Monoclinic, $P2_1/c$
Unit cell dimensions a, b, c (Å)	12.767(2), 6.3308(13), 16.459(4)
β (°)	100.50(2)
Volume (Å^3)	1308.1(5)
Z	4
Calculated density (g/cm^3)	1.428
Absorption coefficient μ (mm^{-1})	0.247
Crystal size (mm)	0.50 × 0.27 × 0.21
Theta range for data collection	θ_{max} = 29.4°, θ_{min} = 3.2°
Limiting indices	h = −17→13<br>k = −8→5<br>l = −13→22
No. of measured, independent and observed [I > 2σ(I)] reflections	5636, 3022, 2400
R_{int}	0.034
Completeness to θ = 26.0°	0.999
Absorption correction	Empirical absorption correction using spherical harmonics, implemented in SCALE3 ABSPACK scaling algorithm [7].
Min. and max. transmission	0.692, 1.000
Method of structure solution and program	Charge flipping method; SUPERFLIP program [10]
Method of refinement and program	Full-matrix least-squares method on F^2; SHELXL [11]
Treatment of H atoms	Mixed refinement
Data / parameters / restraints	3022 / 185 / 0
Goodness-of-fit on F^2	1.04
Final R indices [I>2 σ (I)]	R_1 = 0.0461, wR_2 = 0.1131

R indices (all data)	R_1 = 0.0600, wR_2 = 0.1268
Largest diff. peak and hole (eÅ^{-3})	0.23, −0.30
CCDC	2019498

Table 7. Crystal data and structure refinement for compound **B**

Empirical formula	$C_{11}H_9NO_3S$
M_r	235.25
Temperature (K)	295
Radiation type, wavelength	Mo*K*α radiation, λ = 0.71073 Å
Scan mode	ω scans
Crystal system, space group	Triclinic, P–1
Unit cell dimensions a, b, c (Å)	5.3770(13), 9.583(2), 10.636(2)
α, β, γ (°)	87.243(19), 79.742(19), 73.87(2)
Volume (Å^3)	518.1(2)
Z	2
Calculated density (g/cm^3)	1.508
Absorption coefficient μ (mm^{-1})	0.30
Crystal size (mm)	0.47 × 0.25 × 0.12
Theta range for data collection	θ_{max} = 29.5°, θ_{min} = 2.9°
Limiting indices	h = −7→7<br>k = −11→12<br>l = −9→14
No. of measured, independent and observed [I > 2σ(I)] reflections	4307, 2407, 1834
R_{int}	0.047
Completeness to θ = 26.0°	0.998
Absorption correction	Empirical absorption correction using spherical harmonics, implemented in SCALE3 ABSPACK scaling algorithm [7]
Min. and max. transmission	0.867, 1.000
Method of structure solution and program	Charge flipping method; SUPERFLIP

	program [10]
Method of refinement and program	Full-matrix least-squares method on F^2; SHELXL [11]
Treatment of H atoms	Mixed refinement
Data / parameters / restraints	2407 / 150 / 0
Goodness-of-fit on F^2	1.04
Final R indices [I>2 σ (I)]	R_1 = 0.0552, wR_2 = 0.1446
R indices (all data)	R_1 = 0.0707, wR_2 = 0.1616
Largest diff. peak and hole (eÅ^{-3})	0.33, −0.45
CCDC	2019497

Table 8. Crystal data and structure refinement for compound **4a**

Empirical formula	$C_{16}H_{13}NO_3S$
M_r	299.33
Temperature (K)	295
Radiation type, wavelength	Mo*K*α radiation, λ = 0.71073 Å
Scan mode	ω scans
Crystal system, space group	Monoclinic, $P2_1/c$
Unit cell dimensions a, b, c (Å)	5.6300(16), 18.757(7), 13.424(6)
β (°)	99.48(3)
Volume (Å^3)	1398.2(9)
Z	4
Calculated density (g/cm^3)	1.422
Absorption coefficient μ (mm^{-1})	0.24
Crystal size (mm)	0.52 × 0.07 × 0.04
Theta range for data collection	$θ_{max}$ = 29.4°, $θ_{min}$ = 3.1°
Limiting indices	h = −7→7<br>k = −25→22<br>l = −17→18
No. of measured, independent and observed [I > 2σ(I)] reflections	6580, 3261, 2037

R_{int}	0.048
Completeness to θ = 26.0°	0.999
Absorption correction	Empirical absorption correction using spherical harmonics, implemented in SCALE3 ABSPACK scaling algorithm [7]
Min. and max. transmission	0.818, 1.000
Method of structure solution and program	Direct method; SHELXS program [9]
Method of refinement and program	Full-matrix least-squares method on F^2; SHELXL [11]
Treatment of H atoms	Mixed refinement
Data / parameters / restraints	3261 / 202 / 0
Goodness-of-fit on F^2	1.03
Final R indices [I>2 σ (I)]	R_1 = 0.0603, wR_2 = 0.1250
R indices (all data)	R_1 = 0.1082, wR_2 = 0.1502
Largest diff. peak and hole (eÅ^{-3})	0.32, −0.24
CCDC	2019501

Table 9. Crystal data and structure refinement for compound **3c**

Empirical formula	$C_{17}H_{13}NO_3S$
M_r	311.34
Temperature (K)	295
Radiation type, wavelength	Mo*K*α radiation, λ = 0.71073 Å
Scan mode	ω scans
Crystal system, space group	Orthorhombic, $Pca2_1$
Unit cell dimensions a, b, c (Å)	30.568(9), 5.9031(19), 8.026(2)
Volume (Å^3)	1448.3(7)
Z	4
Calculated density (g/cm^3)	1.428
Absorption coefficient μ (mm^{-1})	0.24
Crystal size (mm)	0.57 × 0.49 × 0.04
Theta range for data collection	θ_{max} = 29.5°, θ_{min} = 3.5°

Limiting indices	h = −25→38<br>k = −8→7<br>l = −6→11
No. of measured, independent and observed [I > 2σ(I)] reflections	4467, 2473, 2114
R_{int}	0.039
Completeness to θ = 26.0°	0.998
Absorption correction	Empirical absorption correction using spherical harmonics, implemented in SCALE3 ABSPACK scaling algorithm [7]
Min. and max. transmission	0.839, 1.000
Method of structure solution and program	Charge flipping method; SUPERFLIP program [10]
Method of refinement and program	Full-matrix least-squares method on F^2; SHELXL [11]
Treatment of H atoms	Mixed refinement
Data / parameters / restraints	2473 / 204 / 1
Goodness-of-fit on F^2	1.04
Final R indices [I>2 σ (I)]	R_1 = 0.0481, wR_2 = 0.1244
R indices (all data)	R_1 = 0.0578, wR_2 = 0.1367
Largest diff. peak and hole (eÅ^{-3})	0.22, −0.34
CCDC	2019500

Table 10. Crystal data and structure refinement for compound **5a**

Empirical formula	$C_{10}H_6O_3$
M_r	174.15
Temperature (K)	295
Radiation type, wavelength	Mo*K*α radiation, λ = 0.71073 Å
Scan mode	ω scans
Crystal system, space group	Monoclinic, $P2_1/n$
Unit cell dimensions a, b, c (Å)	10.303(5), 5.554(2), 14.014(8)

β (°)	92.14(6)
Volume (Å^3)	801.4(6)
Z	4
Calculated density (g/cm^3)	1.443
Absorption coefficient μ (mm^{-1})	0.11
Crystal size (mm)	0.35 × 0.16 × 0.08
Theta range for data collection	θ_{max} = 29.4°, θ_{min} = 2.4°
Limiting indices	h = −14→14<br>k = −7→7<br>l = −19→18
No. of independent and observed [I > 2σ(I)] reflections	2730, 1593
Completeness to θ = 26.0°	1.000
Absorption correction	Empirical absorption correction using spherical harmonics, implemented in SCALE3 ABSPACK scaling algorithm [7]
Min. and max. transmission	0.350, 1.000
Method of structure solution and program	Direct method; SHELXS program [9]
Method of refinement and program	Full-matrix least-squares method on F^2; SHELXL [11]
Treatment of H atoms	Riding model
Data / parameters / restraints	2730 / 119 / 0
Goodness-of-fit on F^2	0.93
Final R indices [I>2 σ (I)]	R_1 = 0.0564, wR_2 = 0.1393
R indices (all data)	R_1 = 0.0972, wR_2 = 0.1503
Largest diff. peak and hole (eÅ^{-3})	0.22, −0.29
Refined twin ratio	0.7260(17):0.2740(17)
CCDC	2019499

CCDC 2019497-2019501 contain the supplementary crystallographic data for this paper. The data can be obtained free of charge from The Cambridge Crystallographic Data Centre *via* http://www.ccdc.cam.ac.uk.

Partial atomic charges comparison in intermediate XI.

Partial atomic charges of carbonyl carbon atoms in intermediate **XI** (Table 11) were calculated semi-empirically by PM7 method [12] in MOPAC2016 program [13].

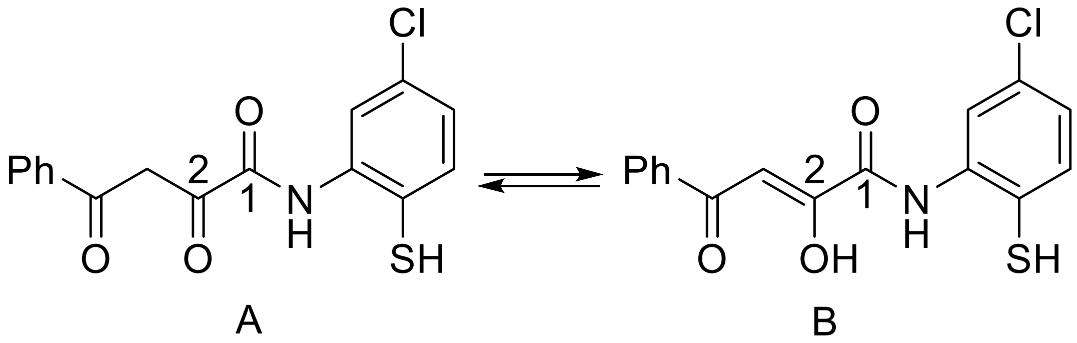

Table 11. Partial atomic charges

Charge	Form A		Form B	
	amide carbonyl atom C1	ketone carbonyl atom C2	amide carbonyl atom C1	ketone carbonyl atom C2
π-charge	0.26027	0.17252	0.26510	0.14387
partial atomic charge	0.555480	0.307158	0.486550	0.414988

References:

1. Beyer, C.; Claisen, L. *Ber. Dtsch. Chem. Ges*., **1887**, *20*, 2178. doi:10.1002/cber.18870200214.

2. Supporting Information to Stepanova, E. E.; Lukmanova, D. N.; Kasatkina, S. O.; Dmitriev, M. V.; Maslivets, A. N. *ChemistrySelect*, **2019**, *4*, 12774. doi:10.1002/slct.201902900.

3. Li, X.; Liu, N.; Zhang, H.; Knudson, S. E.; Slayden, R. A.; Tonge, P. J. *Bioorg. Med. Chem. Lett.*, **2010**, *20*, 6306. doi:10.1016/j.bmcl.2010.08.076.

4. Blaskovich, M. A.; Zuegg, J.; Elliott, A. G.; Cooper, M. A. *ACS Infect. Dis.*, **2015**, *1*, 285. doi:10.1021/acsinfecdis.5b00044.

5. Palomino, J.-C.; Martin, A.; Camacho, M.; Guerra, H.; Swings, J.; Portaels, F. *Antimicrob. Agents Chemother*., **2002**, *46*, 2720. doi:10.1128/AAC.46.8.2720-2722.2002.

6. Taneja, N. K.; Tyagi, J. S. *J. Antimicrob. Chemother*., **2007**, *60*, 288. doi:10.1093/jac/dkm207.

7. CrysAlisPro, Agilent Technologies, Version 1.171.37.33.

8. Dolomanov, O. V.; Bourhis, L. J.; Gildea, R. J.; Howard, J. A. K.; Puschmann, H. *J. Appl. Cryst.*, **2009**, *42*, 339. doi:10.1107/S0021889808042726.

9. Sheldrick, G. M. *ActaCryst*., **2008**, *A64*, 112. doi:10.1107/S0108767307043930.

10. Palatinus, L.; Chapuis, G. *J. Appl. Cryst.,* **2007***, 40*, 786. doi:10.1107/S0021889807029238.

11. Sheldrick, G. M. *ActaCryst*., **2015**, *C71*, 3. doi:10.1107/S2053229614024218.

12. Stewart, J. J. P. *J. Mol. Mod*., **2013**, *19*, 1. doi:10.1007/s00894-012-1667-x.

13. MOPAC2016, Version: 19.059W, James J. P. Stewart, Stewart Computational Chemistry, web-site: HTTP://OpenMOPAC.net.
